# Physiologically Based Pharmacokinetic (PBPK) Modeling of Interstrain Variability in Trichloroethylene Metabolism in the Mouse

**DOI:** 10.1289/ehp.1307623

**Published:** 2014-02-11

**Authors:** Weihsueh A. Chiu, Jerry L. Campbell, Harvey J. Clewell, Yi-Hui Zhou, Fred A. Wright, Kathryn Z. Guyton, Ivan Rusyn

**Affiliations:** 1 National Center for Environmental Assessment, Office of Research and Development, U.S. Environmental Protection Agency, Washington, DC, USA; 2 The Hamner Institutes for Health Sciences, Research Triangle Park, North Carolina, USA; 3 Department of Statistics and Bioinformatics Research Center, North Carolina State University, Raleigh, North Carolina, USA; 4 Department of Environmental Sciences and Engineering, Gillings School of Global Public Health, University of North Carolina at Chapel Hill, Chapel Hill, North Carolina, USA

## Abstract

Background: Quantitative estimation of toxicokinetic variability in the human population is a persistent challenge in risk assessment of environmental chemicals. Traditionally, interindividual differences in the population are accounted for by default assumptions or, in rare cases, are based on human toxicokinetic data.

Objectives: We evaluated the utility of genetically diverse mouse strains for estimating toxicokinetic population variability for risk assessment, using trichloroethylene (TCE) metabolism as a case study.

Methods: We used data on oxidative and glutathione conjugation metabolism of TCE in 16 inbred and 1 hybrid mouse strains to calibrate and extend existing physiologically based pharmacokinetic (PBPK) models. We added one-compartment models for glutathione metabolites and a two-compartment model for dichloroacetic acid (DCA). We used a Bayesian population analysis of interstrain variability to quantify variability in TCE metabolism.

Results: Concentration–time profiles for TCE metabolism to oxidative and glutathione conjugation metabolites varied across strains. Median predictions for the metabolic flux through oxidation were less variable (5-fold range) than that through glutathione conjugation (10-fold range). For oxidative metabolites, median predictions of trichloroacetic acid production were less variable (2-fold range) than DCA production (5-fold range), although the uncertainty bounds for DCA exceeded the predicted variability.

Conclusions: Population PBPK modeling of genetically diverse mouse strains can provide useful quantitative estimates of toxicokinetic population variability. When extrapolated to lower doses more relevant to environmental exposures, mouse population-derived variability estimates for TCE metabolism closely matched population variability estimates previously derived from human toxicokinetic studies with TCE, highlighting the utility of mouse interstrain metabolism studies for addressing toxicokinetic variability.

Citation: Chiu WA, Campbell JL Jr, Clewell HJ III, Zhou YH, Wright FA, Guyton KZ, Rusyn I. 2014. Physiologically based pharmacokinetic (PBPK) modeling of interstrain variability in trichloroethylene metabolism in the mouse. Environ Health Perspect 122:456–463; http://dx.doi.org/10.1289/ehp.1307623

## Introduction

Trichloroethylene (TCE) is an important industrial chemical and a ubiquitous environmental contaminant, and there are complex scientific issues related to its metabolism; the modes, targets, and types of toxicity; and its potential to be a human health hazard. The U.S. Environmental Protection Agency (EPA) and the International Agency for Research on Cancer have concluded that TCE is carcinogenic to humans (Chiu et al. 2013; Guha et al. 2012). Although the cancer hazard classification of TCE has been agreed upon by several expert panels, scientific challenges in the interpretation of the dose–response assessment remain. Major issues include the extent of TCE metabolism through cytochrome P450–mediated oxidation and glutathione *S*-transferase–mediated glutathione conjugation pathways (Lash et al. 2000) in addition to the interindividual differences in the formation of liver- and kidney-toxic metabolites of TCE (Chiu et al. 2009).

Based on the recommendations of the National Research Council (NRC 2006), a physiologically based pharmacokinetic (PBPK) model was used to derive candidate reference dose and concentration values for noncancer human health effects of TCE. A comprehensive PBPK model by Hack et al. (2006) was updated using the Bayesian framework for estimation and characterization of the PBPK model parameter uncertainties (Chiu et al. 2009; Evans et al. 2009). The updated model was used for the dose–response assessment in the U.S. EPA’s TCE toxicological review (U.S. EPA 2011), specifically for quantitative dose extrapolation across routes of exposure, across species, and within species. The latter extrapolation—addressing toxicokinetic variability in the human population—was possible only because of the availability of individual human data on TCE toxicokinetics.

Characterizing variability remains a key risk assessment challenge (Zeise et al. 2013), and there are few chemicals for which sufficient individual human toxicokinetic data are available to conduct population PBPK modeling. Even for TCE, the data are limited to healthy, predominantly male human volunteers largely of European descent. Moreover, the data on glutathione conjugation were much more limited, and questions have been raised as to their reliability for making quantitative estimates of the internal dose. Although it is unlikely that sufficient additional human toxicokinetic data will become available in the future to refine estimates of human toxicokinetic variability, either for TCE or for other chemicals, new experimental approaches using genetically diverse mouse populations offer a potential alternative for evaluating variability. In fact, interstrain differences in TCE metabolism have been quantified using a multistrain panel of inbred mice (Bradford et al. 2011).

TCE offers an attractive case study for examining the utility of the mouse population for characterizing variability. In the present study, we first showed that significant strain and time effects are observed in the metabolism of TCE. Next, we calibrated and further refined PBPK models of TCE (Evans et al. 2009; Hack et al. 2006). We added one-compartment models for *S*-(1,2-dichlorovinyl)glutathione (DCVG) and *S*-(1,2-dichlorovinyl)-l-cysteine (DCVC) and a two-compartment model for dichloroacetic acid (DCA). Finally, we added a population model for interstrain variability to quantify the extent of variability in metabolism through oxidation and glutathione conjugation.

## Materials and Methods

*Animals, treatments, and data availability*. Data used for the analyses presented herein were previously reported (Bradford et al. 2011; Kim et al. 2009b). Additional unpublished data from the study by Bradford et al. (2011) in AKR/J or WSB/EiJs mouse strains are provided in Supplemental Material, Table S1. Males (7–9 weeks of age) from these 16 inbred and 1 hybrid (B6C3F1/J) mouse strains (Jackson Laboratory, Bar Harbor, ME) were gavaged with TCE (2,100 mg/kg) in corn oil (10 mL/kg) and sacrificed at 2, 8, and 24 hr after treatment. Concentrations of DCA, trichloroacetic acid (TCA), DCVG, and DCVC in mouse serum were determined as detailed by Bradford et al. (2011) and Kim et al. (2009a). All studies were conducted with approval of the University of North Carolina at Chapel Hill Institutional Animal Care and Use Committee, and the animals were treated humanely and with regard for alleviation of suffering.

*Analysis of variance (ANOVA) modeling of strain and time effects on concentration–time profiles of TCE metabolites in mouse serum*. Individual animal-level serum TCE metabolite data were examined in a series of power transformations across a grid from 0 (the log transformation) to 1 (untransformed). The transformation y_new = y^0.25 produced the closest average fit to normality across the metabolites, with no influential outliers. Histograms of the transformed values and quantile–quantile plots for each TCE metabolite are shown in Supplemental Material, Figure S1.

ANOVA models were fit to the data with strain as a factor within each time point, and with strain and time point as factors in an overall model, with time point added first to the ANOVA model. An approximate “heritability” was computed as the portion of variation attributable to strain, which was determined using the partial *R*^2^. Statistical tests involving each metabolite were treated as separate hypotheses of independent interest and, thus, not subjected to multiple comparison control.

*Monte Carlo analysis of concentration–time profiles of TCE metabolites in mouse serum*. Monte Carlo analysis of the data was carried out using the TCE PBPK model (Hack et al. 2006) with slight modifications (see Supplemental Material, Figure S2). The model was modified to incorporate the production of DCVG. DCVG clearance was described as metabolism to DCVC. The production of DCA was also altered. In the original model (Hack et al. 2006), DCA was only the product of direct metabolism of TCE. In the modified model, DCA is the product of both the direct metabolism of TCE as well the enzymatic dehalogenation of TCA (Kim et al. 2009b). Model parameters are given in Supplemental Material, Table S2. All other parameters were fixed to the mean posterior value reported by Hack et al. (2006). Monte Carlo analysis was carried out by varying the metabolism and excretion of TCE, TCA, DCA, DCVG, and DCVC while holding all other parameters constant (an approach supported by a sensitivity analysis, discussed below, that confirmed the lack of sensitivity of PBPK model calibration to these parameters). Values for the metabolism were generated randomly from a normal distribution in acslX (Aegis Technologies, Huntsville, AL). The Monte Carlo simulation was run for 100 iterations.

*Model refinement and Bayesian approach to estimating interstrain variability in concentration–time profiles of TCE metabolites in mouse serum*. After completing the preliminary analysis, the additional DCVG, DCVC, and DCA submodels were added to the update by Evans et al. (2009) to the TCE PBPK model of Hack et al. (2006). One-compartment models were used for DCVG and DCVC, and, based on Kim et al. (2009b), a two-compartment model was used for DCA. Complete mathematical details and code are provided in Supplemental Material (Supplemental Material, “Methods,” pp. 32–47, and Supplemental Material—PBPK Model Code).

A hierarchical Bayesian population approach was used, as before, to estimate model parameters and their uncertainty and variability (Bois 2000; Evans et al. 2009; Hack et al. 2006). This involved specification of the hierarchical population statistical model; specification of prior distributions for model and population parameters; estimation of the posterior distributions for model parameters using Markov chain Monte Carlo (MCMC); and evaluation of convergence, the consistency of estimated parameters, and model fit. Parameter scaling relationships and prior distributions, similar to those previously reported by Chiu et al. (2009) and Evans et al. (2009), are provided in Supplemental Material, Tables S3–S6. The likelihood functions used in the Bayesian statistical analysis are described in Supplemental Material, “Methods,” pp. 32–47.

Previously reported population statistical models for TCE PBPK modeling (Chiu et al. 2009; Evans et al. 2009; Hack et al. 2006) did not include variability between mouse strains, and the analyses only characterized variability between studies. Because most of the previously reported data available for PBPK modeling involved only the B6C3F1 strain, most of this interstudy variability was due to variation in laboratory conditions or among studies. In order to separately characterize variation between strains, the following approach was used. For studies other than Bradford et al. (2011), only data using the B6C3F1 strain were included. The B6C3F1 data (Kim et al. 2009b) were excluded for the study by Bradford et al. (2011). Interstudy variability (θ) in PBPK model parameters was characterized using a population model, and included for all studies. A population model for interstrain variability was constructed by adding interstrain scaling parameters (ψ) that are equal to the ratio between the PBPK model parameter for a specific strain and the PBPK model parameter for the B6C3F1 strain. Prior distributions for interstrain variability are provided in Supplemental Material, Table S5. All other aspects of the population statistical model were as reported previously (Chiu et al. 2009; Evans et al. 2009).

Sensitivity analyses reported by the U.S. EPA (2011) showed that the PBPK model calibration was not sensitive to many parameters. Therefore, most of the physiological parameters and partition coefficients for which there were *in vitro* estimates available were fixed to their baseline values. The remaining parameters were estimated and evaluated using the previously reported approach (Chiu et al. 2009; Evans et al. 2009).

## Results

*ANOVA modeling of strain and time effects on serum concentration–time profiles of TCE metabolites*. The TCE metabolite data were examined for evidence of strain and time effects using a fixed-effect two-way ANOVA model, with partial *R*^2^ used to describe the portion of variability attributable to “strain” and “time.” For strain effects, the partial *R*^2^ may be viewed as serving as an index of heritability, although this term is used here in an approximate sense, due to the nonrandom sampling of strains. The overall effects of strain in the two-way model (Table 1) were highly significant for DCVG (*p* = 9 × 10^–5^), and not significant for TCA, DCA, and DCVC. Time effects were significant for TCA, DCA, and DCVG, but not significant for DCVC. Overall, concentration–time profiling in a multistrain experimental design illustrated the importance of both strain and time on TCE metabolite concentrations. The heritability (partial *R*^2^ attributable to strain) estimates ranged from 0.18 to 0.49 for all time periods (Table 1).

**Table 1:** Results of ANOVA modeling of the effect of time and strain on TCE metabolite concentrations in mouse serum.

Metabolite	Time point (hr)	Sample size (*n*)	Partial *R*^2^ (“heritability”)	Strain		Time	
				*F*-statistic	*p*-Value	*F*-statistic	*p*-Value
TCA	2	37	0.25	0.53	0.890
	8	36	0.63	2.51	0.028
	24	23	0.76	2.65	0.066
	All		0.18	1.24	0.263	59.00	< 1 × 10^–10^
DCA	2	38	0.56	2.13	0.052
	8	36	0.58	2.09	0.061
	24	25	0.78	2.53	0.073
	All		0.22	1.69	0.075	6.19	0.003
DCVG	2	36	0.82	6.62	0.00001
	8	33	0.89	8.88	< 1 × 10^–10^
	24	12	1.00	1,376	0.021
	All		0.49	3.75	0.00009	40.50	< 1 × 10^–10^
DCVC	2	32	0.60	1.83	0.118
	8	19	0.72	1.27	0.404
	24	8	0.74	0.48	0.801
	All		0.34	1.41	0.189	1.53	0.229

**Figure 1:**
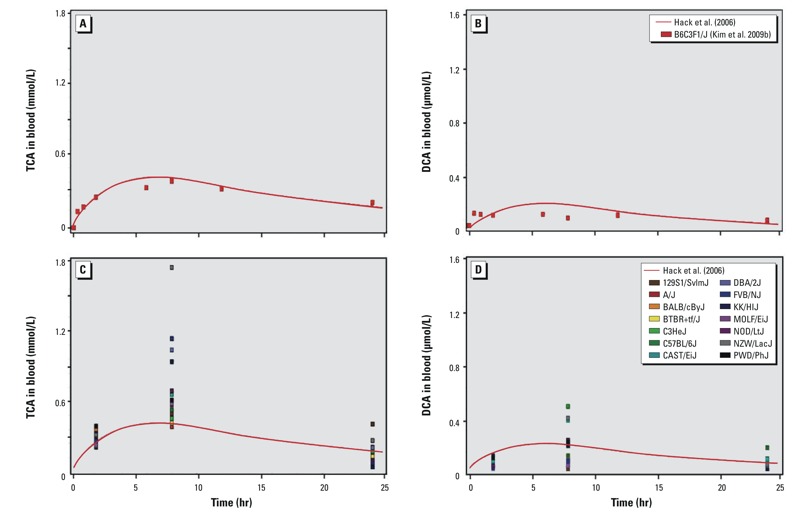
Hack et al. (2006) TCE PBPK model simulation of TCA and DCA compared to the measured data from Kim et al. (2009b) for the B6C3F1/J strain (*A*,*B*), which was used in the original model development and compared to data from a panel of inbred mouse strains (*C*,*D*) from Bradford et al. (2011).

*Interstrain variability in serum concentration–time profiles of TCE metabolites*. We examined how well the Hack et al. (2006) TCE PBPK model corresponds to the concentration–time profiles of oxidative TCE metabolites in serum of B6C3F1/J mice, the strain used in developing this model. A good fit was observed for the time-course TCA and DCA concentrations (Figure 1A,B). When compared with the kinetic data across the strains (Figure 1C,D), the B6C3F1/J strain showed a peak concentration of TCA near the bottom of the distribution at 2 and 8 hr after dosing and fell near the middle of the distribution at 24 hr (Figure 1C). For DCA, the B6C3F1/J strain was above the distribution of plasma concentration at 2 hr and fell near the middle at 8 and 24 hr after dosing (Figure 1D). The Monte Carlo analysis of the multistrain data (see Supplemental Material, Figure S3) using the modified Hack et al. (2006) model was reasonably consistent with the range of measured concentrations of TCA at 8 and 24 hr, and most measured values were below the distribution at 2 hr. For DCA, the simulations overpredicted the observed data by about a factor of two. The spread of measured concentrations for DCVG were captured by the Monte Carlo analysis at 2 hr, but with approximately 50% of the strains falling below the distribution of the simulations. The model failed to capture the rapid clearance of DCVG with all of the measured concentrations at 8 hr falling below the simulations. For DCVC, however, the Monte Carlo simulation was able to reasonably capture both the distribution and shape of the measured data for most strains at all three time-points.

**Figure 2:**
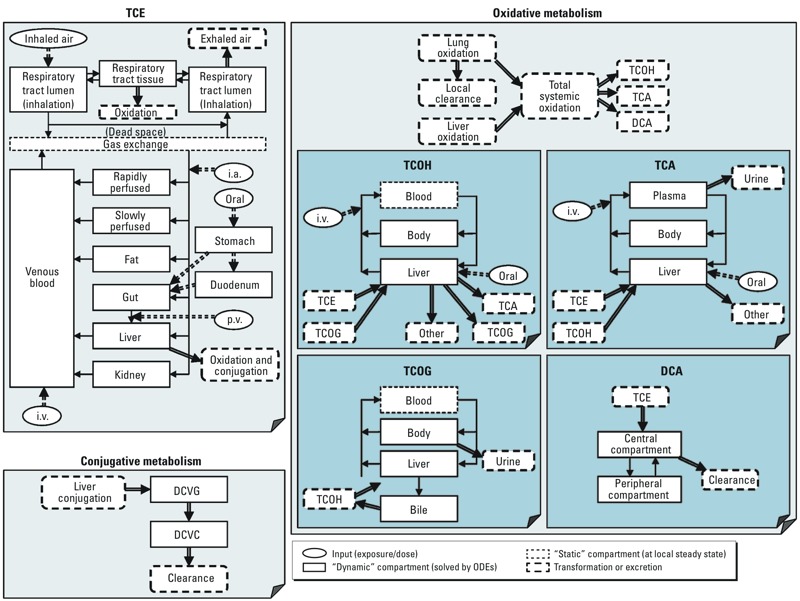
Schematic of the mouse PBPK model of TCE and its metabolites after model refinement, used for Bayesian estimation of interstrain variability. Abbreviations: i.a., intraarterial injection; i.v., intravenous injection; p.v., portal venous injection. The image has been modified from Chiu et al. (2009).

*Model refinement and Bayesian estimates of interstrain variability in serum concentration–time profiles of TCE metabolites*. Because the Hack et al. (2006) model and Monte Carlo simulations did not adequately capture the extent of interstrain variability in serum concentration–time profiles of TCE metabolites, we conducted additional model refinements using the Evans et al. (2009) update to the Hack et al. (2006) model (Figure 2), and performed Bayesian population modeling. Physiological models were added for TCA and trichloroethanol (TCOH), and a 2-compartment model was added for DCA. For the Bayesian population modeling, we ran eight independent MCMC chains, each to 160,000 iterations, with the first half discarded as “burn-in” iterations. Values of the convergence diagnostic “*R*” were < 1.07 for all parameters, indicating convergence (a < 7% change would be expected with further simulation). Only every 500th iteration was retained to reduce autocorrelation. Therefore, a total of 1,280 parameter samples [(8 × 80,000)/500)] were available for analysis.

Posterior distributions are summarized in Supplemental Material, Table S6. Posterior distributions for the previously developed TCE, TCA, and TCOH/TCOG (trichloroethanol glucuronide) submodels were consistent with the analyses of Chiu et al. (2009) and Evans et al. (2009). All posteriors were well within the truncation range of the priors, so the priors were not overly constraining. Furthermore, the data appeared to be informative as to the parameters for the new DCVG, DCVC, and DCA submodels, as evidenced by the posteriors being significantly narrower than the priors.

**Figure 3:**
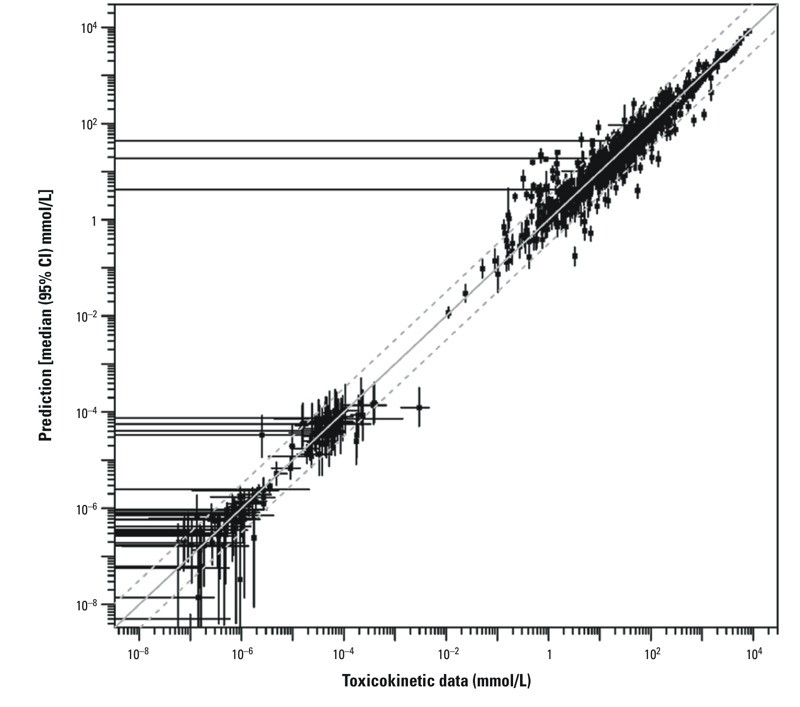
Global evaluation of model fit, comparing toxicokinetic data (x-axis) and PBPK model predictions (y-axis), each with 95% CIs (error bars). In some cases, the confidence interval on the data included 0, as indicated by horizontal error bars that extend all the way to the left. The solid gray diagonal line indicates where data and predictions are equal, and the dashed lines indicate where they are within 3-fold.

**Figure 4:**
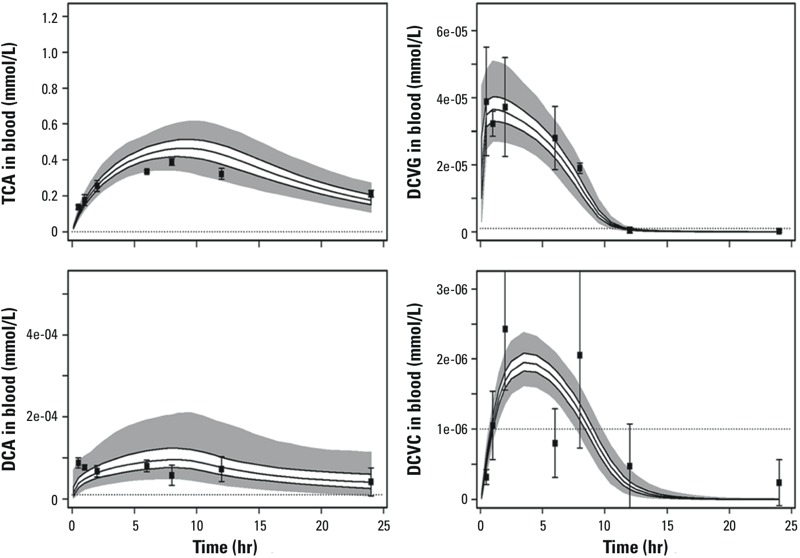
Comparison of data (data points with ± 1 SD error bars) and PBPK model predictions (solid lines, interquartile range; gray area, 95% CI) for TCE metabolites in B6C3F1 mice (data from Kim et al. 2009b). Dotted lines indicate the limits of detection.

**Figure 5:**
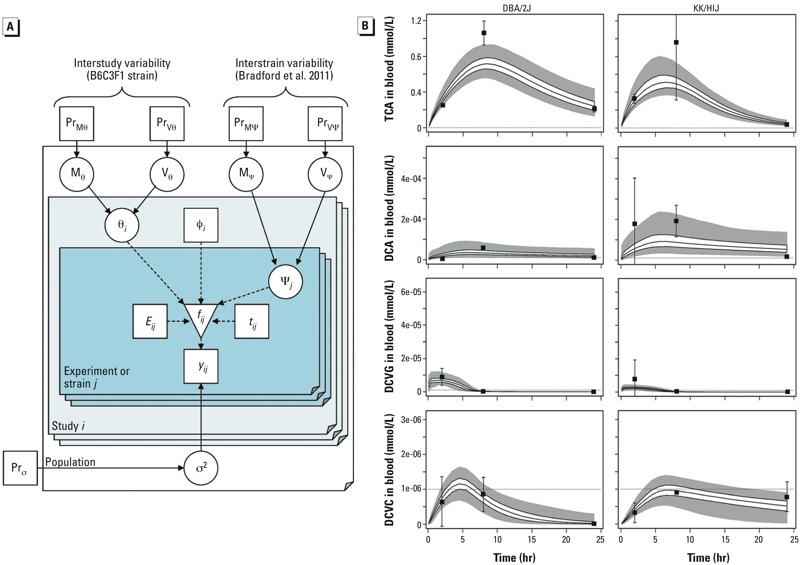
(*A*) Hierarchical population statistical model for PBPK model uncertainty and variability. Square nodes represent fixed or observed quantities, circle nodes represent uncertain or unobserved quantities, and the inverted triangle represents PBPK model outputs; solid arrows indicate a stochastic relationship represented by a conditional distribution [e.g., A→B means B ~ P(B|A)], whereas dashed arrows indicate a functional relationship [e.g., B = *f*(A)]. The population consists of studies *i*, each of which contains experiments or strains *j*, with exposure parameters *E_ij_*, and data *y_ij_* collected at times *t_ij_*. The PBPK model produces outputs *f_ij_*. The difference between data and predictions is assumed to have a distribution with variance σ^2^, which is assigned a prior distribution (Pr). The PBPK model uses non–strain-specific parameters θ_i_, measured covariates φ_i_, and strain-specific parameters ψj. The parameters are each drawn from population distributions with mean M_θ or ψ_ and variance V_θ or ψ_, each of which are in turn assigned prior distributions. (*B*) Comparison of data (data points with ± 1 SD error bars) and PBPK model predictions (solid lines represent interquartile range, gray area represents 95% CI) for two representative mouse inbred strains (data from Bradford et al. 2011). Dotted lines indicate the limits of detection.

Figure 3 demonstrates an overall comparison of model predictions and observed data, showing that the majority of predictions are within 3-fold of the data. Individual time-courses are provided in Supplemental Material, Figures S4–S7, with predictions for the B6C3F1/J strain (Kim et al. 2009b) and two representative inbred strains DBA/2J and KK/HIJ (Bradford et al. 2011) depicted in Figures 4 and 5, respectively. The most influential model refinements leading to improved predictions were the use of a two-compartment model for DCA and the change in glutathione-related parameters—specifically, both increased production and increased clearance of DCVG.

Overall, model predictions are consistent with metabolism of TCE occurring predominantly by oxidation compared with glutathione conjugation, and with more TCA produced from oxidation compared with DCA. Estimates of metabolism parameters and metabolic fluxes for the B6C3F1/J mice are shown in Table 2.

**Table 2:** TCE metabolism parame­ters for B6C3F1/J strain: median (2.5%, 97.5%) of posterior distribution.

Parameter or prediction	Abbreviation	Value
*V*_max_ for liver oxidation (mg/hr)	VMax	2.1 (0.73, 5.6)
*K*_m_ for liver oxidation (mg/L)	KM	3.3 (0.63, 19)
*V*_max_ for liver GSH conjugation (mg/hr)	VMaxDCVG	0.006 (0.003, 5.9)
*K*_m_ for liver GSH conjugation (mg/L)	KMDCVG	0.06 (0.003, 9.8 × 10^4^)
*V*_max_/*K*_m_ for liver GSH conjugation (L/hr)	Vmax/KM	0.1 (4 × 10^–5^, 2.1)
Dose (mg) [fixed]		76.4
Amount of TCE metabolized (mg)	AMetOx	16 (5.5, 60)
Amount of TCE conjugated (mg)	AMetGSH	0.05 (0.03, 0.5)
Amount of TCA produced (mg)	TotTCAProd	3.4 (1.4, 15)
Amount of DCA produced (mg)	TotDCAProd	0.3 (0.02, 3.7)
Oxidation/GSH ratio (mg TCE oxidized/mg TCE conjugated)	OXtoGSHRatio	290 (41, 1,070)
TCA/DCA ratio (mmol TCA produced/mmol DCA produced)	TCAtoDCAratio	10 (0.9, 130)
Abbreviations: GSH, glutathione; *V*_max_, maximum reaction velocity.		

**Figure 6:**
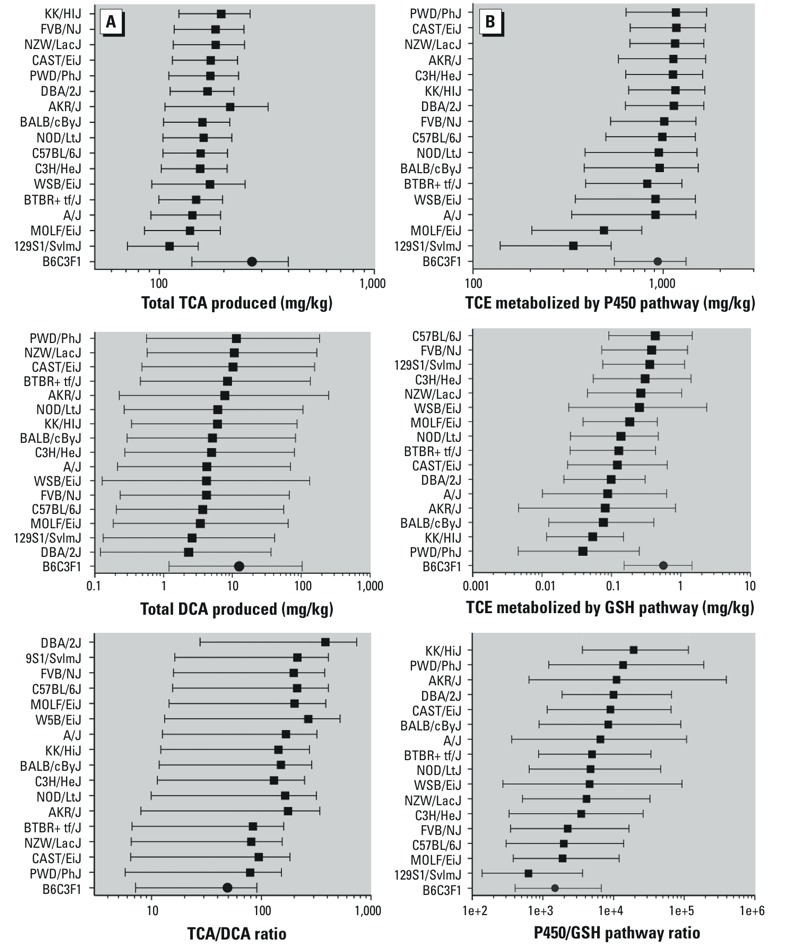
Predictions for TCE metabolites and metabolite fluxes across mouse strains (median and 95% CI). (*A*) TCA produced, DCA produced, and the ratio of TCA/DCA produced. (*B*) Flux of TCE metabolism through oxidation, flux through glutathione conjugation, and ratio of oxidation to glutathione conjugation. A solid circle is used for the “index” B6C3F1 strain, whereas solid squares are used for the other strains.

Figure 6 shows PBPK model predictions for the overall flux of TCE metabolism across mouse strains. Figure 6A shows that less interstrain variability is predicted for TCA (a 2-fold range) than DCA (a 5-fold range), although the uncertainty bounds for DCA were wider than the predicted range of variability. All strains were estimated to produce significantly more TCA than DCA; median estimates for their ratio varied from 11 to 53 (a 5-fold range of variability). Compared to B6C3F1/J, median predictions for most other strains estimated less TCA and DCA production, but with a higher TCA/DCA ratio. Figure 6B shows results for the oxidative and glutathione conjugation pathways. Less variation was predicted for oxidative metabolism (5-fold range across strains) compared with glutathione conjugation (10-fold). Interestingly, in terms of total oxidative metabolism, all but two strains (MOLF/EiJ and 129S1/SvlmJ) were within 2-fold of each other, probably a result of blood-flow–limited metabolism. The two “outlier” strains were predicted to have notably less flux through this pathway. The B6C3F1/J strain was predicted to have more glutathione metabolism than other strains, and median estimates for the oxidation/conjugation ratio was lower than all but the 129S1/SvlmJ strain. Still, all strains were estimated to have a greater metabolic flux through oxidation compared with glutathione conjugation; median estimates for their ratio varied about 30-fold (from 620 to 19,000).

## Discussion

One of the biggest gaps in risk assessment, as identified by the NRC (2009), is that interindividual variability is not being addressed at all (in animals), or incompletely (in epidemiological studies). There is a crucial need for the development of approaches to estimate the quantitative impact of human interindividual variability in personal risk from chemical exposures (Zeise et al. 2013), and with adequate human data, a number of statistical and computational tools are available to toxicologists and risk assessors (Dorne et al. 2012).

However, there are no experimental data with which to derive such population distributions for most toxicants. Some studies have been performed using data on pharmaceuticals (Hattis and Lynch 2007), but the variability in individual responses to drugs—which have generally similar pharmacokinetic properties—is unlikely to encompass the extent of variability in responses to environmental agents (Clewell et al. 2004). Epidemiological data are also of limited use because the variation in response is confounded by the variability in exposure. Combined *in vitro* and computational approaches have been proposed to characterize toxicokinetic variability (Wetmore et al. 2013), but these are limited to first-order kinetics and characterization of variability in parent compound dosimetry. Other *in vitro* approaches to evaluating the extent of and molecular mechanisms for interindividual variability using genetically diverse cell lines have also been proposed (Lock et al. 2012; O’Shea et al. 2011). However, these and other *in vitro* approaches that do not capture the complexity of whole body toxicokinetics would not be successful for compounds such as TCE. Indeed, the metabolism of TCE is complex, with multiple metabolizing tissues and interorgan transport, and toxicity is largely attributed to metabolites rather than the parent compound. As a consequence of these data limitations, current approaches are largely limited to applying default uncertainty factors to account for uncertainty associated with within-species variability (Stedeford et al. 2007).

One possible way to fill this gap is by characterizing the nature and quantitative extent of human variability through studies in the mouse model of the human population (Rusyn et al. 2010). Accordingly, we hypothesized that by using data from a mouse population we can build kinetic models to account for interindividual variability in metabolism from the point of view of genetic variability. Specific focus was on PBPK modeling to generate information and kinetic parameters that may be used for verifying the models used in TCE risk assessment (Evans et al. 2009; Hack et al. 2006). In addition, a Bayesian modeling approach was used for uncertainty and sensitivity analysis (Chiu et al. 2009).

We found considerable variability in TCE metabolism across mouse strains (Bradford et al. 2011) and our novel analytical techniques offer data on additional key metabolites (Kim et al. 2009b) that were used to extend existing TCE PBPK models. Whereas the Hack et al. (2006) model accurately describes the kinetics of TCA in the B6C3F1/J mouse, we found that it only partially (mostly at the lower range) accounts for the variability in the toxicokinetics of TCE observed in a genetically diverse population of mouse strains. A hierarchical Bayesian approach was more successful in estimating the population variability. Using this approach, variability in the rate of production of metabolites (TCA, DCA, DCVG) was seen across strains. All strains were predicted to have a greater metabolic flux through oxidation compared with glutathione conjugation, but with 31-fold variability in the ratio across strains (Figure 6B). Although most strains had predicted total oxidative metabolism within a narrow 2-fold range (likely a result of blood-flow–limited metabolism), two strains were predicted to have notably less metabolism by this pathway. The metabolic flux through glutathione conjugation had a greater range of variability (10-fold) across strains.

These results have a number of limitations. First, the confidence intervals in some cases are quite wide, particularly for DCA. Because there is some confounding between a low rate of production and rapid clearance of DCA (both of which could account for the low levels of DCA in blood), DCA dosing would undoubtedly reduce the associated uncertainty. In addition, the predominant oxidative metabolite is TCOH, which was not measured in our studies. Thus, estimating the balance of oxidation to TCOH in these strains relied on information from previous studies of B6C3F1 mice, which introduces uncertainty due to interstudy variation. Finally, many measurements of DCA, DCVC, and DCVG were near the limit of detection, where analytical errors are larger, so the precision was limited by experimental variation.

We also posit that the “mouse variability distribution” may be further extrapolated to humans using the PBPK model and the resulting human variability distribution may be compared with available data on the variability of the human pharmacokinetics of TCE to determine whether the mouse-derived distribution is consistent with the human evidence. Because of previous work developing human population PBPK models (Bois 2000; Chiu et al. 2009; Hack et al. 2006) a direct comparison is possible, for instance, between the extent of population variability predicted in the human population based on individual human data, and that predicted in a mouse based on multiple strain data. As shown in Table 3, where the ratio of 95th percentile and median in humans were compared with those for mouse strains, there was a remarkable correspondence between the predictions when evaluated at low doses, which are more relevant to environmental exposures. Specifically, both the mouse- and human-based analyses predicted the general trend of the lowest variability in oxidative metabolism (about 1.1-fold between the 95th percentile and the median), greater variability in TCE productions (about 2-fold), and the greatest variability in glutathione conjugation (about 7-fold). Moreover, central estimates were within 20% of each other, with the confidence intervals based on mouse data completely encompassing those based on human data. The difference in confidence intervals may simply reflect the larger number of individuals in the human analysis (*n* = 42) compared with the number of mouse strains (*n* = 17). The combination of using a PBPK model, data from the population-wide experimental model, and statistically rigorous parameter estimation gives this approach its predictive power. Overall, the results reported here based on interstrain variability in mice are consistent with estimates derived from previously published analyses based on individual human data.

**Table 3:** Comparison of Chiu et al. (2009) human variability predictions for TCE metabolism with variability predictions for TCE metabolism among mouse strains. Ratios of 95th percentile/50th percentile individual or strain are shown. Median estimate and 95% CI were calculated at an oral dose of 0.001 mg/(kg-day), where non­linearities in toxicokinetics are negligible.

Parameter	Human inter­individual variability (Chiu et al. 2009)	Mouse interstrain variability (present analysis)
TCE oxidized by P450	1.11 (1.05, 1.22)	1.05 (1.01, 1.27)
Total TCA produced	2.09 (1.81, 2.51)	1.77 (1.36, 2.99)
TCE conjugated with glutathione	6.61 (3.95, 11.17)	7.12 (3.43, 20.66)

## Conclusions

The present case study demonstrates the feasibility of using mouse population models to characterize the nature and extent of human interindividual variability in pharmacokinetics for toxicologically relevant measures of internal dose—a similar approach that could be applied to other chemicals. Because characterization of pharmacokinetic variability is a necessary precursor to characterization of pharmacodynamic variability, this work considerably extends the risk assessment utility of PBPK-modeling tools and Bayesian methods for analysis of population-wide data, having both immediate impact and future translational potential.

## Supplemental Material

(3.9 MB) PDF

(270 KB) PDF
