# Supplementary material for: Physiologically Based Pharmacokinetic (PBPK) Modeling of Interstrain Variability in Trichloroethylene Metabolism in the Mouse
Source: Environ Health Perspect. 2014 Feb 11;122(5):456–63. doi: 10.1289/ehp.1307623 (PMC4014769; doi:10.1289/ehp.1307623)
Supplement: (3.9 MB) PDF [file ehp.1307623.s001.pdf]

Supplemental Material

Physiologically Based Pharmacokinetic (PBPK) Modeling of Interstrain Variability in Trichloroethylene Metabolism in the Mouse

Weihsueh A. Chiu, Jerry L. Campbell Jr., Harvey J. Clewell 3rd, Yi-Hui Zhou, Fred A. Wright, Kathryn Z. Guyton, and Ivan Rusyn

Table of Contents **Page**

Figure S1. Histograms of the transformed values and quantile-quantile plots for TCE metabolites TCA, DCA, DCVG and DCVC. **3**

Figure S2. Schematic of the modified Hack et al. (2006) model. **4**

Figure S3. Monte Carlo simulation (100 iterations) of the multistrain mouse time-course data with the parameter distributions for metabolism and clearance of metabolites estimated to fit the range of measured values. **5**

Figure S4. Comparison of data and PBPK model predictions for TCA in mouse inbred strains. **6**

Figure S5. Comparison of data and PBPK model predictions for DCA in mouse inbred strains. **8**

Figure S6. Comparison of data and PBPK model predictions for DCVG in mouse inbred strains. **10**

Figure S7. Comparison of data and PBPK model predictions for DCVC in mouse inbred strains. **12**

Table S1. TCE metabolite data for individual mice (rows) of AKR/J and WSB/EiJ strains. **14**

Table S2. Parameter distributions estimated with Monte Carlo analysis of the multistrain mouse data. **15**

Table S3. PBPK model parameters, baseline values, and scaling relationships. **16**

Table S4. Uncertainty distributions for the population mean and variance of the PBPK model parameters. **25**

Table S5. Interstrain variability parameters. **29**

Table S6. Comparison of prior and posterior distributions for scaling parameters. **30**

Methods **32**
Details of the Bayesian PBPK modeling of TCE and its metabolites 32
PBPK model structure and equations 32
TCE submodel 33
Gas exchange, respiratory metabolism, arterial blood concentration, and closed-chamber concentrations 33
Oral absorption to gut compartment 35
Nonmetabolizing tissues 36
Liver compartment 36
Venous blood compartment 37
TCOH Submodel 38
Blood concentration 38
Body compartment 38
Liver compartment 39
TCOG Submodel 40
Blood concentration 40
Body compartment 41
Liver compartment 41
Bile compartment 41
TCA Submodel 42
Plasma binding and concentrations 42
Urinary excretion 43
Body compartment 44
Liver compartment 44
GSH conjugation submodel 45
DCA submodel 45
Likelihood function 46

References **48**

Figure S1. Histograms of the transformed values and quantile-quantile plots for TCE metabolites TCA, DCA, DCVG and DCVC. The transformation y_new=y^0.25 produced the closest average fit to normality across the metabolites, with no influential outliers.

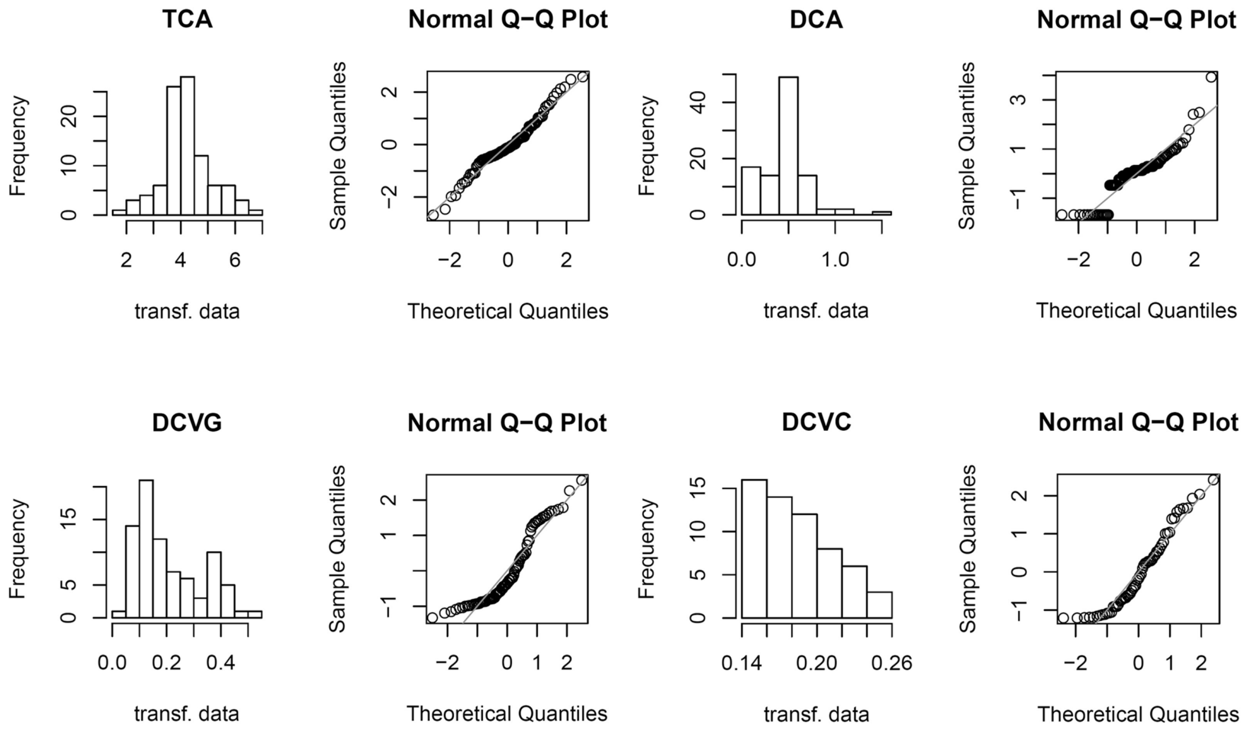

Figure S2. Schematic of the modified Hack et al. (2006) model. Reprinted with permission from Elsevier.

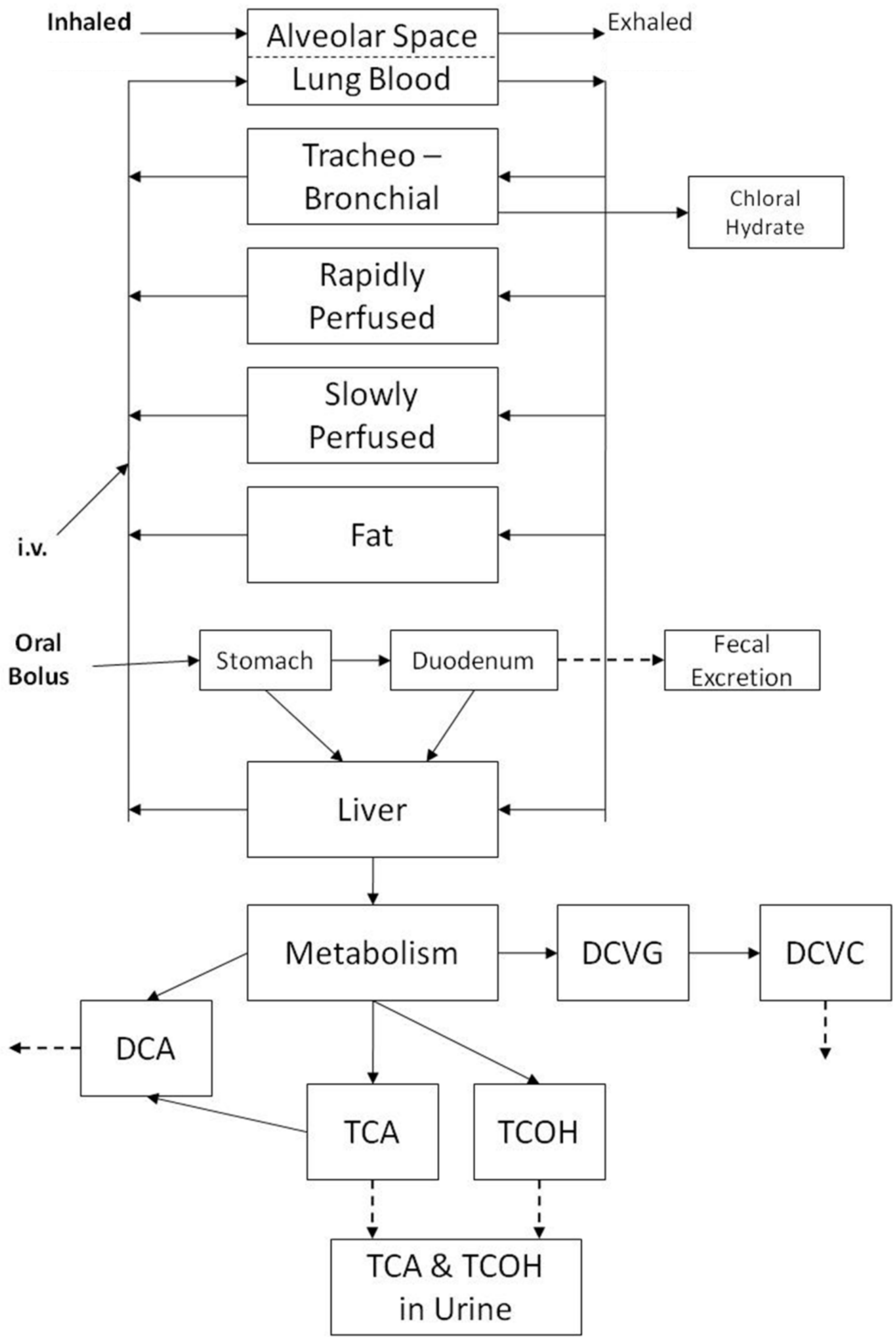

Figure S3. Monte Carlo simulation (100 iterations) of the multistrain mouse time-course data with the parameter distributions for metabolism and clearance of metabolites estimated to fit the range of measured values.

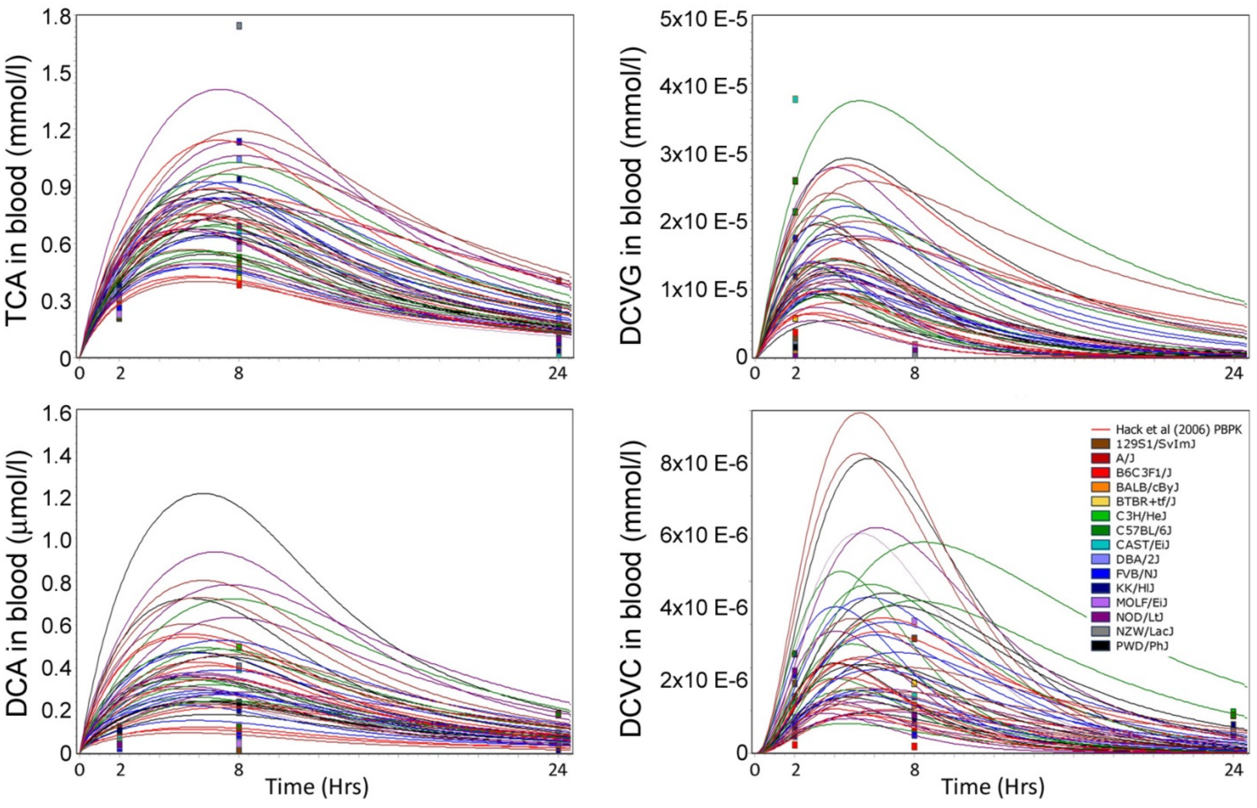

Figure S4. Comparison of data (solid boxes with ± 1 SD error bars) and PBPK model predictions (solid lines: interquartile range; grey area: 95% confidence interval) for TCA in mouse inbred strains [data from (Bradford et al. 2011)].

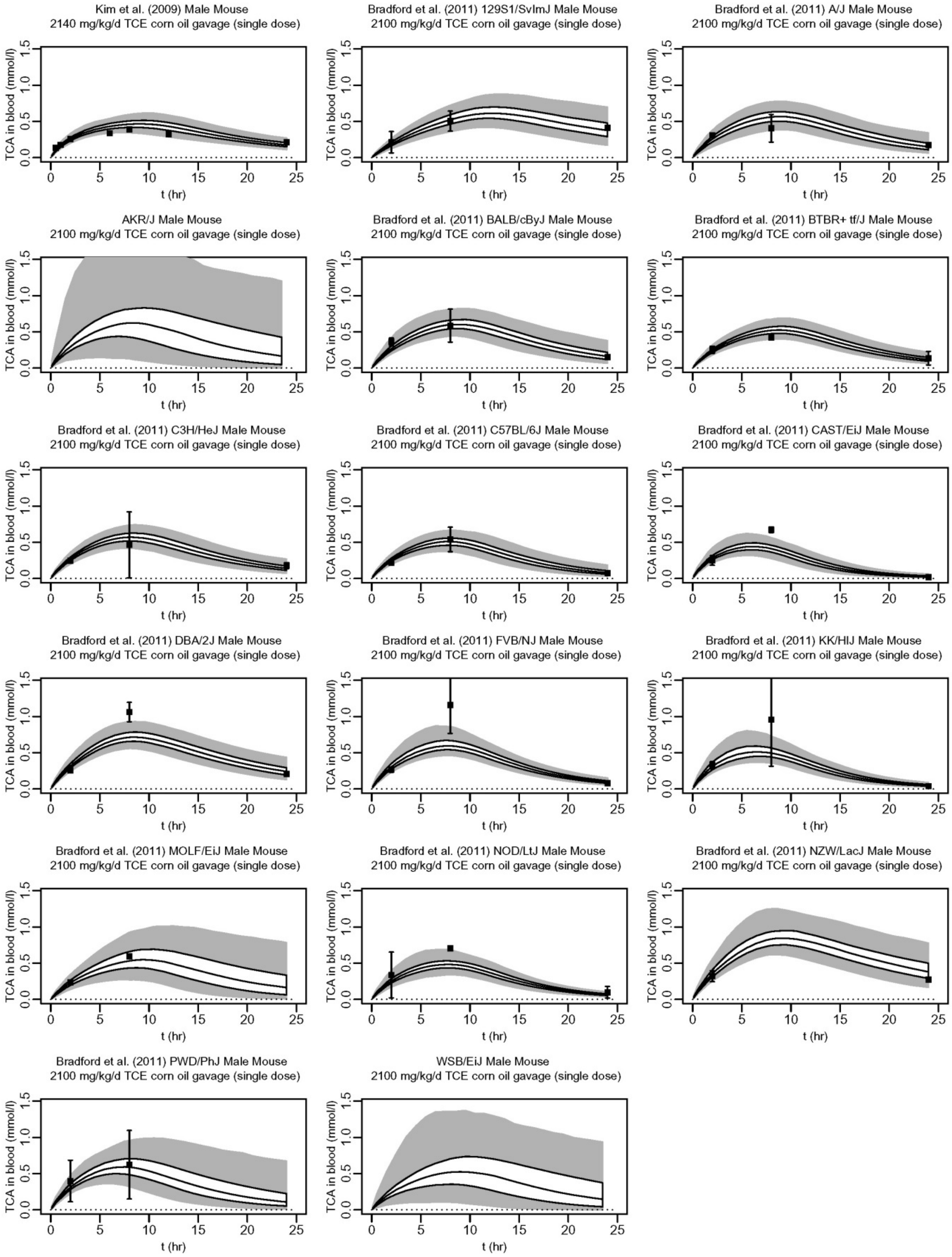
Kim et al. (2009) Male Mouse
2140 mg/kg/d TCE corn oil gavage (single dose)
Bradford et al. (2011) 129S1/SvImJ Male Mouse
2100 mg/kg/d TCE corn oil gavage (single dose)
Bradford et al. (2011) A/J Male Mouse
2100 mg/kg/d TCE corn oil gavage (single dose)
AKR/J Male Mouse
2100 mg/kg/d TCE corn oil gavage (single dose)
Bradford et al. (2011) BALB/cByJ Male Mouse
2100 mg/kg/d TCE corn oil gavage (single dose)
Bradford et al. (2011) BTBR+ tf/J Male Mouse
2100 mg/kg/d TCE corn oil gavage (single dose)
Bradford et al. (2011) C3H/HeJ Male Mouse
2100 mg/kg/d TCE corn oil gavage (single dose)
Bradford et al. (2011) C57BL/6J Male Mouse
2100 mg/kg/d TCE corn oil gavage (single dose)
Bradford et al. (2011) CAST/EiJ Male Mouse
2100 mg/kg/d TCE corn oil gavage (single dose)
Bradford et al. (2011) DBA/2J Male Mouse
2100 mg/kg/d TCE corn oil gavage (single dose)
Bradford et al. (2011) FVB/NJ Male Mouse
2100 mg/kg/d TCE corn oil gavage (single dose)
Bradford et al. (2011) KK/HIJ Male Mouse
2100 mg/kg/d TCE corn oil gavage (single dose)
Bradford et al. (2011) MOLF/EiJ Male Mouse
2100 mg/kg/d TCE corn oil gavage (single dose)
Bradford et al. (2011) NOD/LtJ Male Mouse
2100 mg/kg/d TCE corn oil gavage (single dose)
Bradford et al. (2011) NZW/LacJ Male Mouse
2100 mg/kg/d TCE corn oil gavage (single dose)
Bradford et al. (2011) PWD/PhJ Male Mouse
2100 mg/kg/d TCE corn oil gavage (single dose)
WSB/EiJ Male Mouse
2100 mg/kg/d TCE corn oil gavage (single dose)
TCA in blood (mmol/l)
t (hr)

Figure S5. Comparison of data (solid boxes with ± 1 SD error bars) and PBPK model predictions (solid lines: interquartile range; grey area: 95% confidence interval) for DCA in mouse inbred strains [data from (Bradford et al 2011)].

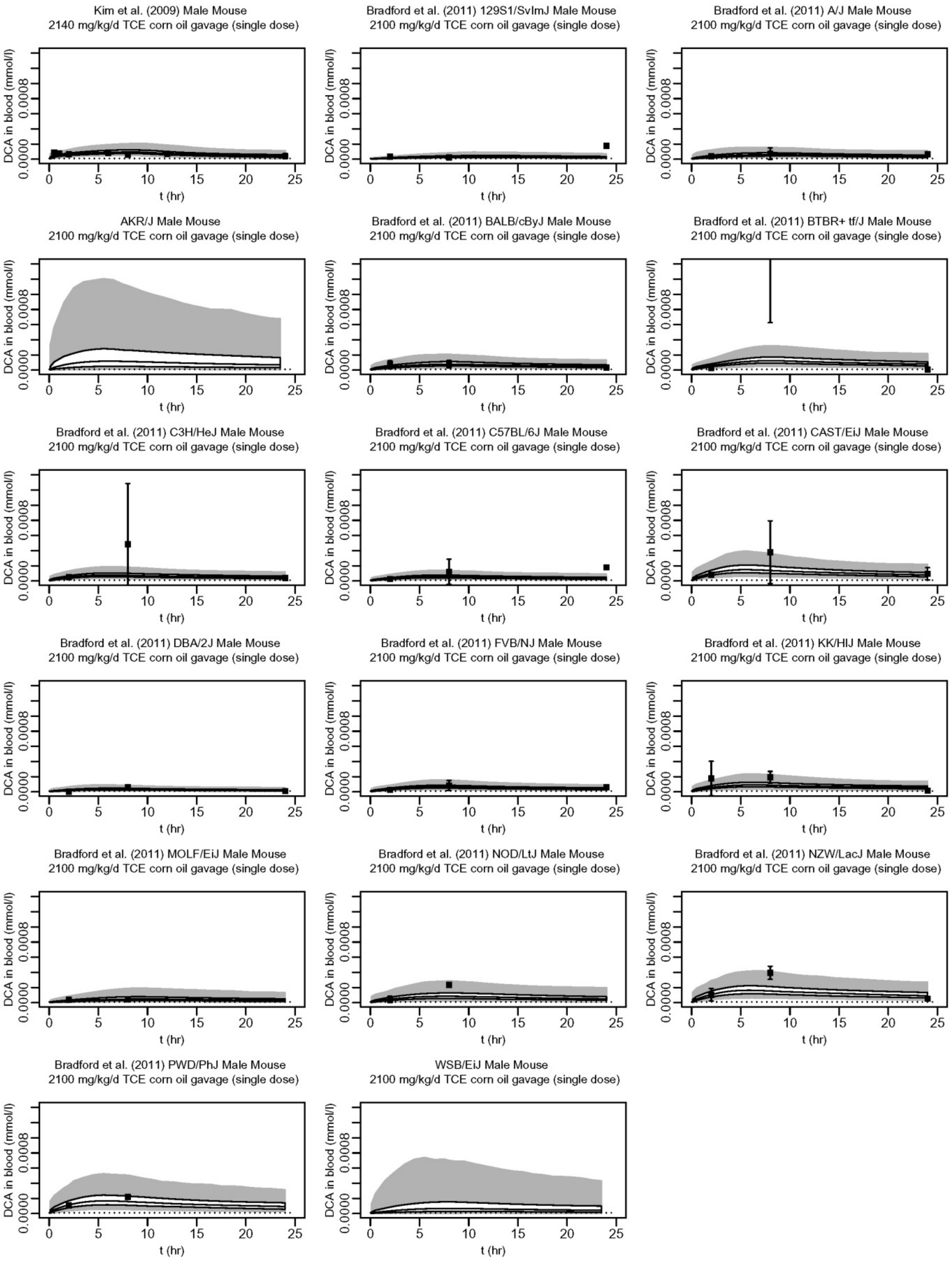
Kim et al. (2009) Male Mouse
2140 mg/kg/d TCE corn oil gavage (single dose)
Bradford et al. (2011) 129S1/SvImJ Male Mouse
2100 mg/kg/d TCE corn oil gavage (single dose)
Bradford et al. (2011) A/J Male Mouse
2100 mg/kg/d TCE corn oil gavage (single dose)
AKR/J Male Mouse
2100 mg/kg/d TCE corn oil gavage (single dose)
Bradford et al. (2011) BALB/cByJ Male Mouse
2100 mg/kg/d TCE corn oil gavage (single dose)
Bradford et al. (2011) BTBR+ tf/J Male Mouse
2100 mg/kg/d TCE corn oil gavage (single dose)
Bradford et al. (2011) C3H/HeJ Male Mouse
2100 mg/kg/d TCE corn oil gavage (single dose)
Bradford et al. (2011) C57BL/6J Male Mouse
2100 mg/kg/d TCE corn oil gavage (single dose)
Bradford et al. (2011) CAST/EiJ Male Mouse
2100 mg/kg/d TCE corn oil gavage (single dose)
Bradford et al. (2011) DBA/2J Male Mouse
2100 mg/kg/d TCE corn oil gavage (single dose)
Bradford et al. (2011) FVB/NJ Male Mouse
2100 mg/kg/d TCE corn oil gavage (single dose)
Bradford et al. (2011) KK/HIJ Male Mouse
2100 mg/kg/d TCE corn oil gavage (single dose)
Bradford et al. (2011) MOLF/EiJ Male Mouse
2100 mg/kg/d TCE corn oil gavage (single dose)
Bradford et al. (2011) NOD/LtJ Male Mouse
2100 mg/kg/d TCE corn oil gavage (single dose)
Bradford et al. (2011) NZW/LacJ Male Mouse
2100 mg/kg/d TCE corn oil gavage (single dose)
Bradford et al. (2011) PWD/PhJ Male Mouse
2100 mg/kg/d TCE corn oil gavage (single dose)
WSB/EiJ Male Mouse
2100 mg/kg/d TCE corn oil gavage (single dose)
DCA in blood (mmol/l)
0.0000
0.0008
t (hr)
0
5
10
15
20
25

Figure S6. Comparison of data (solid boxes with +/- 1 SD error bars) and PBPK model predictions (solid lines: interquartile range; grey area: 95% confidence interval) for DCVG in mouse inbred strains [data from (Bradford et al 2011)].

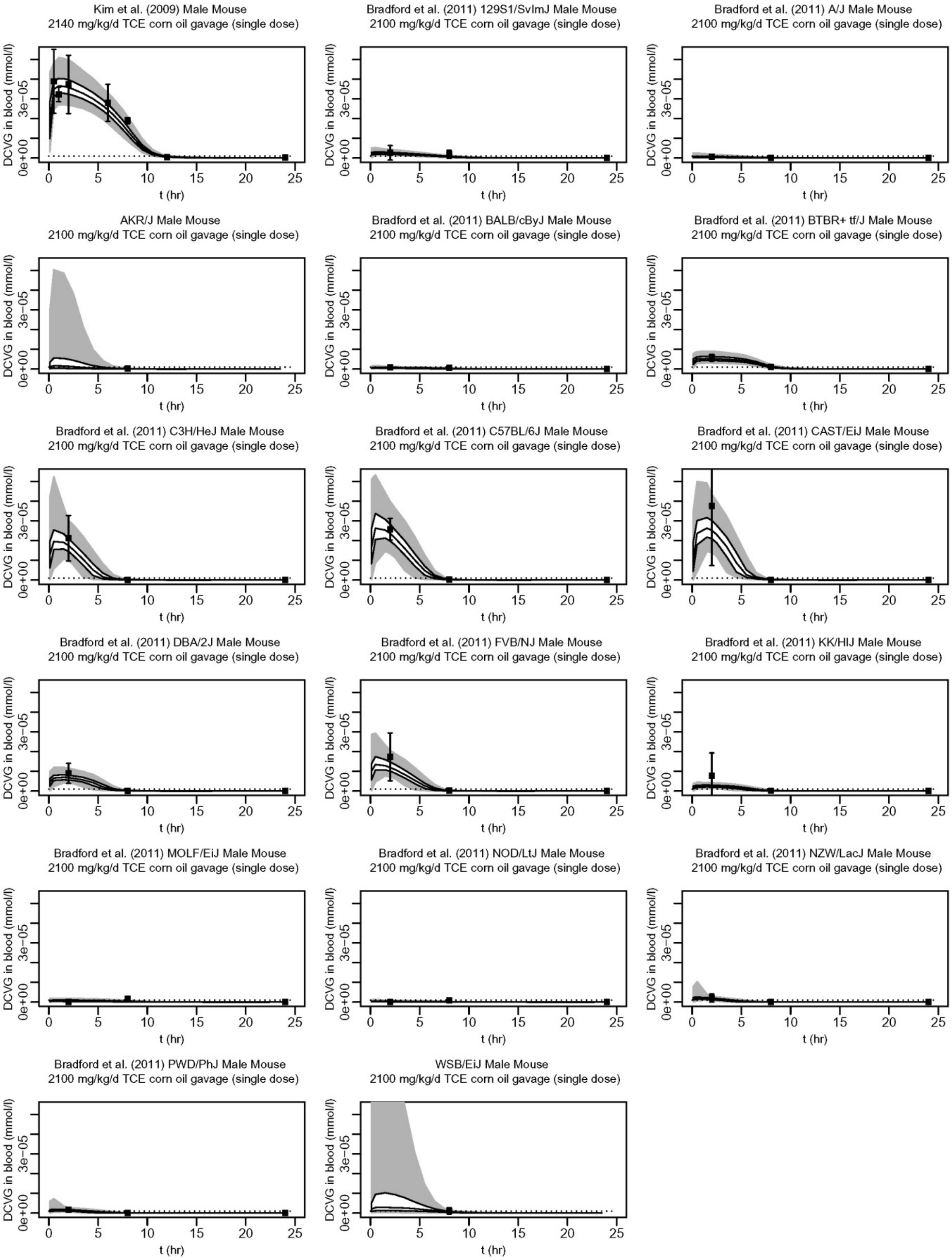
Kim et al. (2009) Male Mouse
2140 mg/kg/d TCE corn oil gavage (single dose)
Bradford et al. (2011) 129S1/SvImJ Male Mouse
2100 mg/kg/d TCE corn oil gavage (single dose)
Bradford et al. (2011) A/J Male Mouse
2100 mg/kg/d TCE corn oil gavage (single dose)
AKR/J Male Mouse
2100 mg/kg/d TCE corn oil gavage (single dose)
Bradford et al. (2011) BALB/cByJ Male Mouse
2100 mg/kg/d TCE corn oil gavage (single dose)
Bradford et al. (2011) BTBR+ tf/J Male Mouse
2100 mg/kg/d TCE corn oil gavage (single dose)
Bradford et al. (2011) C3H/HeJ Male Mouse
2100 mg/kg/d TCE corn oil gavage (single dose)
Bradford et al. (2011) C57BL/6J Male Mouse
2100 mg/kg/d TCE corn oil gavage (single dose)
Bradford et al. (2011) CAST/EiJ Male Mouse
2100 mg/kg/d TCE corn oil gavage (single dose)
Bradford et al. (2011) DBA/2J Male Mouse
2100 mg/kg/d TCE corn oil gavage (single dose)
Bradford et al. (2011) FVB/NJ Male Mouse
2100 mg/kg/d TCE corn oil gavage (single dose)
Bradford et al. (2011) KK/HIJ Male Mouse
2100 mg/kg/d TCE corn oil gavage (single dose)
Bradford et al. (2011) MOLF/EiJ Male Mouse
2100 mg/kg/d TCE corn oil gavage (single dose)
Bradford et al. (2011) NOD/LtJ Male Mouse
2100 mg/kg/d TCE corn oil gavage (single dose)
Bradford et al. (2011) NZW/LacJ Male Mouse
2100 mg/kg/d TCE corn oil gavage (single dose)
Bradford et al. (2011) PWD/PhJ Male Mouse
2100 mg/kg/d TCE corn oil gavage (single dose)
WSB/EiJ Male Mouse
2100 mg/kg/d TCE corn oil gavage (single dose)
DCVG in blood (mmol/l)
0e+00
3e−05
t (hr)
0
5
10
15
20
25

Figure S7. Comparison of data (solid boxes with ± 1 SD error bars) and PBPK model predictions (solid lines: interquartile range; grey area: 95% confidence interval) for DCVC in mouse inbred strains [data from (Bradford et al 2011)].

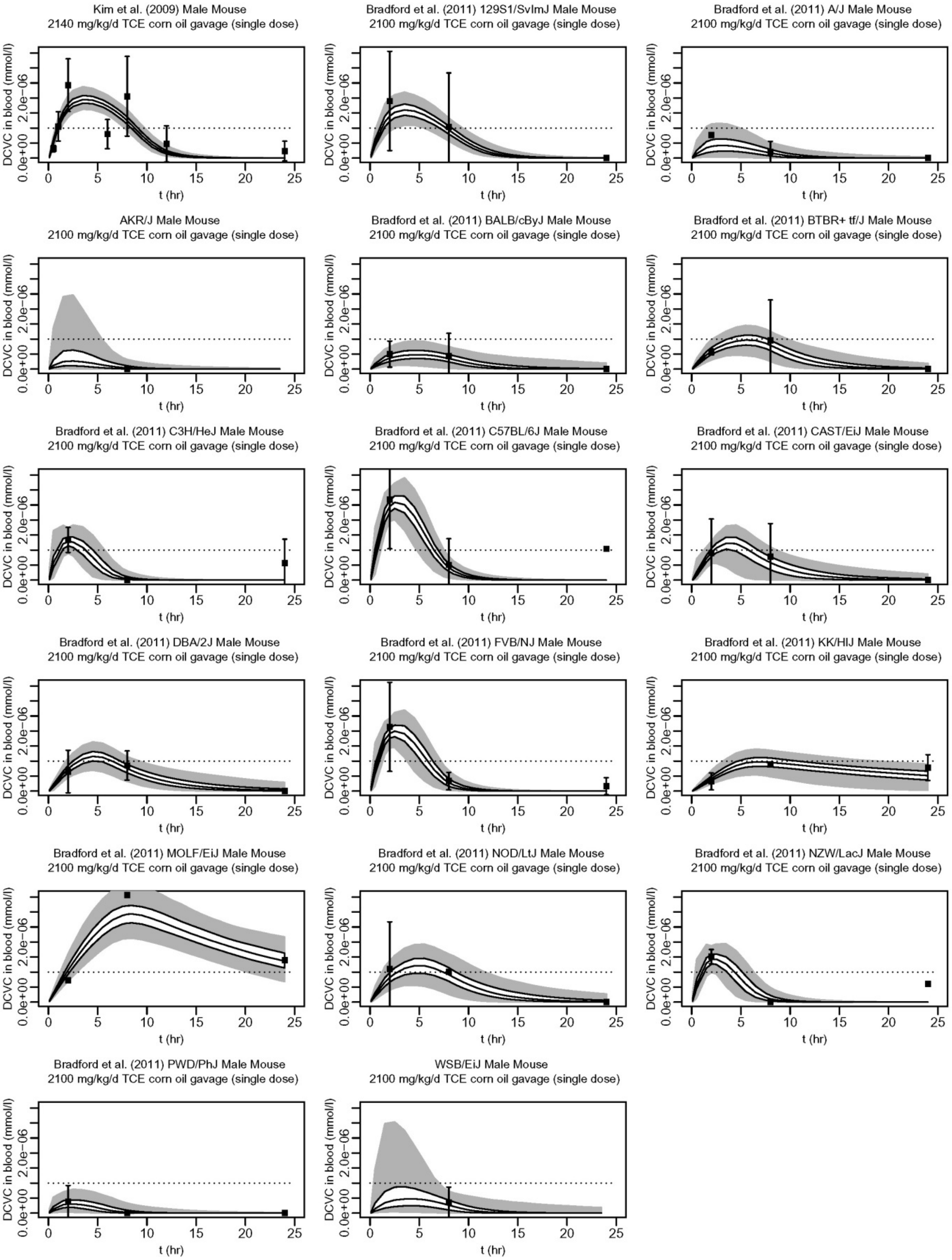
Kim et al. (2009) Male Mouse
2140 mg/kg/d TCE corn oil gavage (single dose)
Bradford et al. (2011) 129S1/SvImJ Male Mouse
2100 mg/kg/d TCE corn oil gavage (single dose)
Bradford et al. (2011) A/J Male Mouse
2100 mg/kg/d TCE corn oil gavage (single dose)
AKR/J Male Mouse
2100 mg/kg/d TCE corn oil gavage (single dose)
Bradford et al. (2011) BALB/cByJ Male Mouse
2100 mg/kg/d TCE corn oil gavage (single dose)
Bradford et al. (2011) BTBR+ tf/J Male Mouse
2100 mg/kg/d TCE corn oil gavage (single dose)
Bradford et al. (2011) C3H/HeJ Male Mouse
2100 mg/kg/d TCE corn oil gavage (single dose)
Bradford et al. (2011) C57BL/6J Male Mouse
2100 mg/kg/d TCE corn oil gavage (single dose)
Bradford et al. (2011) CAST/EiJ Male Mouse
2100 mg/kg/d TCE corn oil gavage (single dose)
Bradford et al. (2011) DBA/2J Male Mouse
2100 mg/kg/d TCE corn oil gavage (single dose)
Bradford et al. (2011) FVB/NJ Male Mouse
2100 mg/kg/d TCE corn oil gavage (single dose)
Bradford et al. (2011) KK/HIJ Male Mouse
2100 mg/kg/d TCE corn oil gavage (single dose)
Bradford et al. (2011) MOLF/EiJ Male Mouse
2100 mg/kg/d TCE corn oil gavage (single dose)
Bradford et al. (2011) NOD/LtJ Male Mouse
2100 mg/kg/d TCE corn oil gavage (single dose)
Bradford et al. (2011) NZW/LacJ Male Mouse
2100 mg/kg/d TCE corn oil gavage (single dose)
Bradford et al. (2011) PWD/PhJ Male Mouse
2100 mg/kg/d TCE corn oil gavage (single dose)
WSB/EiJ Male Mouse
2100 mg/kg/d TCE corn oil gavage (single dose)
DCVC in blood (mmol/l)
0.0e+00
2.0e−06
t (hr)
0 5 10 15 20 25

Table S1. TCE metabolite data for individual mice (rows) of AKR/J and WSB/EiJ strains.

Strain	Time point (hrs)	Treatment	DCVC (nmol/ml)	DCVG (nmol/ml)
AKR/J	8	TCE (2100 mg/kg)		5.76E-05
AKR/J	8	TCE (2100 mg/kg)		5.68E-04
AKR/J	8	Vehicle		1.77E-05
AKR/J	8	Vehicle		1.28E-04
WSB/EiJ	8	TCE (2100 mg/kg)	7.17E-04	0.00228

Table S2. Parameter distributions estimated with Monte Carlo analysis of the multistrain mouse data*.

Parameter	Mean	Standard Deviation	CV
FRACTCE	0.1	0.04	0.4
KURNTCAC	0.86	0.344	0.4
FRACDCA	0.000045	0	0.4
KTCADCAC	0.00201	0.001	0.4
KCLEARDCAC	3.688	1.475	0.4
KTCEDCVGC	0.0000195	0	0.5
KDCVGDCVCC	0.204	0.102	0.5
KCLEARDCVCC	0.394	0.197	0.5

Where FRACTCE is the fractional split of TCE metabolism to TCA, KURNTCAC is the rate constant for urinary clearance of TCA, FRACDCA is the fractional split of TCE metabolism to DCA, KTCADCAC is the rate constant for TCA metabolism to DCA, KCLEARDCAC is the rate constant for clearance of DCA, KTCEDCVGC is the rate constant for TCE metabolism to DCVG, KDCVGDCVCC is the rate constant for metabolism of DCVG to DCVC, and KCLEARDCVCC is the rate constant for clearance of DCVC.

*All parameters were distributed lognormally in the Monte Carlo simulation of the multistrain rat time-course data.

Table S3. PBPK model parameters, baseline values, and scaling relationships.

Model parameter: Symbol	Model parameter: Description (units)	Model parameter: Scaling formula	Scaling parameter: Symbol	Baseline value: Symbol	Baseline value: Description	Baseline value	Notes
BW	Body weight (kg)	–	–	–	–	–	a
QC	Cardiac output (L/hr)	QC = QCC0 × exp(lnQCC) × $BW^{3/4}$	lnQCC	QCC0	Cardiac output allometrically scaled	11.6	b
QP	Alveolar ventilation (L/hr)	QP = QC × VPR0 × exp(lnVPR)	lnVPRC	VPR0	Ventilation-perfusion ratio	2.5	c
DResp	Diffusion clearance rate (L/hr)	DResp = QP × exp(lnDRespC)	lnDRespC	–	–	–	d
QFat	Blood flow to fat (L/hr)	QFat = QC × QFatC0 × QFatC	QFatC	QFatC0	Fraction of blood flow to fat	0.07	e
QGut	Blood flow to gut (L/hr)	QGut = QC × QGutC0 × QGutC	QGutC	QGutC0	Fraction of blood flow to gut	0.141	e
QLiv	Hepatic artery blood flow (L/hr)	QLiv = QC × QLivC0 × QLivC	QLivC	QLivC0	Fraction of blood flow to hepatic artery	0.02	e
QSlw	Blood flow to slowly perfused tissues (L/hr)	QSlw = QC × QSlwC0 × QSlwC	QSlwC	QSlwC0	Fraction of blood flow to slowly perfused tissues	0.217	e
QKid	Blood flow to kidney (L/hr)	QKid = QC × QKidC0 × QKidC	QKidC	QKidC0	Fraction of blood flow to kidney	0.091	e
QRap	Blood flow to rapidly perfused tissues (L/hr)	QRap = QC–(QFat + QGut + QLiv + QSlw + QKid)	–	–	–	–	e
FracPlas	Fraction of blood that is plasma	FracPlas = FracPlas0 × FracPlasC	FracPlasC	FracPlas0	Fraction of blood that is plasma	0.52	f
VFat	Volume of fat (L)	VFat = BW × VFatC0 × VFatC	VFatC	VFatC0	Fraction of body weight that is fat	0.07	g

Model parameter: Symbol	Model parameter: Description (units)	Model parameter: Scaling formula	Scaling parameter: Symbol	Baseline value: Symbol	Baseline value: Description	Baseline value	Notes
VGut	Volume of gut (L)	VGut = BW × VGutC0 × VGutC	VGutC	VGutC0	Fraction of body weight that is gut	0.049	g
VLiv	Volume of liver (L)	VLiv = BW × VLivC0 × VLivC	VLivC	VLivC0	Fraction of body weight that is liver	0.055	g
VRap	Volume of rapidly perfused tissues (L)	VRap = BW × VRapC0 × VRapC	VRapC	VRapC0	Fraction of body weight that is rapidly perfused	0.1	g
VRespLum	Volume of respiratory tract lumen (L)	VRespLum = BW × VRespLumC0 × VRespLumC	VRespLumC	VRespLumC0	Respiratory lumen volume as fraction body weight	0.004667	g
VResp	Volume of respiratory tract tissue (L)	VResp = BW × VRespC0 × VRespC	VRespC	VRespC0	Fraction of body weight that is respiratory tract	0.0007	g
VRespEff	Effective air volume of respiratory tract tissue	VRespEff = VResp × PResp × PB	–	–	–	–	g
VKid	Volume of kidney (L)	VKid = BW × VKidC0 × VKidC	VKidC	VKidC0	Fraction of body weight that is kidney	0.017	g
VBld	Volume of blood (L)	VBld = BW × VBldC0 × VBldC	VBldC	VBldC0	Fraction of body weight that is blood	0.049	g
VSlw	Volume of slowly perfused tissue (L)	VSlw = BW × VperfC0 – (VFat + VGut + VLiv + VRap + VResp + VKid + VBld)	–	VperfC0	Fraction of body weight that is blood perfused	0.8897	g
VPlas	Volume of plasma (L)	VPlas = FracPlas × VBld	–	–	–	–	h
VBod	Volume body for TCA submodel (L)	VBod = VFat + VGut + VRap + VResp + VKid + VSlw	–	–	–	–	i

Model parameter: Symbol	Model parameter: Description (units)	Model parameter: Scaling formula	Scaling parameter: Symbol	Baseline value: Symbol	Baseline value: Description	Baseline value	Notes
VBodTCOH	Volume body for TCOH and TCOG submodels (L)	VBodTCOH = VBod + VBld	–	–	–	–	j
PB	TCE blood-air partition coefficient	PB=PB0×PBC	PBC	PB0	TCE blood-air partition coefficient	15	k
PFat	TCE fat-blood partition coefficient	PFat=PFatC0×exp(PFatC)	PFatC	PFatC0	TCE fat-blood partition coefficient	36	l
PGut	TCE gut-blood partition coefficient	PGut=(PGutC0)×exp(lnPGutC)	lnPGutC	PGutC0	TCE gut-blood partition coefficient	1.9	m
PLiv	TCE liver-blood partition coefficient	PLiv = (PLivC0) × exp(lnPLivC)	lnPLivC	PLivC0	TCE liver-blood partition coefficient	1.7	n
PRap	TCE rapidly perfused-blood partition coefficient	PRap = (PRapC0) × exp(lnPRapC)	lnPRapC	PRapC0	TCE rapidly perfused-blood partition coefficient	1.9	o
PResp	TCE respiratory tract tissue-blood partition coefficient	Presp = (PRespC0) × exp(lnPRespC)	lnPRespC	PRespC0	TCE respiratory tract tissue-blood partition coefficient	2.6	p
PKid	TCE kidney-blood partition coefficient	PKid = (PKidC0) × exp(lnPKidC)	lnPKidC	PKidC0	TCE kidney-blood partition coefficient	2.1	q
PSlw	TCE slowly perfused-blood partition coefficient	PSlw = (PSlwC0) × exp(lnPSlwC)	lnPSlwC	PSlwC0	TCE slowly perfused-blood partition coefficient	2.4	r
TCAPlas	TCA blood-plasma concentration ratio	TCAPlas = FracPlas + (1 – FracPlas) × PRBCPlasTCA0 × exp(lnPRBCPlasTCAC)	lnPRBCPlasTCAC	PRBCPlasTCA0	TCA red blood cell-plasma partition coefficient	0.5	s

Model parameter: Symbol	Model parameter: Description (units)	Model parameter: Scaling formula	Scaling parameter: Symbol	Baseline value: Symbol	Baseline value: Description	Baseline value	Notes
PBodTCA	Free TCA body-plasma partition coefficient	PBodTCA = TCAPlas × PBodTCAC0 × exp(lnPBodTCAC)	lnPBodTCAC	PBodTCAC0	Free TCA body-blood partition coefficient	0.88	t
PLivTCA	Free TCA liver-plasma partition coefficient	PLivTCA = TCAPlas × PLivTCAC0 × exp(lnPLivTCAC)	lnPLivTCAC	PLivTCAC0	Free TCA liver-blood partition coefficient	1.18	t
kDissoc	Protein TCA dissociation constant (microM)	kDissoc = kDissoc0 × exp(lnkDissocC)	lnkDissocC	kDissoc0	Protein TCA dissociation constant (microM)	107	u
BMax	Protein concentration (microM)	BMax = BMaxkD0 × kDissoc × exp(lnBMaxkDC)	lnBMaxkDC	BMaxkD0	BMax/kDissoc ratio	0.88	u
PBodTCOH	TCOH body-blood partition coefficient	PBodTCOH = PBodTCOH0 × exp(lnPBodTCOHC)	lnPBodTCOHC	PBodTCOH0	TCOH body-blood partition coefficient	1.11	v
PLivTCOH	TCOH liver-blood partition coefficient	PBodTCOH = PLivTCOH0 × exp(lnPLivTCOHC)	lnPLivTCOHC	PLivTCOH0	TCOH liver-blood partition coefficient	1.3	v
PBodTCOG	TCOG body-blood partition coefficient	PBodTCOG = PBodTCOG0 × exp(lnPBodTCOGC)	lnPBodTCOGC	PBodTCOG0	TCOG body-blood partition coefficient	1.11	w
PLivTCOG	TCOG liver-blood partition coefficient	PBodTCOG = PLivTCOG0 × exp(lnPLivTCOGC)	lnPLivTCOGC	PLivTCOG0	TCOG liver-blood partition coefficient	1.3	w
VDCVG	DCVG distribution volume (L)	VDCVG = VBld + (VBod+VLiv) × exp(lnPeffDCVG)	lnPeffDCVG	–	–	–	x
VDCVC	DCVC distribution volume (L)	VDCVC = VBld + (VBod+VLiv) × exp(lnPeffDCVC)	lnPeffDCVC	–	–	–	x

Model parameter: Symbol	Model parameter: Description (units)	Model parameter: Scaling formula	Scaling parameter: Symbol	Baseline value: Symbol	Baseline value: Description	Baseline value	Notes
VDCA	DCA distribution volume (L)	VDCA = VBld + (VBod+VLiv) × exp(lnPeffDCA)	lnPeffDCA	–	–	–	x
VMAX	VMAX for TCE hepatic oxidation (mg/hr)	VMAX = VMAX0 × VLiv × exp(lnVMAXC)	lnVMAXC	VMAX0	VMAX per kg liver for TCE hepatic oxidation (mg/hr/kg liver)	2,700	y
KM	KM for TCE hepatic oxidation (mg/L blood)	KM = KM0 × exp(lnKMC)	lnKMC	KM0	KM for TCE hepatic oxidation (mg/L)	36	y
FracOther	Fraction of TCE oxidation not to TCA or TCOH	FracOther = exp(lnFracOtherC)/ (1+exp(lnFracOtherC))	lnFracOtherC	–	–	–	z
FracTCA	Fraction of TCE oxidation to TCA	FracTCA = (1-FracOther) × logitFracTCA0 × exp(lnFracTCAC)/ (1 + logitFracTCA0 × exp(lnFracTCAC))	lnFracTCAC	logitFracTCA0	Log of ratio of fraction to TCA to fraction not to TCA	0.32	aa
VMAXDCVG	VMAX for TCE hepatic GSH conjugation (mg/hr)	VMAXDCVG = VMAXDCVG0 × VLiv × exp(lnVMAXDCVGC)	lnVMAXDCVGC	VMAXDCVG0	VMAX per kg liver for TCE GSH conjugation (mg/hr/kg liver)	300	bb
KMDCVG	KM for TCE hepatic GSH conjugation (mg/L blood)	KMDCVG = VMAXDCVG/ (ClDCVG0 × exp(lnClDCVGC)	lnClDCVGC	ClDCVG0	VMAX/KM per kg liver for TCE hepatic GSH conjugation (L blood/hr/kg liver)	1.53	bb
KMClara	KM for TCE lung oxidation (mg/L air)	KMClara = exp(lnKMClara)	–	–	–	–	cc

Model parameter: Symbol	Model parameter: Description (units)	Model parameter: Scaling formula	Scaling parameter: Symbol	Baseline value: Symbol	Baseline value: Description	Baseline value	Notes
VMAXClara	VMAX for TCE lung oxidation (mg/hr)	VMAXClara = VMAX × VMAXLungLiv0 × exp(lnVMAXLungLivC)	lnVMAXLungLivC	VMAXLungLiv0	Ratio of lung to liver total VMAX (mg/hr per mg/hr)	0.07	cc
FracLungSys	Fraction of respiratory oxidation entering systemic circulation	FracLungSys = exp(lnFracLungSysC)/(1 + exp(lnFracLungSysC))	lnFracLungSysC	–	–	–	dd
VMAXTCOH	VMAX for TCOH oxidation to TCA (mg/hr)	VMAXTCOH= $BW^{3/4}$ × exp(lnVMAXTCOHC)	lnVMAXTCOHC	–	–	–	
KMTCOH	KM for TCOH oxidation to TCA (mg/L air)	KMTCOH = exp(lnKMTCOHC)	lnKMTCOHC	–	–	–	
VMAXGluc	VMAX for TCOH glucuronidation (mg/hr)	VMAXGluc = $BW^{3/4}$ × exp(lnVMAXGlucC)	lnVMAXGlucC	–	–	–	
KMGluc	KM for TCOH glucuronidation (mg/L air)	KMGluc = exp(lnKMGlucC)	lnKMGlucC	–	–	–	
kMetTCOH	Rate constant for TCOH other clearance (/hr)	kMetTCOH = $BW^{-1/4}$ × exp(lnkMetTCOHC)	lnkMetTCOHC	–	–	–	
kUrnTCA	Rate constant for TCA excretion to urine (/hr)	kUrnTCA = GFR_BW × exp(lnkUrnTCAC) × BW /VPlas	lnkUrnTCAC	GFR_BW	Glomerular filtration rate per kg body weight (L/h/kg)	0.6	ee
kMetTCA	Rate constant for other TCA clearance (/hr)	kMetTCA = $BW^{-1/4}$ × exp(lnkMetTCAC)	lnkMetTCAC	–	–	–	
kBile	Rate constant for other TCOG excretion to bile (/hr)	kBile = $BW^{-1/4}$ × exp(lnkBileC)	lnkBileC	–	–	–	

Model parameter: Symbol	Model parameter: Description (units)	Model parameter: Scaling formula	Scaling parameter: Symbol	Baseline value: Symbol	Baseline value: Description	Baseline value	Notes
kEHR	Rate constant for other bile TCOG reaborption as TCOH (/hr)	kEHR = $BW^{-1/4}$ × exp(lnkEHRC)	lnkEHRC	–	–	–	
kUrnTCOG	Rate constant for TCOH excretion to urine (/hr)	kUrnTCOG = GFR_BW × exp(lnkUrnTCOGC) × BW/(VBodTCOH × PBodTCOG)	lnkUrnTCOGC	GFR_BW	Glomerular filtration rate per kg body weight (L/hr/kg)	0.6	ee
kDCVG	Rate constant for DCVC formation from DCVG (/hr)	kDCVG = $BW^{-1/4}$ × exp(lnkDCVGC)	lnkDCVGC	–	–	–	
kElimDCVC	Lumped rate constant for elimination of DCVC (/hr)	kElimDCVC = $BW^{-1/4}$ × exp(lnkElimDCVCC)	lnkElimDCVCC	–	–	–	
kClearDCVC	Rate constant for clearance of DCA (/hr)	kClearDCA = $BW^{-1/4}$ × exp(lnkClearDCAC)	lnkClearDCAC	–	–	–	
kDCAcen_per	Rate constant for DCA central-to-peripheral (/hr)	kDCAcen_per = $BW^{-1/4}$ × exp(lnkDCAcen_perC)	lnkDCAcen_perC	–	–	–	
kDCAper_cen	Rate constant for DCA peripheral-to-central (/hr)	kDCAper_cen = $BW^{-1/4}$ × exp(lnkDCAper_cenC)	lnkDCAper_cenC	–	–	–	
kTSD	TCE gavage stomach-duodenum transfer coefficient (/hr)	kTSD = exp(lnkTSD)	lnkTSD	–	–	–	ff
kAS	TCE gavage stomach-absorption coefficient (/hr)	kAS = exp(lnkAS)	lnkAS	–	–	–	ff
kAD	TCE gavage duodenum-absorption coefficient (/hr)	kAD = exp(lnkAD)	lnkAD	–	–	–	ff

Model parameter: Symbol	Model parameter: Description (units)	Model parameter: Scaling formula	Scaling parameter: Symbol	Baseline value: Symbol	Baseline value: Description	Baseline value	Notes
kASTCA	TCA stomach absorption coefficient (/hr)	kASTCA = exp(lnkASTCA)	lnkASTCA	–	–	–	ff
kASTCOH	TCOH stomach absorption coefficient (/hr)	kASTCOH = exp(lnkASTCOH)	lnkASTCOH	–	–	–	ff

Explanatory note: "Scaling parameters" are the parameters which may be given an uncertainty distribution in the statistical analysis. See Table S1 for the prior distributions for these parameters. For fixed parameters, see the notes below.

Notes: [a]Use measured value if available. Otherwise assume BW = 0.03 kg. [b]Baseline values are from Brown et al. (1997). [c]Scale by QC using alveolar VPR. Baseline values are from Brown et al. (1997). [d]Scaling parameter is relative to alveolar ventilation rate. [e]Fat represents adipose tissue only. Gut is the GI tract, pancreas, and spleen (all drain to the portal vein). Slowly perfused tissue is the muscle and skin. Rapidly perfused tissue is the rest of the organs, plus the bone marrow and lymph nodes, the blood flow for which is calculated as the difference between the cardiac output (QC) and the sum of the other blood flows. Baseline values are from Brown et al. (1997). Scaling parameter fixed at 1. [f]This is equal to 1 minus the hematocrit (measured value used if available). Baseline values from control animals in (Hejtmancik et al. 2002). Scaling parameter fixed at 1. [g]Fat represents adipose tissue only, and the measured value is used, if available. Gut is the GI tract, pancreas, and spleen (all drain to the portal vein). Rapidly perfused tissue is the rest of the organs, plus the bone marrow and lymph nodes, minus the tracheobronchial region. The respiratory tissue volume is tracheobronchial region, with an effective air volume given by multiplying by its tissue:air partition coefficient (= tissue:blood times blood:air). The slowly perfused tissue is the muscle and skin. This leaves a small (10–15% of body weight) unperfused volume that consists mostly of bone (minus marrow) and the GI tract contents. Baseline values are from Brown et al. (1997), except for volumes of the respiratory lumen, which are from Sarangapani et al. (2003). Scaling parameter fixed at 1. [h]Derived from blood volume using FracPlas. [i]Sum of all compartments except the blood and liver. [j]Sum of all compartments except the liver. [k]Pooling Abbas and Fisher (1997) and Fisher et al. (1991). Scaling parameter fixed at 1. [l]Abbas and Fisher (1997). Scaling parameter fixed at 1. [m]Value is the geometric mean of liver and kidney (relatively high uncertainty) values. Scaling parameter fixed at 0. [n]Fisher et al. (1991). Scaling parameter fixed at 0. [o]Geometric mean of liver and kidney values. Scaling parameter fixed at 0. [p]Lung value from Abbas and Fisher (1997). Scaling parameter fixed at 0. [q] Abbas and Fisher (1997). Scaling parameter fixed at 0. [r]Muscle value from Abbas and Fisher (1997). Scaling parameter fixed at 0. [s]Scaling parameter is the effective partition coefficient between red blood cells and plasma. Thus, the TCA blood-plasma concentration ratio depends on the plasma fraction. Baseline value is based on the blood-plasma concentration ratio of 0.76 in rats (Schultz et al. 1999). [t]In vitro partition coefficients were determined at high concentration, when plasma binding is saturated, so should reflect the free blood:tissue partition coefficient. To get the plasma partition coefficient, the partition coefficient is multiplied by the blood:plasma concentration ratio (TCAPlas). In vitro values were

from Abbas and Fisher (1997). Body values based on measurements in muscle. Scaling parameter fixed at 0. [u]Values are based on the geometric mean of estimates based on data from Lumpkin et al. (2003), Schultz et al. (1999), Templin et al. (1993; 1995), and Yu et al. (2000). Scaling parameter for BMAX is actually the ratio of BMAX/kD, which determines the binding at low concentrations. [v]Data are from Abbas and Fisher (1997). Scaling parameter fixed at 0. [w]Used in vitro measurements in TCOH as a proxy, but higher uncertainty is noted. Scaling parameter fixed at 0. [x]The scaling parameter is the effective partition coefficient for the "body" (nonblood) compartment, so that the distribution volume for X (V_X) is given by VBld + exp(lnPeff_X) × (VBod + VLiv). [y]Baseline values have the following units: for VMAX, mg/hr/kg liver; for KM, mg/L blood; and for clearance (Cl), L/hr/kg liver. Values are based on in vitro (microsomal and hepatocellular preparations) from Elfarra et al. (1998), Lipscomb et al. (1998a; 1997, 1998b). Scaling from in vitro data based on 32 mg microsomal protein/g liver and 99 × 106 hepatocytes/g liver (Barter et al. 2007). Scaling of KM from microsomes were based on two methods: (1) assuming microsomal concentrations equal to liver tissue concentrations and (2) using the measured microsome:air partition coefficient and a central estimate of the blood:air partition coefficient. [z]Scaling parameter is ratio of "DCA" to "non-DCA" oxidative pathway (where DCA is a proxy for oxidative metabolism not producing TCA or TCOH). Fraction of "other" oxidation is exp(lnFracOtherC)/(1 + exp[lnFracOtherC]). [aa]Scaling parameter is ratio of TCA to TCOH pathways. Baseline value based on geometric mean of Lipscomb et al. (1998a) using fresh hepatocytes and Bronley-DeLancey et al. (2006) using cryogenically-preserved hepatocytes. Fraction of oxidation to TCA is (1 – FracOther) × exp(lnFracTCAC)/(1 + exp[lnFracTCAC]). [bb]Baseline values are based on in vitro data at 1 or 2 mM (Lash et al. 1998; Lash et al. 1995). In most cases, rates at 2 mM were increased over the same sex/species at 1 mM, indicating VMAX has not yet been reached. These data therefore put lower bounds on both VMAX (in units of mg/hr/kg tissue) and clearance (in units of L/hr/kg tissue), so those are the scaling parameters used, with those bounds used as baseline values. [cc]Scaling parameter is the ratio of the lung to liver VMAX (each in units of mg/hr), with baseline values based on microsomal preparations (mg/hr/mg protein) assayed at ~1 mM (Green et al. 1997), further adjusted by the ratio of lung to liver tissue masses (Brown et al. 1997; Publication 89, ICRP 2003). [dd]Scaling parameter is the ratio of respiratory oxidation entering systemic circulation (translocated to the liver) to that locally cleared in the lung. Fraction of respiratory oxidation entering systemic circulation is exp(lnFracLungSysC)/(1 + exp[lnFracLungSysC]). [ee]Baseline parameters for urinary clearance (L/hr) were based on glomular filtration rate per unit body weight (L/hr/kg body weight) from Lin (1995), multiplied by the body weights cited in the study. For TCA, these were scaled by plasma volume to obtain the rate constant (/hr), since the model clears TCA from plasma. For TCOG, these were scaled by the effective distribution volume of the body (VBodTCOH × PBodTCOG) to obtain the rate constant (/hr), since the model clears TCOG from the body compartment. [ff]Baseline value for oral absorption scaling parameter are as follows: kTSD and kAS, 1.4/hr, based on human stomach half time of 0.5 hr; kAD, kASTCA, and kASTCOH, 0.75/hr, based on human small intestine transit time of 4 hrs (Publication 89, ICRP 2003). These are noted to have very high uncertainty.

Table S4. Uncertainty distributions for the population mean and variance of the PBPK model parameters.

Scaling parameter	Population mean: Distribution	Population mean: SD or Min	Population mean: Truncation (± nxSD) or Max	Population mean: Notes	Population variance: CV	Population variance: CU	Population variance: Notes
lnQCC	TruncNormal	0.2	4	a	0.2	2	aa
lnVPRC	TruncNormal	0.2	4	a	0.2	2	aa
lnDRespC	Uniform	-11.513	2.303	b	0.2	0.5	aa
lnPRBCPlasTCAC	Uniform	-4.605	4.605	c	0.336	2	bb
lnkDissocC	TruncNormal	1.191	3	d	1.191	2	cc
lnBMaxkDC	TruncNormal	0.495	3	d	0.495	2	cc
lnPeffDCVG	Uniform	-6.908	6.908	e	0.4	2	cc
lnPeffDCVC	Uniform	-6.908	6.908	e	0.4	2	cc
lnPeffDCA	Uniform	-6.908	6.908	e	0.4	2	cc
lnVMAXC	TruncNormal	0.693	3	f	0.824	1	dd
lnKMC	TruncNormal	1.386	3	f	0.270	1	dd
lnFracOtherC	Uniform	-6.908	6.908	e	0.5	2	ee
lnFracTCAC	TruncNormal	1.163	3	g	0.5	2	ee
lnVMAXDCVGC	Uniform	-6.908	6.908	h	0.5	2	ee
lnClDCVGC	Uniform	-6.908	6.908	h	0.5	2	ee

Scaling parameter	Population mean: Distribution	Population mean: SD or Min	Population mean: Truncation (± nxSD) or Max	Population mean: Notes	Population variance: CV	Population variance: CU	Population variance: Notes
lnVMAXLungLivC	TruncNormal	1.099	3	i	0.5	2	ee
lnKMClara	Uniform	-6.908	6.908	e	0.5	2	ee
lnFracLungSysC	Uniform	-6.908	6.908	e	0.5	2	ee
lnVMAXTCOHC	Uniform	-9.21	9.21	e	0.5	2	ee
lnKMTCOH	Uniform	-9.21	9.21	e	0.5	2	ee
lnVMAXGlucC	Uniform	-9.21	9.21	e	0.5	2	ee
lnKMGluc	Uniform	-6.908	6.908	e	0.5	2	ee
lnkMetTCOHC	Uniform	-11.513	6.908	e	0.5	2	ee
lnkUrnTCAC	Uniform	-4.605	4.605	e	0.5	2	ee
lnkMetTCAC	Uniform	-9.21	4.605	e	0.5	2	ee
lnkBileC	Uniform	-9.21	4.605	e	0.5	2	ee
lnkEHRC	Uniform	-9.21	4.605	e	0.5	2	ee
lnkUrnTCOGC	Uniform	-6.908	6.908	e	0.5	2	ee
lnkDCVGC	Uniform	-9.21	4.605	e	0.5	2	ee
lnkElimDCVCC	Uniform	-9.21	4.605	e	0.5	2	ee
lnkClearDCAC	Uniform	-9.21	4.605	e	0.5	2	ee

Scaling parameter	Population mean: Distribution	Population mean: SD or Min	Population mean: Truncation (± nxSD) or Max	Population mean: Notes	Population variance: CV	Population variance: CU	Population variance: Notes
lnkDCAcen_perC	Uniform	-9.21	4.605	e	0.5	2	ee
lnkDCAper_cenC	Uniform	-9.21	4.605	e	0.5	2	ee
lnkTSD	Uniform	-4.269	4.942	e	2	2	ff
lnkAS	Uniform	-6.571	7.244	e	2	2	ff
lnkAD	Uniform	-7.195	6.62	e	2	2	ff
lnkASTCA	Uniform	-7.195	6.62	e	2	2	ff
lnkASTCOH	Uniform	-7.195	6.62	e	2	2	ff

Explanatory note: All population mean parameters have either truncated normal (TruncNormal) or uniform distributions. For those with TruncNormal distributions, the mean for the population mean is 0 for natural-log transformed parameters (parameter name starting with "ln") and one for untransformed parameters, with the truncation at the specified number (n) of SDs. All uniformly distributed parameters are natural-log transformed, so their untransformed minimum and maximum are exp(Min) and exp(Max), respectively. All population variance parameters (V_pname, for parameter "pname") have Inverse-Gamma distributions, with the expected value given by CV and coefficient of uncertainty given by CU (i.e., SD of V_pname divided by expected value of V_pname) (notation the same as Hack et al. 2006). Under these conditions, the Inverse-Gamma distribution has a shape parameter is given by $\alpha = 2 + 1/CU^2$ and scale parameter $\beta = (\alpha - 1)\ CV^2$. In addition, it should be noted that, under a normal distribution and a uniform prior distribution on the population variance, the posterior distribution for the variance given n data points with a sample variance s^2 is given by and Inverse-Gamma distribution with $\alpha = (n - 1)/2$ and $\beta = \alpha\ s^2$. Therefore, the "effective" number of data points is given by $n = 5 + 2/CU^2$ and the "effective" sample variance is $s^2 = CV^2\ \alpha/(\alpha - 1)$.

Notes: [a]Uncertainty based on coefficient of variation (CV) or range of values in Brown et al. (1997). [b]Noninformative prior distribution intended to span a wide range of possibilities because no independent data are available on these parameters. [c]No in vitro data was available, so a noninformative prior was used. [d]GSD for uncertainty based on different estimates from different in vitro studies. [e]Noninformative prior

distribution. [f]Assume twofold uncertainty GSD in VMAX, based on observed variability and uncertainties of in vitro-to-in vivo scaling. For KM, the uncertainty is assumed to be fourfold, due to the different methods for scaling of concentrations from TCE in the in vitro medium to TCE in blood. [g]Uncertainty GSD of 3.2-fold reflects difference between in vitro measurements from Lipscomb et al. (1998a) and Bronley-DeLancey et al. (2006). [h]The baseline values are notional lower-limits on VMAX and clearance; however, the lower bound of the prior distribution is set to 100-fold less because of uncertainty in in vitro-in vivo extrapolation, and because Green et al. (1997) reported values 100-fold smaller than Lash et al. (1998; 1995). [i]Uncertainty GSD of threefold was assumed due to possible differences in microsomal protein content, the fact that measurements were at a single concentration, and the fact that the human baseline values was based on the limit of detection. [aa]CV values generally taken to be equal to the uncertainty SD in the population mean, most of which were based on variability between studies (i.e., not clear whether variability represents uncertainty or variability). Given this uncertainty, CU of 2 assigned to cardiac output and ventilation-perfusion, while CU of 0.5 assigned to the remaining physiological parameters. [bb]Used value from uncertainty in population in mean in rats for all species with high uncertainty. [cc]It is not clear whether interstudy variability is due to intersubject or assay variability, so the same central were assigned to the uncertainty in the population mean as to the central estimate of the population variance. In the cases where direct measurements were available, the CU for the uncertainty in the population variance is based on the actual sample n, with the derivation discussed in the notes preceding this table. Otherwise, a CU of 2 was assigned, reflecting high uncertainty. [dd]Based on variability in results from Lipscomb et al. (1998b) and Elfarra et al. (1998) in microsomes. Since only pooled or mean values are available, CU of one was assigned (moderate uncertainty). [ee]No data on variability, so a CV of 0.5 was assigned, with a CU of 2. [ff]No data on variability, so a CV of 2 was assigned (larger than assumed for metabolism due to possible vehicle effects), with a CU of 2.

Table S5. Interstrain variability parameters.

Interstrain scaling parameter	Parameter scaling	Population mean: Distribution	Population mean: SD or Min	Population mean: Truncation (± nxSD) or Max	Population variance: CV	Population variance: CU
lnISOx	VMAX → VMAX × exp(lnISOx)	Uniform	-4.605	4.605	2	2
lnISTCA	lnFracTCAC → lnFracTCAC + lnISTCA	Uniform	-4.605	4.605	2	2
lnISDCA	FracOther → FracOther × exp(lnISDCA)	Uniform	-4.605	4.605	2	2
lnISConj	VMAXDCVG → VMAXDCVG × exp(lnISConj)	Uniform	-4.605	4.605	2	2
lnISkTCA	kUrnTCA → kUrnTCA × exp(lnISTCA)<br>kMetTCA → kMetTCA × exp(lnISTCA)	Uniform	-4.605	4.605	2	2
lnISkDCVG	kDCVG → kDCVG× exp(lnISDCVG)	Uniform	-4.605	4.605	2	2
lnISkDCVC	kElimDCVC → kElimDCVC× exp(lnISDCVG)	Uniform	-4.605	4.605	2	2

Table S6. Comparison of prior and posterior distributions for scaling parameters.

Parameter	Population Mean: Prior Median	Population Mean: Prior 2.5%	Population Mean: Prior 97.5%	Population Mean: Posterior Median	Population Mean: Posterior 2.5%	Population Mean: Posterior 97.5%	Population Variability: Prior CV	Population Variability: Prior CU	Population Variability: Posterior CV	Population Variability: Posterior CU
lnQCC	0	-0.392	0.392	0.152	-0.0798	0.358	0.2	2	0.206	0.214
lnVPRC	0	-0.392	0.392	-0.319	-0.574	-0.0191	0.2	2	0.27	0.301
lnDRespC	-4.61	-11.2	1.96	0.83	0.442	1.3	0.2	0.5	0.212	0.117
lnPRBCPlasTCAC	0	-4.37	4.37	0.889	-0.212	1.74	0.336	2	0.423	0.59
lnkDissocC	0	-2.33	2.33	0.467	-0.713	1.78	1.19	2	1.15	1.18
lnBMaxkDC	0	-0.97	0.97	0.421	-0.0813	0.929	0.495	2	0.45	0.534
lnPeffDCVG	0	-6.56	6.56	0.0903	-5.22	2.46	0.4	2	0.406	0.456
lnPeffDCVC	0	-6.56	6.56	5.98	4.61	6.85	0.4	2	0.408	0.435
lnPeffDCA	0	-6.56	6.56	3.67	-0.355	6.35	0.4	2	0.424	0.473
lnVMaxC	0	-1.36	1.36	-0.633	-1.23	0.00521	0.824	1	0.74	0.568
lnKMC	0	-2.72	2.72	-2.44	-3.41	-1.43	0.27	1	1.01	0.869
lnFracOtherC	0	-6.56	6.56	-4.16	-6.16	-1.95	0.5	2	0.537	0.621
lnFracTCAC	0	-2.28	2.28	-1.43	-2.08	-0.761	0.5	2	0.477	0.573
lnVMaxDCVGC	0	-6.56	6.56	-4.96	-6.46	-3.29	0.5	2	0.525	0.728
lnClDCVGC	0	-6.56	6.56	-3.63	-5.26	1.09	0.5	2	0.551	1.29
lnVMaxLungLivC	0	-2.15	2.15	2.28	0.525	3.11	0.5	2	0.81	1.33
lnKMClara	0	-6.56	6.56	-5.05	-6.56	-3.33	0.5	2	0.571	0.808
lnFracLungSysC	0	-6.56	6.56	2.72	-0.0928	5.41	0.5	2	0.615	1.23
lnVMaxTCOHC	0	-8.75	8.75	1.14	0.477	1.89	0.5	2	0.655	0.455
lnKMTCOH	0	-8.75	8.75	-0.0643	-1.29	0.952	0.5	2	0.554	0.713
lnVMaxGlucC	0	-8.75	8.75	4.63	3.76	6.32	0.5	2	0.501	0.508
lnKMGluc	0	-6.56	6.56	3.41	2.03	5.21	0.5	2	0.606	0.95

Parameter	Population Mean: Prior Median	Population Mean: Prior 2.5%	Population Mean: Prior 97.5%	Population Mean: Posterior Median	Population Mean: Posterior 2.5%	Population Mean: Posterior 97.5%	Population Variability: Prior CV	Population Variability: Prior CU	Population Variability: Posterior CV	Population Variability: Posterior CU
lnkMetTCOHC	-2.3	-11.1	6.45	1.98	0.455	3.14	0.5	2	0.591	0.844
lnkUrnTCAC	0	-4.37	4.37	-2.11	-3.16	-1.27	0.5	2	0.69	0.635
lnkMetTCAC	-2.3	-8.86	4.26	-0.698	-1.34	0.0157	0.5	2	0.474	0.373
lnkBileC	-2.3	-8.86	4.26	0.863	-0.192	1.73	0.5	2	0.516	0.611
lnkEHRC	-2.3	-8.86	4.26	-3.25	-4.72	-2.39	0.5	2	0.565	0.554
lnkUrnTCOGC	0	-6.56	6.56	2.33	0.933	4.39	0.5	2	1.5	1.51
lnkDCVGC	-2.3	-8.86	4.26	2.19	0.061	4.4	0.5	2	0.535	0.75
lnkElimDCVCC	-2.3	-8.86	4.26	-1.14	-2.3	-0.0474	0.5	2	0.53	0.659
lnkClearDCAC	-2.3	-8.86	4.26	-2.57	-8.02	1.34	0.5	2	0.607	1.19
lnkDCAcen_perC	-2.3	-8.86	4.26	1.25	-5.28	4.28	0.5	2	0.618	1.12
lnkDCAper_cenC	-2.3	-8.86	4.26	-2.14	-5.73	1.72	0.5	2	0.66	0.98
lnkTSD	0.336	-4.04	4.71	1.36	-1.02	4.1	2	2	2.12	5.2
lnkAS	0.336	-6.23	6.9	-0.0762	-2.04	1.92	2	2	1.69	1.5
lnkAD	-0.288	-6.85	6.27	-1.34	-2.74	0.193	2	2	1.52	1.6
lnkASTCA	-0.288	-6.85	6.27	1.71	-2.24	5.55	2	2	2.05	2.3
lnkASTCOH	-0.288	-6.85	6.27	-0.361	-5.25	4.8	2	2	1.98	2.35
lnISOx	0	-4.37	4.37	-0.0614	-1.78	1.96	2	2	1.2	0.767
lnISTCA	0	-4.37	4.37	-1.83	-4.23	0.265	2	2	1.53	1.19
lnISDCA	0	-4.37	4.37	-0.649	-2.98	1.43	2	2	1.1	0.532
lnISConj	0	-4.37	4.37	-1.11	-3.29	0.723	2	2	1.58	1.36
lnISkTCA	0	-4.37	4.37	-0.19	-1.4	0.802	2	2	0.96	0.403
lnISkDCVG	0	-4.37	4.37	1.24	-0.709	3.37	2	2	1.64	1.15
lnISkDCVC	0	-4.37	4.37	-0.654	-2.18	1.22	2	2	1.53	1.09

Methods

Details of the Bayesian PBPK modeling of TCE and its metabolites

The structure of the updated PBPK model and the statistical population model are shown graphically in Figure 2 in the manuscript. Details as to the model structure and equations, and the likelihood function used in the parameter estimation, are given below.

PBPK model structure and equations

The equations below, along with the parameters defined in Supplemental Table S3, specify the PBPK model. The ordinary differential equations are shown in bold, with the remaining equations being algebraic definitions. The same equations are in the PBPK model code, with some additional provisions for unit conversions (e.g., ppm to mg/L) or numerical stability (e.g., truncating small values at 10^{-15}, so states are never negative). For reference, the stoichiometric adjustments for molecular weights are given by the following:

Molecular Weights

TCE: MWTCE = 131.39

DCA: MWDCA = 129.0

DCVC: MWDCVC = 216.1

TCA: MWTCA = 163.5

TCOH: MWTCOH = 149.5

TCOG: MWTCOHGluc = 325.53

Stoichiometry

StochTCATCE = MWTCA/MWTCE;

StochTCATCOH = MWTCA/MWTCOH;

StochTCOHTCE = MWTCOH/MWTCE;

StochGlucTCOH = MWTCOHGluc/MWTCOH;

StochTCOHGluc = MWTCOH/MWTCOHGluc;

StochTCEGluc = MWTCE/MWTCOHGluc;

StochDCVCTCE = MWDCVC/MWTCE

TCE submodel

The TCE submodel is a whole-body, flow-limited PBPK model, with gas respiratory exchange, oral absorption, and metabolizing and nonmetabolizing tissues.

Gas exchange, respiratory metabolism, arterial blood concentration, and closed-chamber concentrations

For an open-chamber concentration and a closed-chamber concentration of ACh/VCh, the rates of change for the amount in the respiratory lumen during inhalation (AInhResp, in mg), the amount in the respiratory tract tissue (AResp, in mg), and the respiratory lumen during exhalation (AExhResp, in mg) are given by the following:

d(AInhResp)/dt = (QM × CInh + DResp × (CResp – CInhResp) – QM × CInhResp) [Eq. 1]

d(AResp)/dt = (DResp × (CInhResp + CExhResp – 2 × CResp) – RAMetLng) [Eq. 1]

d(AExhResp)/dt = (QM × (CInhResp – CExhResp) + QP × (CArt_tmp/PB-CInhResp) + DResp × (CResp-CExhResp)) [Eq. 2]

where

CInh	= inhaled concentration (mg/L) = ACh/VCh + Conc
QM	= minute volume (L/hour) = QP/0.7
CInhResp	= concentration in respiratory lumen during inhalation (mg/L) = AInhResp/VRespLum
CResp	= concentration in respiratory tract tissue (mg/L) = AResp/VRespEff

CExhResp = concentration in respiratory lumen during exhalation (mg/L)
= AExhResp/VRespLum

RAMetLng = rate of metabolism in respiratory tract tissue
= (VMAXClara × CResp)/(KMClara + CResp)

CArt_tmp = arterial blood concentration after gas exchange
= (QC × CVen + QP × CInhResp)/(QC + (QP/PB))

Because alveolar breath concentrations can include desorption from the respiratory tract tissue, the concentration at the alveolae (CArt_tmp/PB) may not equal the measured concentration in end-exhaled breath. It is therefore assumed that the ratio of the measured end-exhaled breath concentration to the concentration in the absence of desorption is the same as the ratio of the rate of TCE leaving the lumen to the rate of TCE entering the lumen:

$$CAlv/(CArt\_tmp/PB) = (QM \times CMixExh)/\{(QP \times CArt\_tmp/PB + (QM-QP) \times CInhResp)\} \quad \text{[Eq. 3]}$$

That is, it is assumed that desorption occurs proportionally throughout the "breath." The concentration of arterial blood entering circulation needs to add the contribution from the i.a. dose (IADose in mg/kg, infused over a time period TChng):

$$CArt = CArt\_tmp + kIA/QC \quad \text{[Eq. 4]}$$

where

$$kIA = (IADose \times \text{body weight})/TChng$$

For closed-chamber experiments, the additional differential equation for the amount in the chamber (ACh, in mg) is:

$$d(ACh)/dt = Rodents \times (QM \times CMixExh - QM \times ACh/VCh) - kLoss \times Ach \quad \text{[Eq. 5]}$$

where rodents is the number of animals in the chamber, and kLoss is the chamber loss rate (per hour).

Oral absorption to gut compartment

For oil-based gavage, the dose PDose is defined in terms of units of mg/kg, entering the stomach during a time TChng, with rates of change in the stomach (AStom, in mg) and duodenum (ADuod, in mg):

$$d(AStom)/dt = kStom - AStom \times (kAS + kTSD) \quad \text{[Eq. 6]}$$

$$d(ADuod)/dt = (kTSD \times AStom) - kAD \times ADuod \quad \text{[Eq. 7]}$$

where

kStom = rate of TCE entering stomach (mg/hour) = (PDose × body weight)/TChng

Note that there is absorption to the gut from both the stomach and duodenal compartments. Analogous equations are defined for aqueous gavage, with the expectation that absorption and transfer coefficients would differ with the different vehicle. In particular, the aqueous gavage dose PDoseAq is defined in terms of units of mg/kg, entering the stomach during a time TChng, with rates of change in the stomach (AStomAq, in mg) and duodenum (ADuodAq, in mg):

$$d(AStomAq)/dt = kStomAq - AStomAq \times (kASAq + kTSDAq) \quad \text{[Eq. 8]}$$

$$d(ADuodAq)/dt = (kTSDAq \times AStomAq) - kADAq \times ADuodAq \quad \text{[Eq. 9]}$$

where

kStomAq = rate of TCE entering stomach (mg/hour) = (PDoseAq × body weight)/TChng

For drinking water, the rate Drink is defined in terms of mg/kg-day, and it is assumed that absorption is direct to the gut:

$$kDrink = (Drink \times \text{body weight})/24.0 \quad \text{[Eq. 10]}$$

Therefore, the total rate of absorption to the gut via oral exposure (RAO, in mg/hour) is:

$$RAO = kDrink + (kAS \times AStom) + (kAD \times ADuod) + (kASAq \times AStomAq) + (kADAq \times ADuodAq) \quad \text{[Eq. 11]}$$

The differential equation for the gut compartment (AGut, in mg) is, therefore, given by:

$$d(AGut)/dt = QGut \times (CArt - CVGut) + RAO \quad \text{[Eq. 12]}$$

where

CVGut = concentration in the gut (mg/L) = AGut/VGut/PGut

Nonmetabolizing tissues

The differential equations for nonmetabolizing tissues (rapidly perfused, ARap, in mg; slowly perfused, ASlw, in mg; fat, AFat, in mg; and kidney, AKid, in mg) follow the standard flow-limited form:

$$d(ARap)/dt = QRap \times (CArt - CVRap) \quad \text{[Eq. 13]}$$

$$d(ASlw)/dt = QSlw \times (CArt - CVSlw) \quad \text{[Eq. 14]}$$

$$d(AFat)/dt = QFat \times (CArt - CVFat) \quad \text{[Eq. 15]}$$

$$d(AKid)/dt = (QKid \times (CArt - CVKid)) \quad \text{[Eq. 16]}$$

where

CVRap = venous blood concentration leaving rapidly perfused issues
= ARap/VRap/PRap

CVSlw = venous blood concentration leaving slowly perfused issues
= ASlw/VSlw/PSlw

CVFat = venous blood concentration leaving fat
= AFat/VFat/PFat

CVKid = venous blood concentration leaving kidney
= AKid/VKid/PKid

Chiu et al. (2009) and Evans et al. (2009) included kidney GSH conjugation. For the current effort, it was determined to be non-identifiable as compared to liver GSH conjugation.

Liver compartment

The liver has two metabolizing pathways:

RAMetLiv1 = Rate of TCE oxidation by P450 in liver (mg/hour) [Eq. 17]
= (VMAX × CVLiv)/(KM + CVLiv)

RAMetLiv2 = Rate of TCE metabolized to S-dichlorovinyl glutathione (DCVG_ in liver (mg/hour)
= (VMAXDCVG × CVLiv) (KMDCVG + CVLiv) [Eq. 18]

Some experiments also had portal vein dosing (PVDose in mg/kg, infused over a time period TChng), with a rate entering the liver of:

kPV = (PVDose × body weight)/TChng [Eq. 19]

The differential equation for TCE in liver (ALiv, in mg) is thus:

d(ALiv)/dt = (QLiv × (CArt – CVLiv)) + (QGut × (CVGut – CVLiv)) – RAMetLiv1 – RAMetLiv2 + kPV [Eq. 20]

where

CVLiv = venous blood concentration leaving liver
= ALiv/VLiv/PLiv

Venous blood compartment

The venous blood compartment (ABld, in mg) has inputs both from the venous blood exiting tissues as well as from an IV dose (IVDose in mg/kg infused during a time TChng), and output to the gas exchange region:

d(ABld)/dt = (QFat × CVFat + QGutLiv × CVLiv + QSlw × CVSlw + QRap × CVRap + QKid × CVKid) + kIV – CVen × QC [Eq. 21]

where

kIV = IV infusion rate
= (IVDose × body weight)/TChng

CVen = concentration in mixed venous blood
= ABld/VBld

TCOH Submodel

The TCOH submodel is a simplified whole-body, flow-limited PBPK model, with only a body (ABodTCOH, in mg) and liver (ALivTCOH, in mg) compartment.

Blood concentration

The venous blood concentration, including an IV dose (IVDoseTCOH in mg/kg infused during a time TChng), is given by

$$\text{CTCOH} = (\text{QBod} \times \text{CVBodTCOH} + \text{QGutLiv} \times \text{CVLivTCOH} + \text{kIVTCOH})/\text{QC} \quad \text{[Eq. 22]}$$

where

$$\text{CVBodTCOH} = \text{ABodTCOH}/\text{VBodTCOH}/\text{PBodTCOH}$$

$$\text{CVLivTCOH} = \text{ALivTCOH}/\text{VLiv}/\text{PLivTCOH}$$

$$\text{kIVTCOH} = \text{IV infusion rate} = (\text{IVDoseTCOH} \times \text{body weight})/\text{TChng}$$

and the partition coefficients for the body:blood and liver:blood are PBodTCOH and PLivTCOH, respectively, QGutLiv is the sum of the portal vein and hepatic artery blood flows, QBod is the remaining blood flow, VLiv is the liver volume, and VBodTCOH is the remaining perfused volume.

Body compartment

The rate of change of the amount of TCOH in the body compartment is

$$d(\text{ABodTCOH})/dt = \text{QBod} \times (\text{CTCOH} - \text{CVBodTCOH}) \quad \text{[Eq. 23]}$$

Liver compartment

The liver has three metabolizing pathways:

RAMetTCOHTCA = Rate of oxidation of TCOH to TCA (mg/hour) [Eq. 24]
= (VMAXTCOH × CVLivTCOH)/(KMTCOH
+ CVLivTCOH)

RAMetTCOHGluc = Amount of glucuronidation to TCOG (mg/hour) [Eq. 25]
= (VMAXGluc × CVLivTCOH)/(KMGluc
+ CVLivTCOH)

RAMetTCOH = Amount of TCOH metabolized to other (e.g., DCA) [Eq. 26]
= kMetTCOH × ALivTCOH

Some experiments also had oral dosing (PODoseTCOH in mg/kg, entering the stomach over a time TChng):

d(AStomTCOH)/dt = kStomTCOH – AStomTCOH × kASTCOH [Eq. 27]

kStomTCOH = (PODoseTCOH × body weight)/TChng [Eq. 28]

kPOTCOH = AStomTCOH × kASTCOH [Eq. 29]

In addition, there are three additional sources of TCOH:

Production in the liver from TCE (a fraction of hepatic oxidation) [Eq. 30]
= (1.0 – FracOther – FracTCA) × StochTCOHTCE × RAMetLiv1

Production in the lung from TCE (a fraction of lung oxidation) [Eq. 31]
= (1.0 – FracOther – FracTCA) × StochTCOHTCE
× FracLungSys × RAMetLng

Enterohepatic recirculation (rate kEHR) from TCOG in the bile [Eq. 32]
(amount ABileTCOG) = StochTCOHGluc × RARecircTCOG
= StochTCOHGluc × kEHR × ABileTCOG

Note that StochTCOHTCE is the ratio of molecular weights of TCOH and TCE, StochTCOHGluc is the ratio of molecular weights of TCOH and TCOG, FracOther is the fraction of TCE oxidation not producing TCA or TCOH, FracTCA is the fraction of TCE

oxidation producing TCA, and FracLungSys is the fraction of lung TCE oxidation that is translocated to the liver and not locally cleared.

The differential equation for TCOH in liver (ALivTCOH, in mg) is thus:

$$d(\text{ALivTCOH})/dt = \text{kPOTCOH} + \text{QGutLiv} \times (\text{CTCOH} - \text{CVLivTCOH}) - \text{RAMetTCOH} - \text{RAMetTCOHTCA} - \text{RAMetTCOHGluc} + ((1.0 - \text{FracOther} - \text{FracTCA}) \times \text{StochTCOHTCE} \times (\text{RAMetLiv1} + \text{FracLungSys} \times \text{RAMetLng})) + (\text{StochTCOHGluc} \times \text{RARecircTCOG}) \quad \text{[Eq. 33]}$$

TCOG Submodel

The TCOG submodel is a simplified whole-body, flow-limited PBPK model, with body (ABodTCOG, in mg), liver (ALivTCOG, in mg), and bile (ABileTCOG) compartments.

Blood concentration

The venous blood concentration is given by:

$$\text{CTCOG} = (\text{QBod} \times \text{CVBodTCOG} + \text{QGutLiv} \times \text{CVLivTCOG})/\text{QC} \quad \text{[Eq. 34]}$$

where

$$\text{CVBodTCOG} = \text{ABodTCOG}/\text{VBodTCOH}/\text{PBodTCOG}$$

$$\text{CVLivTCOG} = \text{ALivTCOG}/\text{VLiv}/\text{PLivTCOG}$$

and the partition coefficients for the body:blood and liver:blood are PBodTCOG and PLivTCOG, respectively, QGutLiv is the sum of the portal vein and hepatic artery blood flows, QBod is the remaining blood flow, VLiv is the liver volume, and VBodTCOH is the remaining perfused volume.

Body compartment

The body compartment is flow limited, with urinary excretion rate (mg/hour):

$$\text{RUrnTCOG} = \text{kUrnTCOG} \times \text{ABodTCOG} \qquad \text{[Eq. 35]}$$

So the rate of change of the amount of TCOG in the body compartment is:

$$\text{d(ABodTCOG)/dt} = \text{QBod} \times (\text{CTCOG} - \text{CVBodTCOG}) - \text{RUrnTCOG} \qquad \text{[Eq. 36]}$$

Thus, the amount excreted in urine (AUrnTCOG, mg) is given by:

$$\text{d(AUrnTCOG)/dt} = \text{RUrnTCOG} \qquad \text{[Eq. 37]}$$

Liver compartment

The liver is flow limited, with one input, glucuronidation of TCOH (defined above in the TCOH submodel):

$$\text{StochGlucTCOH} \times \text{RAMetTCOHGluc} \qquad \text{[Eq. 38]}$$

and one additional output, excretion in bile:

$$\text{RBileTCOG} = \text{rate of excretion in bile (mg/hour)} = \text{kBile} \times \text{ALivTCOG} \qquad \text{[Eq. 39]}$$

The rate of change of the amount of TCOG in the liver is, therefore:

$$\begin{aligned}\text{d(ALivTCOG)/dt} = {} & \text{QGutLiv} \times (\text{CTCOG} - \text{CVLivTCOG}) \qquad \text{[Eq. 40]} \\ & + (\text{StochGlucTCOH} \times \text{RAMetTCOHGluc}) - \text{RBileTCOG}\end{aligned}$$

Bile compartment

The bile compartment has one input, excretion of TCOG in bile from the liver (defined above) and one output, enterohepatic recirculation to TCOH in the liver (defined above in the TCOH submodel), with rate of change:

$$\text{d(ABileTCOG)/dt} = \text{RBileTCOG} - \text{RARecircTCOG} \qquad \text{[Eq. 41]}$$

TCA Submodel

The TCA submodel is the same as that in Hack et al. (2006), with an error in the plasma flow to the liver corrected. In brief, TCA in plasma is assumed to undergo saturable plasma protein binding. TCA in tissues is assumed to be flow limited, but with the tissue partition coefficient reflecting equilibrium with the free concentration of TCA in plasma.

Plasma binding and concentrations

For an i.v. dose of TCA given by IVDoseTCA (mg/kg during an infusion period of TChng), the rate of the change of the amount of total TCA in plasma (APlasTCA, in mg) is:

$$d(APlasTCA)/dt = kIVTCA + (QBodPlas \times CVBodTCA) + (QGutLivPlas \times CVLivTCA) - (QCPlas \times CPlasTCA) - RUrnTCAplas \quad \text{[Eq. 42]}$$

where

kIVTCA = rate of IV infusion of TCA = (IVDoseTCA × body weight)/TChng

QBodPlas = plasma flow from body = QBod × FracPlas

QGutLivPlas = plasma flow from liver = (QGut + QLiv) × FracPlas

CVBodTCA = venous concentration leaving body = CPlasTCABnd + CVBodTCAFree

CVBodTCAFree = free venous concentration leaving body = (ABodTCA/VBod/PBodTCA)

CVLivTCA = venous concentration leaving liver = CPlasTCABnd + CVLivTCAFree

CVLivTCAFree = free venous concentration leaving liver = (ALivTCA/VLiv/PLivTCA)

QCPlas = total plasma flow = QC × FracPlas

RUrnTCAplas = rate of urinary excretion of TCA from plasma = kUrnTCA × APlasTCAFree

The free (CPlasTCAFree) and bound (CPlasTCABnd) concentrations are calculated from the total concentration (CPlasTCA = APlasTCA/VPlas) by solving the equations:

CPlasTCABndMole = BMax × CPlasTCAFreeMole/(kDissoc + CPlasTCAFreeMole) [Eq. 43]

CPlasTCABndMole = CPlasTCAMole – CPlasTCAFreeMole [Eq. 44]

Here the suffix "Mole" means that all concentrations are in micromole/L, because BMax and kDissoc are given in those units. These lead to explicit solutions of:

CPlasTCAFreeMole = (sqrt(a × a + b) – a)/2 [Eq. 45]

where

a = kDissoc + BMax – CPlasTCAMole

b = 4.0 × kDissoc × CPlasTCAMole

CPlasTCABlasTCAMoleCPlasTCAFreeMole

These concentrations are converted to mg/L (CPlasTCABnd, CPlasTCAFree) by multiplying by the molecular weight in mg/µmoles. The amount of free TCA in plasma is, thus:

APlasTCAFree = CPlasTCAFree × VPlas. [Eq. 46]

Here, VPlas is derived from the blood volume and hematocrit.

Urinary excretion

Urinary excretion is modeled as coming from the plasma compartment, so the rate of change of TCA in urine (AUrnTCA, in mg) is:

d(AUrnTCA)/dt = RUrnTCA [Eq. 47]

where

RUrnTCA = RUrnTCAplas

For some human data (Chiu et al. 2007), urinary excretion was only collected during certain time periods, with data missing in other time periods. Thus, a switch UrnMissing was defined, which equals 0 during times of urine collection and 1 when urinary data are missing (this parameter was not used for mice, and set to 0). The total amount of urinary TCA "collected" (AUrnTCA_collect, in mg) is, thus, given by:

d(AUrnTCA_collect)/dt = (1-UrnMissing) × RUrnTCA [Eq. 48]

Body compartment

The body compartment is flow limited, with the rate of change for the amount of TCA in the body (ABodTCA, in mg) given by:

d(ABodTCA)/dt = QBodPlas × (CPlasTCAFree – CVBodTCAFree) [Eq. 49]

Liver compartment

The rate of change for the amount of TCA in the liver (ALivTCA, in mg) is given by:

d(ALivTCA)/dt = QGutLivPlas × (CPlasTCAFree – CVLivTCAFree) [Eq. 50]
+ (FracTCA × StochTCATCE × (RAMetLiv1 + FracLungSys × RAMetLng))
+ (StochTCATCOH × RAMetTCOHTCA) – RAMetTCA + kPOTCA

The first term reflects the free TCA in plasma flowing into and out of the liver compartment, the second term reflects production of TCA from liver (adjusted for molecular weights and fractional yield of TCA) and lung (adjusted for molecular weights, fraction of lung metabolism translocated to the liver, and fractional yield of TCA) metabolism of TCE, the third term reflects production of TCA from TCOH, the fourth term reflects other clearance of TCA from the liver, and the fifth term reflects absorption from the stomach of TCA. The contribution from liver metabolism of TCE is adjusted for molecular weights and production of oxidative metabolites other than TCA. The rate of clearance of TCA is given by:

$$RAMetTCA = kMetTCA \times ALivTCA \quad [Eq. 51]$$

The oral intake rate of TCA (mg/hour) includes a one-compartment stomach. So for an oral dose of PODoseTCA (in mg/kg), occurring over a time TChng, the rate of change of TCA in the stomach (AStomTCA, in mg) is given by:

$$d(AStomTCA)/dt = kStomTCA - AStomTCA \times kASTCA \quad [Eq. 52]$$

where

kStomTCA = rate of input into stomach
= (PODoseTCA × body weight)/TChng

The rate of absorption into the liver is, thus,

$$kPOTCA = AStomTCA \times kASTCA \quad [Eq. 53]$$

GSH conjugation submodel

The GSH conjugation submodel only tracks DCVG, DCVC, and elimination of DCVC.

The rate of change for DCVG (ADCVGmol, in mmoles) depends on production from TCE in the liver and metabolism to DCVC:

$$d(ADCVGmol)/dt = RAMetLiv2/MWTCE - RAMetDCVGmol \quad [Eq. 54]$$

where

RAMetDCVGmol = rate of metabolism of DCVG to DCVC
= kDCVG × ADCVGmol

The rate of change of DCVC (ADCVC, in mg) depends on the production from DCVG and the lumped elimination rate of DCVC (rate constant kElimDCVC):

$$d(ADCVC)/dt = RAMetDCVGmol \times MWDCVC - kElimDCVC \times ADCVC \quad [Eq. 55]$$

DCA submodel

The DCA submodel is a two-compartment model with a central and peripheral compartment, with clearance (lumped metabolism and excretion) from the central compartment.

The rate of change for DCA in the central compartment (ADCA, in mg) depends on the production from TCE metabolism, the clearance (rate constant kClearDCA), and distribution to (rate constant kDCAcen_per) and from (rate constant kDCAper_cen) the peripheral compartment:

$$d(ADCA)/dt = (FracOther \times StochDCATCE \times (RAMetLiv1 + FracLungSys \times RAMetLng)) - (kClearDCA \times ADCA) - kDCAcen\_per \times ADCA + kDCAper\_cen \times ADCAper; \quad \text{[Eq. 56]}$$

The rate of change for DCA in the peripheral compartment (ADCAper, in mg) depends only on the distribution to and from the central compartment:

$$d(ADCAper)/dt = kDCAcen\_per \times ADCA - kDCAper\_cen \times A\ DCAper \quad \text{[Eq. 57]}$$

Likelihood function

In most cases, the likelihood function of the data given the predictions was assumed to be lognormal, which requires specification of the variance of the "residual error." This error may include variability due to measurement error, intra-individual and intra-study heterogeneity, as well as model misspecification. The variances for each of the corresponding residual errors were given log-uniform distributions. For all measurements, the bounds on the log-uniform distribution were 0.01 and 3.3, corresponding to geometric standard deviations bounded by 1.11 and 6.15. The lower bound was set to prevent "over-fitting" (Bois 2000; CE Hack et al. 2006). The upper bound was set at an arbitrarily high value, and the posteriors checked to make sure that the bound was unimportant.

For DCVG, DCVC, and DCA data, a different error model was necessary because many individual measurements were below the limit of quantitation. The use of a lognormal error model is based on the fact that analytical measurements typically have proportional errors well

above the detection limit. However, much of the data on DCVG, DCVC, and DCA are at or near the detection limit, where analytical errors are typically fixed and a normal distribution is more appropriate. Therefore, for these data, a "two-component error model" (Rocke and Lorenzato 1995) was used that is normally distributed near the detection limit (with a standard deviation fixed at one-third of the detection limit) and lognormally distributed well above the detection limit (with "residual error" specified as discussed previously). Specifically, the variance-stabilizing "generalized-log" transformation (Durbin and Rocke 2003) was employed for computing the likelihood.

References

Abbas R, Fisher J. 1997. A physiologically based pharmacokinetic model for trichloroethylene and its metabolites, chloral hydrate, trichloroacetate, dichloroacetate, trichloroethanol, and trichloroethanol glucuronide in B6C3F1 mice. Toxicol Appl Pharmacol 147(1):15-30.

Barter Z, Bayliss M, Beaune P, Boobis A, Carlile D, Edwards R, et al. 2007. Scaling factors for the extrapolation of in vivo metabolic drug clearance from in vitro data: Reaching a consensus on values of human microsomal protein and hepatocellularity per gram of liver. Curr Drug Metab 8(1):33-45.

Bronley-DeLancey A, McMillan D, McMillan J, Jollow D, Mohr L, Hoel D. 2006. Application of cryopreserved human hepatocytes in trichloroethylene risk assessment: Relative disposition of chloral hydrate to trichloroacetate and trichloroethanol. Environ Health Perspect 114(8):1237-1242.

Brown R, Delp M, Lindstedt S, Rhomberg L, Beliles R. 1997. Physiological parameter values for physiologically based pharmacokinetic models. Toxicol Ind Health 13(4):407-484.

Evans MV, Chiu WA, Okino MS, Caldwell JC. 2009. Development of an updated PBPK model for trichloroethylene and metabolites in mice, and its application to discern the role of oxidative metabolism in TCE-induced hepatomegaly. Toxicol Appl Pharmacol 236(3):329-340.

Elfarra A, Krause R, Last A, Lash L, Parker J. 1998. Species- and sex-related differences in metabolism of trichloroethylene to yield chloral and trichloroethanol in mouse, rat, and human liver microsomes. Drug Metab Dispos 26(8):779-785.

Fisher J, Gargas M, Allen B, Andersen M. 1991. Physiologically based pharmacokinetic modeling with trichloroethylene and its metabolite, trichloroacetic acid, in the rat and mouse. Toxicol Appl Pharmacol 109(2):183-195.

Green T, Mainwaring G, Foster J. 1997. Trichloroethylene-induced mouse lung tumors: Studies of the mode of action and comparisons between species. Fundam Appl Toxicol 37(2):125-130.

Hejtmancik M, Trela B, Kurtz P, Persing R, Ryan M, Yarrington J, et al. 2002. Comparative gavage subchronic toxicity studies of o-chloroaniline and m-chloroaniline in F344 rats and B6C3F1 mice. Toxicol Sci 69(1):234-243.

ICRP. 2003. Basic anatomical and physiological data for use in radiological protection: Reference values. (Annals of the ICRP). ICRP Publication 89. New York, NY: Pergamon Press.

Lash L, Visarius T, Sall J, Qian W, Tokarz J. 1998. Cellular and subcellular heterogeneity of glutathione metabolism and transport in rat kidney cells. Toxicology 130(1):1-15.

Lash L, Xu Y, Elfarra A, Duescher R, Parker J. 1995. Glutathione-dependent metabolism of trichloroethylene in isolated liver and kidney cells of rats and its role in mitochondrial and cellular toxicity. Drug Metab Dispos 23(8):846-853.

Lin Y, Brunt E, Bowling W, Hafenrichter D, Kennedy S, Flye M, et al. 1995. Ras-transduced diethylnitrosamine-treated hepatocytes develop into cancers of mixed phenotype in vivo. Can Res 55(22):5242-5250.

Lipscomb J, Fisher J, Confer P, Byczkowski J. 1998a. In vitro to in vivo extrapolation for trichloroethylene metabolism in humans. Toxicol Appl Pharmacol 152(2):376-387.

Lipscomb J, Garrett C, Snawder J. 1997. Cytochrome P450-dependent metabolism of trichloroethylene: Interindividual differences in humans. Toxicol Appl Pharmacol 142(2):311-318.

Lipscomb J, Garrett C, Snawder J. 1998b. Use of kinetic and mechanistic data in species extrapolation of bioactivation: Cytochrome P-450 dependent trichloroethylene metabolism at occupationally relevant concentrations. J Occup Health 40(2):110-117.

Lumpkin M, Bruckner J, Campbell J, Dallas C, White C, Fisher J. 2003. Plasma binding of trichloroacetic acid in mice, rats, and humans under cancer bioassay and environmental exposure conditions. Drug Metab Dispos 31(10):1203-1207.

Sarangapani R, Gentry P, Covington T, Teeguarden J, Clewell HJ I. 2003. Evaluation of the potential impact of age- and gender-specific lung morphology and ventilation rate on the dosimetry of vapors. Inhal Toxicol 15(10):987-1016.

Schultz I, Merdink J, Gonzalez-Leon A, Bull R. 1999. Comparative toxicokinetics of chlorinated and brominated haloacetates in F344 rats. Toxicol Appl Pharmacol 158(2):103-114.

Templin M, Parker J, Bull R. 1993. Relative formation of dichloroacetate and trichloroacetate from trichloroethylene in male B6C3F1 mice. Toxicol Appl Pharmacol 123(1):1-8.

Templin M, Stevens D, Stenner R, Bonate P, Tuman D, Bull R. 1995. Factors affecting species differences in the kinetics of metabolites of trichloroethylene. J Toxicol Environ Health 44(4):435-447.

Yu K, Barton H, Mahle D, Frazier J. 2000. In vivo kinetics of trichloroacetate in male Fischer 344 rats. Toxicol Sci 54(2):302-311.
