# Supplementary material for: Physiologically Based Pharmacokinetic (PBPK) Modeling of Interstrain Variability in Trichloroethylene Metabolism in the Mouse
Source: Environ Health Perspect. 2014 Feb 11;122(5):456–63. doi: 10.1289/ehp.1307623 (PMC4014769; doi:10.1289/ehp.1307623)
Supplement: (270 KB) PDF [file ehp.1307623.s002.pdf]

Environmental Health Perspectives doi:10.1289/ehp.1307623

Supplemental Material – PBPK Model Code

Physiologically Based Pharmacokinetic (PBPK) Modeling of Interstrain Variability in Trichloroethylene Metabolism in the Mouse

Weihsueh A. Chiu, Jerry L. Campbell Jr., Harvey J. Clewell 3rd, Yi-Hui Zhou, Fred A. Wright,

Kathryn Z. Guyton, and Ivan Rusyn

```
# TCE.risk.2.0.gamma.IS.pop.model -- TCE risk assessment PBPK model for
#                           multistrain mouse data
#
# Model code for MCSim, modified from TCE.risk.1.2.3.3.pop.model
published in
#
# Chiu WA, Okino MS, Evans MV. Characterizing uncertainty and population
# variability in the toxicokinetics of trichloroethylene and metabolites
# in mice, rats, and humans using an updated database, physiologically
# based pharmacokinetic (PBPK) model, and Bayesian approach. Toxicol
# Appl Pharmacol. 2009 Nov 15;241(1):36-60. doi:
10.1016/j.taap.2009.07.032.
# Epub 2009 Aug 4.
#
# Evans MV, Chiu WA, Okino MS, Caldwell JC. Development of an updated
# PBPK model for trichloroethylene and metabolites in mice, and its
# application to discern the role of oxidative metabolism in TCE-induced
# hepatomegaly. Toxicol Appl Pharmacol. 2009 May 1;236(3):329-40.
# doi: 10.1016/j.taap.2009.02.013. Epub 2009 Feb 26.
#
# Modifications are labeled with a comment "#(v2.0)"
#
#***********************************************************************
******
#***                  State Variable Specifications
***
#***********************************************************************
******

States = {
##-- TCE uptake
      AStom,            # Amount of TCE in stomach
      ADuod,            # oral gavage absorption -- mice and rats only
#(v1e)     AExc,        # excreted in feces from gavage -- mice and rats
only
#(v1e)     AO,          # total absorbed
      InhDose,    # Amount inhaled
##-- TCE in the body
      ARap,       # Amount in rapidly perfused tissues
      ASlw,       # Amount in slowly perfused tissues
      AFat,       # Amount in fat
      AGut,       # Amount in gut
      ALiv,       # Amount in liver
#(v1j)     ATB,         # Amount in TB -- candidate to remove and put in
Rap
                        # comment out for #(v1b)(v1c)
#(v1.2)    RTB,  #(v1j) Rate of metabolism in TB
      AKid,             # Amount in Kidney -- previously in Rap tissue
      ABld,             # Amount in Blood -- previously in Rap tissue
                        # comment out for #(v1a)(v1c)
      AInhResp,   #(v1.2) Amount in respiratory lumen during inhalation
      AResp,            #(v1.2) Amount in respiratory tissue
      AExhResp,   #(v1.2) Amount in respiratory lumen during exhalation
##-- DCA in body
```

```
        ADCA,             # Amount in central compartment#(v2.0)
        ADCAper, # Amount in peripheral compartment#(v2.0)
#(v1e)  AElimDCA,# Amount eliminated#(v2.0)
#
##-- TCA in the body
#(v1e)     AOTCA,
      AStomTCA,  # Amount of TCA in stomach
      APlasTCA,  # Amount of TCA in plasma #comment out for #(v1h)
      ABodTCA,   # Amount of TCA in lumped body compartment
      ALivTCA,   # Amount of TCA in liver
##-- TCA metabolized
      AUrnTCA,   # Cumulative Amount of TCA excreted in urine
      AUrnTCA_sat,     # Amount of TCA excreted that during times that
had
                 # saturated measurements (for lower bounds)
      AUrnTCA_collect,# Cumulative Amount of TCA excreted in urine during
                 # collection times (for intermittent collection)
##-- TCOH in body
#(v1e)     AOTCOH,
      AStomTCOH, # Amount of TCOH in stomach
      ABodTCOH,  # Amount of TCOH in lumped body compartment
      ALivTCOH,  # Amount of TCOH in liver
##-- TCOG in body
      ABodTCOG,  # Amount of TCOG in lumped body compartment
      ALivTCOG,  # Amount of TCOG in liver
      ABileTCOG, # Amount of TCOG in bile (incl. gut)
#(v1e)     ARecircTCOG,
##-- TCOG excreted
      AUrnTCOG,  # Amount of TCOG excreted in urine
      AUrnTCOG_sat,    # Amount of TCOG excreted that during times that
had
                 # saturated measurements (for lower bounds)
      AUrnTCOG_collect,# Cumulative Amount of TCA excreted in urine
during
                 # collection times (for intermittent collection)
##-- DCVG in body
#(v1e)     ADCVGIn,
      ADCVGmol,  # Amount of DCVG in body in mmoles
#(v1e)     AMetDCVG,
##-- DCVC in body
#(v1e)     ADCVCIn,
        ADCVC,         # Amount of DCVC in body
#(v2.0) deleted ABioactDCVC,
##-- DCVC eliminated#(v2.0)
#(v1e)     AElimDCVC, # Amount of DCVC eliminated#(v2.0)
##-- Other states for TCE
      ACh,       # Amount in closed chamber -- mice and rats only
      AExh,            # Amount exhaled
      AExhExp,   # Amount exhaled during expos  [to calc. retention]
##-- Metabolism
      AMetLiv1,  # Amount metabolized by P450 in liver#(vrisk)
      AMetLiv2,  # Amount metabolized by GSH conjugation in liver#(vrisk)
      AMetLng,   # Amount metabolized in the lung#(vrisk)
      AMetKid,#(vrisk)
```

```
      AMetTCOHTCA,     # Amount of TCOH metabolized to TCA#(vrisk)
#(v1e)      AMetTCOHGluc,    # Amount of TCOH glucuronidated
#(v1e)      AMetTCOHOther,
#(v1e)      AMetTCA,   # Amount of TCA metabolized
##-- Other Dose metrics
#(v1d)      AUCCBld,   #
#(v1d)      AUCCLiv,   #
#(v1d)      AUCCKid,   #
#(v1d)      AUCCFat,   ##
#(v1d)      AUCCRap,   ##
#(v1d)      AUCCSlw,   ##
#(v1d)      AUCCGut,   ##
#(v1d)      AUCCChl,   #
#(v1d)      AUCCTCOH,  #
#(v1d)      AUCCTCOG,  #
#(v1d)      AUCPlasTCAFree,#
#(v1d)      AUCPlasTCA #
};

#***********************************************************************
******
#***                  Input Variable Specifications
***
#***********************************************************************
******

Inputs = {
##-- TCE dosing
      Conc,       # Inhalation exposure conc. (ppm)
      IVDose,           # IV dose (mg/kg)
      PDose,            # Oral gavage dose (mg/kg)
      Drink,            # Drinking water dose (mg/kg/day)
      IADose,                 # Inter-arterial (v1f)
      PVDose,                 # Portal Vein (v1f)
##-- TCA dosing
      IVDoseTCA, # IV dose (mg/kg) of TCA
      PODoseTCA, # Oral dose (mg/kg) of TCA
##-- TCOH dosing
      IVDoseTCOH,# IV dose (mg/kg) of TCOH
      PODoseTCOH,# Oral dose (mg/kg) of TCOH
##-- Potentially time-varying parameters
      QPmeas,           # Measured value of Alveolar ventilation QP
      TCAUrnSat, # Flag for saturated TCA urine
      TCOGUrnSat,# Flag for saturated TCOG urine
      UrnMissing # Flag for missing urine collection times
};

#***********************************************************************
******
#***                  Output Variable Specifications
***
#***********************************************************************
******
Outputs = {
```

```
#*****************************************************************************
#*** Outputs for mass balance check
MassBalTCE,
MassBalTCOH,
MassBalDCA,#(v2.0)
MassBalTCA,
MassBalTCOG,
MassBalDCVG,
MassBalDCVC,

#*****************************************************************************
#*** Outputs for comparison to in vivo data
# TCE
#(v1.2) AlvRet,  # human - Fractional alveolar retention  = (CInh-CAlv)/CInh
#(v1.2)          # = 1 - AExhExp/InhDose
RetDose,    # human - = (InhDose - AExhExp)
CAlv, # needed for CAlvPPM
CAlvPPM,    # human
CInhPPM,    # mouse, rat
CInh, # needed for CMixExh
CMixExh,    # rat - Mixed exhaled breath (mg/l)
CArt, # rat, human - Arterial blood concentration
CVen, # mouse, rat, human
CBldMix,    # rat - Concentration in mixed arterial+venous blood
            # (used for cardiac puncture)
CFat, # mouse, rat - Concentration in fat
CGut, # rat
CRap, # needed for unlumped tissues
CSlw, # needed for unlumped tissues
#(v1.2) CTB,     # needed for lung
CHrt, # rat - Concentration in heart tissue [use CRap]
CKid, # mouse, rat -  Concentration in kidney
CLiv, # mouse, rat - Concentration in liver
CLung,      # mouse, rat - Concentration in lung [use CRap]
CMus, # rat - Concentration in muscle [use CSlw]
CSpl,       # rat - Concentration in spleen [use CRap]
CBrn,       # rat - Concentration in brain [use CRap]
zAExh,      # mouse
zAExhpost,  # rat - Amount exhaled post-exposure (mg)

# TCOH
CTCOH,      # mouse, rat, human - TCOH concentration in blood
CKidTCOH,   # mouse - TCOH concentration in kidney
CLivTCOH,   # mouse - TCOH concentration in liver
CLungTCOH,  # mouse - TCOH concentration in lung

# TCA
CPlasTCA,   # mouse, rat, human - TCA concentration in plasma
CBldTCA,    # mouse, rat, human - TCA concentration in blood
CBodTCA,    # needed for CKidTCA and CLungTCA
CKidTCA,    # mouse - TCA concentration in kidney
```

```
CLivTCA,    # mouse, rat - TCA concentration in liver
CLungTCA,   # mouse - TCA concentration in lung
zAUrnTCA,   # mouse, rat, human - Cumulative Urinary TCA
zAUrnTCA_collect,# human - TCA measurements for intermittent collection
zAUrnTCA_sat,     # human - Saturated TCA measurements

# TCOG
zABileTCOG, # rat - Amount of TCOG in bile (mg)
CTCOG,      # needed for CTCOGTCOH
CTCOGTCOH,  # mouse - TCOG concentration in blood (in TCOH-equiv)
CKidTCOGTCOH,     # mouse - TCOG concentration in kidney (in TCOH-equiv)
CLivTCOGTCOH,     # mouse - TCOG concentration in liver (in TCOH-equiv)
CLungTCOGTCOH,    # mouse - TCOG concentration in lung (in TCOH-equiv)
AUrnTCOGTCOH,     # mouse, rat, human - Cumulative Urinary TCOG (in TCOH-
equiv)
AUrnTCOGTCOH_collect,  # human - TCOG (in TCOH-equiv) measurements for
                  # intermittent collection
AUrnTCOGTCOH_sat,# human - Saturated TCOG (in TCOH-equiv) measurements

# Other
CDCVGmol,         # concentration of DCVG (mmol/l)
CDCVGmol0,  # Dummy variable without likelihood (for plotting)
CDCVG_ND,   # Non-detect of DCVG (<0.001 nmol/ml= 1e-6 mmol/l )#(v2.0)
            # Output -ln(likelihood)
CDCVCmol,         # concentration of DCVC (mmol/l) #(v2.0)
CDCVCmol0,  # Dummy variable without likelihood (for plotting)#(v2.0)
CDCVC_ND,   # Non-detect of DCVC (<0.001 nmol/ml= 1e-6 mmol/l )#(v2.0)
            # Output -ln(likelihood)#(v2.0)
CDCAmol,          # concentration of DCA (mmol/l) #(v2.0)
CDCAmol0,   # Dummy variable without likelihood (for plotting)#(v2.0)
CDCA_ND,    # Non-detect of DCA (<0.01 nmol/ml= 1e-5 mmol/l )#(v2.0)
            # Output -ln(likelihood)#(v2.0)
AUrnTCTotMole,    # rat, human - Cumulative urinary TCOH+TCA in mmoles
TotCTCOH,   # mouse, human - TCOH+TCOG Concentration (in TCOH-equiv)
TotCTCOHcomp,     # ONLY FOR COMPARISON WITH HACK
ATCOG,            # ONLY FOR COMPARISON WITH HACK
QPsamp,     # human - sampled value of alveolar ventilation rate

      AMetOX,           # Amount of TCE oxidized #(vrisk)
      AMetGSH,    # Amount of TCE conjugated #(vrisk)
      TotTCAProd, # Amount TCA produced in mg TCA #(vrisk)
      TotDCAProd, # Amount DCA produced in mg DCA #(vrisk)


## PARAMETERS #(vrisk3)
  QCnow, # (vrisk3) #Cardiac output (L/hr)
  QP, # (vrisk3) #Alveolar ventilation (L/hr)
  QFatSC, # (vrisk3) #Scaled fat blood flow
  QGutSC, # (vrisk3) #Scaled gut blood flow
  QLivSC, # (vrisk3) #Scaled liver blood flow
  QSlwSC, # (vrisk3) #Scaled slowly perfused blood flow
  QRapSC, # (vrisk3) #Scaled rapidly perfused blood flow
  QKidSC, # (vrisk3) #Scaled kidney blood flow
  DResp, # (vrisk3) #Respiratory lumen:tissue diffusive clearance rate
```

```
  VFatSC, # (vrisk3) #Fat fractional compartment volume
  VGutSC, # (vrisk3) #Gut fractional compartment volume
  VLivSC, # (vrisk3) #Liver fractional compartment volume
  VRapSC, # (vrisk3) #Rapidly perfused fractional compartment volume
  VRespLumSC, # (vrisk3) # Fractional volume of respiratory lumen
  VRespEffSC, # (vrisk3) #Effective fractional volume of respiratory
tissue
  VKidSC, # (vrisk3) #Kidney fractional compartment volume
  VBldSC, # (vrisk3) #Blood fractional compartment volume
  VSlwSC, # (vrisk3) #Slowly perfused fractional compartment volume
  VPlasSC, # (vrisk3) #Plasma fractional compartment volume
  VBodSC, # (vrisk3) #TCA Body fractional compartment volume [not incl.
blood+liver]
  VBodTCOHSC, # (vrisk3) #TCOH/G Body fractional compartment volume [not
incl. liver]
  PB, # (vrisk3) #TCE Blood/air partition coefficient
  PFat, # (vrisk3) #TCE Fat/Blood partition coefficient
  PGut, # (vrisk3) #TCE Gut/Blood partition coefficient
  PLiv, # (vrisk3) #TCE Liver/Blood partition coefficient
  PRap, # (vrisk3) #TCE Rapidly perfused/Blood partition coefficient
  PResp, # (vrisk3) #TCE Respiratory tissue:air partition coefficient
  PKid, # (vrisk3) #TCE Kidney/Blood partition coefficient
  PSlw, # (vrisk3) #TCE Slowly perfused/Blood partition coefficient
  TCAPlas, # (vrisk3) #TCA blood/plasma concentration ratio
  PBodTCA, # (vrisk3) #Free TCA Body/blood plasma partition coefficient
  PLivTCA, # (vrisk3) #Free TCA Liver/blood plasma partition coefficient
  kDissoc, # (vrisk3) #Protein/TCA dissociation constant (umole/L)
  BMax, # (vrisk3) #Maximum binding concentration (umole/L)
  PBodTCOH, # (vrisk3) #TCOH body/blood partition coefficient
  PLivTCOH, # (vrisk3) #TCOH liver/body partition coefficient
  PBodTCOG, # (vrisk3) #TCOG body/blood partition coefficient
  PLivTCOG, # (vrisk3) #TCOG liver/body partition coefficient
  VDCVG, # (vrisk3) #DCVG effective volume of distribution
  VDCVC, # (v2.0risk)
  VDCA, # (v2.0risk)
  kAS, # (vrisk3) #TCE Stomach absorption coefficient (/hr)
  kTSD, # (vrisk3) #TCE Stomach-duodenum transfer coefficient (/hr)
  kAD, # (vrisk3) #TCE Duodenum absorption coefficient (/hr)
  kTD, # (vrisk3) #TCE Duodenum-feces transfer coefficient (/hr)
  kASTCA, # (vrisk3) #TCA Stomach absorption coefficient (/hr)
  kASTCOH, # (vrisk3) #TCOH Stomach absorption coefficient (/hr)
  VMax, # (vrisk3) #VMax for hepatic TCE oxidation (mg/hr)
  KM, # (vrisk3) #KM for hepatic TCE oxidation (mg/L)
  FracOther, # (vrisk3) #Fraction of hepatic TCE oxidation not to
TCA+TCOH
  FracTCA, # (vrisk3) #Fraction of hepatic TCE oxidation to TCA
  VMaxDCVG, # (vrisk3) #VMax for hepatic TCE GSH conjugation (mg/hr)
  KMDCVG, # (vrisk3) #KM for hepatic TCE GSH conjugation (mg/L)
  ClDCVG, # (vrisk3) #CL for hepatic TCE GSH conjugation (L/hr)
  VMaxKidDCVG, # (vrisk3) #VMax for renal TCE GSH conjugation (mg/hr)
  KMKidDCVG, # (vrisk3) #KM for renal TCE GSH conjugation (mg/L)
  FracKidDCVC, # (vrisk3) #Fraction of renal TCE GSH conj. "directly" to
DCVC
               # (vrisk3) #(i.e., via first pass)
```

```
  VMaxClara, # (vrisk3) #VMax for Tracheo-bronchial TCE oxidation (mg/hr)
  KMClara, # (vrisk3) #KM for Tracheo-bronchial TCE oxidation (mg/L)
  FracLungSys, # (vrisk3) #Fraction of respiratory metabolism to systemic
circ.
  kClearDCA,     # Rate constant for DCA elimination (/hr)#(v2.0risk)
  kDCAcen_per,   # Rate constant for DCA central->peripheral
(/hr)#(v2.0risk)
  kDCAper_cen,   # Rate constant for DCA peripheral->central
(/hr)#(v2.0risk)
  VMaxTCOH, # (vrisk3) #VMax for hepatic TCOH->TCA (mg/hr)
  KMTCOH, # (vrisk3) #KM for hepatic TCOH->TCA (mg/L)
  VMaxGluc, # (vrisk3) #VMax for hepatic TCOH->TCOG (mg/hr)
  KMGluc, # (vrisk3) #KM for hepatic TCOH->TCOG (mg/L)
  kMetTCOH, # (vrisk3) #Rate constant for hepatic TCOH->other (/hr)
  kUrnTCA, # (vrisk3) #Rate constant for TCA plasma->urine (/hr)
  kMetTCA, # (vrisk3) #Rate constant for hepatic TCA->other (/hr)
  kBile, # (vrisk3) #Rate constant for TCOG liver->bile (/hr)
  kEHR, # (vrisk3) #Lumped rate constant for TCOG bile->TCOH liver (/hr)
  kUrnTCOG, # (vrisk3) #Rate constant for TCOG->urine (/hr)
  kDCVG, # (vrisk3) #Rate constant for hepatic DCVG->DCVC (/hr)
  kElimDCVC,     # Lumped rate constant for DCVC elimination
(/hr)#(v2.0risk)

## Misc
  RAO,
  CVenMole,
  CPlasTCAMole,
  CPlasTCAFreeMole #(v1d), #

#***********************************************************************
******
#*** Outputs that are dose metrics
#(v1d)  AMetLiv1BW,    # Amount of oxidative metabolism in liver/liver
volume
#(v1d)  RiskKid  # Amount of DCVC bioactivated/kidney volume

};

#***********************************************************************
******
#***                 Global Constants
***
#***********************************************************************
******

# Molecular Weights
        MWTCE = 131.39;        # TCE
        MWDCA = 129.0;         # DCA
       MWDCVC = 216.1;         # DCVC
        MWTCA = 163.5;         # TCA
      MWChlor = 147.5;         # Chloral
       MWTCOH = 149.5;         # TCOH
   MWTCOHGluc = 325.53;        # TCOH-Gluc
     MWNADCVC = 258.8;         # N Acetyl DCVC
```

```
# Stoichiometry
 StochChlorTCE = MWChlor / MWTCE;
   StochDCATCE = MWDCA / MWTCE;
   StochTCATCE = MWTCA / MWTCE;
  StochTCATCOH = MWTCA / MWTCOH;
  StochTCOHTCE = MWTCOH / MWTCE;
 StochGlucTCOH = MWTCOHGluc / MWTCOH;
 StochTCOHGluc = MWTCOH / MWTCOHGluc;
  StochTCEGluc = MWTCE / MWTCOHGluc;
  StochDCVCTCE = MWDCVC / MWTCE;
        StochN = MWNADCVC / MWDCVC;

#*************************************************************************
******
#***                  Global Model Parameters
***
#*************************************************************************
******
# These are the actual model parameters used in "dynamics."
# Values that are assigned in the "initialize" section,
# are all set to 1 to avoid confusion.

#*************************************************************************
******
# Flows
QC    = 1;  # Cardiac output (L/hr)
QPsamp      = 1;  # Alveolar ventilation (L/hr)
VPR   = 1;  # Alveolar ventilation-perfusion ratio
QFatCtmp    = 1;  # Scaled fat blood flow
QGutCtmp    = 1;  # Scaled gut blood flow
QLivCtmp    = 1;  # Scaled liver blood flow
QSlwCtmp    = 1;  # Scaled slowly perfused blood flow
#(v1.2) QTBCtmp  = 1;  # Scaled tracheo-bronchial blood flow
DResptmp    = 1;  #(v1.2) Respiratory lumen:tissue diffusive clearance
rate (L/hr) [scaled to QP]
QKidCtmp    = 1;  # Scaled kidney blood flow
FracPlas    = 1;  # Fraction of blood that is plasma (1-hematocrit)
#*************************************************************************
******
# Volumes
VFat  = 1;  # Fat compartment volume (L)
VGut  = 1;  # Gut compartment volume (L)
VLiv  = 1;  # Liver compartment volume (L)
VRap  = 1;  # Rapidly perfused compartment volume (L)
#(v1.2) VTB = 1;  # Tracheo-bronchial compartment volume (L)
VRespLum    = 1;  #(v1.2) Volume of respiratory lumen (L air)
VRespEff    = 1;  #(v1.2) Effective volume for respiratory tissue (L air)
= V(tissue) * Resp:Air partition coefficient
VKid  = 1;  # Kidney compartment volume (L)
VBld  = 1;  # Blood compartment volume (L)
VSlw  = 1;  # Slowly perfused compartment volume (L)
VPlas = 1;  # Plasma compartment volume [fraction of blood] (L)
VBod  = 1;  # TCA Body compartment volume [not incl. blood+liver] (L)
```

```
VBodTCOH    = 1;  # TCOH/G Body compartment volume [not incl. liver] (L)
#*********************************************************************
******
# Distribution/partitioning
PB    = 1;  # TCE Blood/air partition coefficient
PFat  = 1;  # TCE Fat/Blood partition coefficient
PGut  = 1;  # TCE Gut/Blood partition coefficient
PLiv  = 1;  # TCE Liver/Blood partition coefficient
PRap  = 1;  # TCE Rapidly perfused/Blood partition coefficient
#(v1.2) PTB = 1;  # TCE Tracheo-bronchial/Blood partition coefficient
PResp = 1;  #(v1.2) TCE Respiratory tissue:air partition coefficient
PKid  = 1;  # TCE Kidney/Blood partition coefficient
PSlw  = 1;  # TCE Slowly perfused/Blood partition coefficient
TCAPlas     = 1;  # TCA blood/plasma concentration ratio
PBodTCA     = 1;  # Free TCA Body/blood plasma partition coefficient
PLivTCA     = 1;  # Free TCA Liver/blood plasma partition coefficient
kDissoc     = 1;  # Protein/TCA dissociation constant (umole/L)
BMax  = 1;  # Protein concentration (UNITS?)
PBodTCOH    = 1;  # TCOH body/blood partition coefficient
PLivTCOH    = 1;  # TCOH liver/body partition coefficient
PBodTCOG    = 1;  # TCOG body/blood partition coefficient
PLivTCOG    = 1;  # TCOG liver/body partition coefficient
VDCVG = 1;  # DCVG effective volume of distribution
VDCVC = 1;  # DCVC effective volume of distribution#(v2.0)
VDCA  = 1;  # DCA effective volume of distribution (central
compartment)#(v2.0)
#*********************************************************************
******
# Oral absorption
kTSD  = 1.4;      # TCE Stomach-duodenum transfer coefficient (/hr)
kAS   = 1.4;      # TCE Stomach absorption coefficient (/hr)
kTD   = 0.1;      # TCE Duodenum-feces transfer coefficient (/hr)
kAD   = 0.75;     # TCE Duodenum absorption coefficient (/hr)
kASTCA      = 0.75;     # TCA Stomach absorption coefficient (/hr)
kASTCOH     = 0.75;     # TCOH Stomach absorption coefficient (/hr)
#*********************************************************************
******
# TCA and DCA background
TCABgd = 0; # mg/kg/d
DCABgd = 0; # mg/kg/d
#*********************************************************************
******
# TCE Metabolism
VMax  = 1;  # VMax for hepatic TCE oxidation (mg/hr)
KM    = 1;  # KM for hepatic TCE oxidation (mg/L)
FracOther   = 1;  # Fraction of hepatic TCE oxidation not to TCA+TCOH
FracTCA     = 1;  # Fraction of hepatic TCE oxidation to TCA
VMaxDCVG    = 1;  # VMax for hepatic TCE GSH conjugation (mg/hr)
KMDCVG      = 1;  # KM for hepatic TCE GSH conjugation (mg/L)
VMaxKidDCVG = 1;  # VMax for renal TCE GSH conjugation (mg/hr)
KMKidDCVG   = 1;  # KM for renal TCE GSH conjugation (mg/L)
VMaxClara   = 1;  # VMax for Tracheo-bronchial TCE oxidation (mg/hr)
KMClara     = 1;  # KM for Tracheo-bronchial TCE oxidation (mg/L)
            #(v1.2) but in units of air concentration
```

```
#(v1.2) VMaxClear= 1;  # VMax for Tracheo-bronchial CH clearance (mg/hr)
#(v1.2) KMClear  = 1;  # KM for Tracheo-bronchial CH clearance (mg/L)
FracLungSys = 1;  #(v1.2) Fraction of respiratory oxidative metabolism
that enters systemic circulation

#*************************************************************************
******
# DCA metabolism/clearance#(v2.0)
kClearDCA   = 1;  # Rate constant for DCA elimination (/hr)#(v2.0)
kDCAcen_per = 1;  # Rate constant for DCA central->peripheral (/hr)#(v2.0)
kDCAper_cen = 1;  # Rate constant for DCA peripheral->central (/hr)#(v2.0)
#*************************************************************************
******
# TCOH metabolism
VMaxTCOH    = 1;  # VMax for hepatic TCOH->TCA (mg/hr)
KMTCOH      = 1;  # KM for hepatic TCOH->TCA (mg/L)
VMaxGluc    = 1;  # VMax for hepatic TCOH->TCOG (mg/hr)
KMGluc      = 1;  # KM for hepatic TCOH->TCOG (mg/L)
kMetTCOH    = 1;  # Rate constant for hepatic TCOH->other (/hr)
#*************************************************************************
******
# TCA metabolism/clearance
kUrnTCA     = 1;  # Rate constant for TCA plasma->urine (/hr)
kMetTCA     = 1;  # Rate constant for hepatic TCA->other (/hr)
#*************************************************************************
******
# TCOG metabolism/clearance
kBile = 1;  # Rate constant for TCOG liver->bile (/hr)
kEHR  = 1;  # Lumped rate constant for TCOG bile->TCOH liver (/hr)
kUrnTCOG    = 1;  # Rate constant for TCOG->urine (/hr)
#*************************************************************************
******
# DCVG metabolism
kDCVG = 1;  # Rate constant for hepatic DCVG->DCVC (/hr)
FracKidDCVC = 1;  # Fraction of renal TCE GSH conj. "directly" to DCVC
(i.e., via first pass)
#*************************************************************************
******
# DCVC metabolism/clearance
kElimDCVC   = 1;  # Lumped rate constant for DCVC elimination (/hr)#(v2.0)
#*************************************************************************
******
# Closed chamber and other exposure parameters
Rodents     = 1;  # Number of rodents in closed chamber data
VCh   = 1;  # Chamber volume for closed chamber data
kLoss = 1;  # Rate constant for closed chamber air loss
CC    = 0.0;      # Initial chamber concentration (ppm)
TChng       = 0.003;    # IV infusion duration (hour)
#*************************************************************************
******
## Flag for species, sex -- these are global parameters
BW    = 0.0;  # Species-specific defaults during initialization
Male  = 1.0;      # 1 = male, 0 = female
Species     = 1.0;      # 1 = human, 2 = rat, 3 = mouse
```

```
#***********************************************************************
******
#***                    Potentially measured covariates (constants)
***
#***********************************************************************
******
BWmeas      = 0.0;      # Body weight
VFatCmeas   = 0.0; # Fractional volume fat
PBmeas      = 0.0;      # Measured blood-air partition coefficient
Hematocritmeas = 0.0; # Measured hematocrit -- used for FracPlas = 1 -
HCt
CDCVGmolLD = 1e-6; # Detection limit of CDCVGmol (in mmol/L)#(v2.0)
CDCVCmolLD = 1e-6; # Detection limit of CDCVCmol (in mmol/L)#(v2.0)
CDCAmolLD = 1e-5; # Detection limit of CDCAmol (in mmol/L)#(v2.0)

#***********************************************************************
******
#***                    Global Sampling Parameters
***
#***********************************************************************
******
# These parameters are potentially sampled/calibrated in the MCMC or MC
# analyses.  The default values here are used if no sampled value is
given.
# M_ indicates population mean parameters used only in MC sampling
# V_ indicates a population variance parameter used in MC and MCMC
sampling

# Flow Rates
lnQCC = 0.0;      # Scaled by BW^0.75 and species-specific central
estimates
lnVPRC      = 0.0;      # Scaled to species-specific central estimates

# Fractional Blood Flows to Tissues (fraction of cardiac output)
QFatC = 1.0;      # Scaled to species-specific central estimates
QGutC = 1.0;      # Scaled to species-specific central estimates
QLivC = 1.0;      # Scaled to species-specific central estimates
QSlwC = 1.0;      # Scaled to species-specific central estimates
#(v1.2) QTBC      = 1.0;      # Scaled to species-specific central
estimates
QKidC = 1.0;      # Scaled to species-specific central estimates
FracPlasC  = 1.0;      # Scaled to species-specific central estimates
lnDRespC   = 0.0;      #(v1.2) Scaled to alveolar ventilation rate in
dynamics

# Fractional Tissue Volumes (fraction of BW)
VFatC = 1.0;      # Scaled to species-specific central estimates
VGutC = 1.0;      # Scaled to species-specific central estimates
VLivC = 1.0;      # Scaled to species-specific central estimates
VRapC = 1.0;      # Scaled to species-specific central estimates
#(v1.2) VTBC      = 1.0;      # Scaled to species-specific central
estimates
```

```
VRespLumC  = 1.0;      #(v1.2) Scaled to species-specific central
estimates
VRespEffC  = 1.0;      #(v1.2) Scaled to species-specific central
estimates

VKidC = 1.0;      # Scaled to species-specific central estimates
VBldC = 1.0;      # Scaled to species-specific central estimate

# Partition Coefficients for TCE
lnPBC = 0.0;      # Scaled to species-specific central estimates
lnPFatC    = 0.0;      # Scaled to species-specific central estimates
lnPGutC    = 0.0;      # Scaled to species-specific central estimates
lnPLivC    = 0.0;      # Scaled to species-specific central estimates
lnPRapC    = 0.0;      # Scaled to species-specific central estimates
#(v1.2) lnPTBC   = 0.0;      # Scaled to species-specific central
estimates
lnPRespC   = 0.0;      #(v1.2) Scaled to species-specific central
estimates
lnPKidC    = 0.0;      # Scaled to species-specific central estimates
lnPSlwC    = 0.0;      # Scaled to species-specific central estimates

# Partition Coefficients for TCA
lnPRBCPlasTCAC   = 0.0;      # Scaled to species-specific central
estimates
lnPBodTCAC = 0.0;      # Scaled to species-specific central estimates
lnPLivTCAC = 0.0;      # Scaled to species-specific central estimates

# Plasma Binding for TCA
lnkDissocC = 0.0;      # Scaled to species-specific central estimates
lnBMaxkDC  = 0.0;      # Scaled to species-specific central estimates

# Partition Coefficients for TCOH and TCOG
lnPBodTCOHC = 0.0;      # Scaled to species-specific central estimates
lnPLivTCOHC = 0.0;      # Scaled to species-specific central estimates
lnPBodTCOGC = 0.0;      # Scaled to species-specific central estimates
lnPLivTCOGC = 0.0;      # Scaled to species-specific central estimates
lnPeffDCVG  = 0.0;      # Scaled to species-specific central estimates
lnPeffDCVC  = 0.0;      # Scaled to species-specific central
estimates#(v2.0)
lnPeffDCA   = 0.0;      # Scaled to species-specific central
estimates#(v2.0)

# Oral Absorption rates
lnkTSD     = 0.336;
lnkAS = 0.336;
lnkTD = -2.303;
lnkAD = -0.288;
lnkASTCA    = -0.288;
lnkASTCOH   = -0.288;

# Background rates
lnTCABgd = -30;
lnDCABgd = -30;
```

```
# TCE Metabolism
lnVMaxC     = 0.0;      # Scaled by liver weight and species-specific 
central estimates
lnKMC = 0.0;      # Scaled to species-specific central estimates
lnClC = 0.0;      # Scaled to species-specific central estimates
lnFracOtherC     = 0.0;      # Ratio of DCA to non-DCA
lnFracTCAC  = 0.0;      # Ratio of TCA to TCOH
lnVMaxDCVGC = 0.0;      # Scaled by liver weight and species-specific 
central estimates
lnClDCVGC   = 0.0;      # Scaled to species-specific central estimates
lnKMDCVGC   = 0.0;      # Scaled to species-specific central estimates
lnVMaxKidDCVGC   = 0.0;      # Scaled by kidney weight and species-
specific central estimates
lnClKidDCVGC     = 0.0;      # Scaled to species-specific central 
estimates
lnKMKidDCVGC     = 0.0;      # Scaled to species-specific central 
estimates
#(v1.2) lnClClaraC     = 0.0;      # Scaled by BW^0.75
lnVMaxLungLivC   = 0.0;      #(v1.2) Ratio of lung Vmax to liver Vmax,
                  # Scaled to species-specific central estimates
lnKMClara   = 0.0;      #(v1.2) now in units of air concentration

# Inter-strain scaling - relative to B6C3F1
lnISOx = 0.0; # P450 oxidative metabolism
lnISTCA = 0.0; # fraction to TCA
lnISDCA = 0.0; # fraction to DCA
lnISConj = 0.0; # GSH conjugation metabolism
lnISkTCA = 0.0; # TCA metabolism/clearance
lnISkDCA = 0.0; # DCA metabolism/clearance
lnISkDCVG = 0.0; # DCVG -> DCVC
lnISkDCVC = 0.0; # DCVC clearance

# Clearance in lung
#(v1.2) lnVMaxClearC   = 0.0;      # Scaled by BW^0.75
#(v1.2) lnKMClear= 0.0;      #
lnFracLungSysC   = 0.0;      #(v1.2) ratio of systemic to local clearance 
of lung oxidation

# TCOH Metabolism
lnVMaxTCOHC = 0.0;      # Scaled by BW^0.75
lnClTCOHC   = 0.0;      # Scaled by BW^0.75
lnKMTCOH    = 0.0;      #
lnVMaxGlucC = 0.0;      # Scaled by BW^0.75
lnClGlucC   = 0.0;      # Scaled by BW^0.75
lnKMGluc    = 0.0;      #
lnkMetTCOHC = 0.0;      # Scaled by BW^-0.25

# DCA metabolism/clearance#(v2.0)
lnkClearDCAC     = 0;  # Scaled by BW^-0.25#(v2.0)
lnkDCAcen_perC   = 0;  # Scaled by BW^-0.25#(v2.0)
lnkDCAper_cenC   = 0;  # Scaled by BW^-0.25#(v2.0)

# TCA Metabolism/clearance
```

```
lnkUrnTCAC = 0.0;      # Scaled by (plasma volume)^-1 and species-
specific central estimates
lnkMetTCAC = 0.0;      # Scaled by BW^-0.25

# TCOG excretion and reabsorption
lnkBileC   = 0.0;      # Scaled by BW^-0.25
lnkEHRC    = 0.0;      # Scaled by BW^-0.25
lnkUrnTCOGC= 0.0;      # Scaled by (blood volume)^-1 and species-specific
central estimates

# DCVG metabolism
lnFracKidDCVCC   = 0.0;      # Ratio of "directly" to DCVC to systemic
DCVG
lnkDCVGC   = 0.0;      # Scaled by BW^-0.25

# DCVC metabolism
lnkElimDCVCC     = 0.0;      # Scaled by BW^-0.25#(v2.0)

# Closed chamber parameters
NRodents   = 1;  #
VChC  = 1;  #
lnkLossC   = 0;  #

#*********************************************************************
******
# Population means
#
# These are given truncated normal or uniform distributions, depending on
#     what prior information is available.  Note that these distributions
#     reflect uncertainty in the population mean, not inter-individual
#     variability.  Normal distributions are truncated at 2, 3, or 4 SD.
#           For fractional volumes and flows, 2xSD
#           For plasma fraction, 3xSD
#           For cardiac output and ventilation-perfusion ratio, 4xSD
#           For all others, 3xSD
#     For uniform distributions, range of 1e2 to 1e8 fold, centered on
#           central estimate.
#
M_lnQCC     = 1.0;
M_lnVPRC    = 1.0;
M_QFatC     = 1.0;
M_QGutC     = 1.0;
M_QLivC     = 1.0;
M_QSlwC     = 1.0;
#(v1.2) M_QTBC   = 1.0;
M_QKidC     = 1.0;
M_FracPlasC= 1.0;
M_lnDRespC = 1.0; #(v1.2)
M_VFatC     = 1.0;
M_VGutC     = 1.0;
M_VLivC     = 1.0;
M_VRapC     = 1.0;
#(v1.2) M_VTBC   = 1.0;
M_VRespLumC = 1.0; #(v1.2)
```

```
M_VRespEffC = 1.0; #(v1.2)
M_VKidC     = 1.0;
M_VBldC     = 1.0;
M_lnPBC     = 1.0;
M_lnPFatC   = 1.0;
M_lnPGutC   = 1.0;
M_lnPLivC   = 1.0;
M_lnPRapC   = 1.0;
#(v1.2) M_lnPTBC = 1.0;
M_lnPRespC  = 1.0; #(v1.2)
M_lnPKidC   = 1.0;
M_lnPSlwC   = 1.0;
M_lnPRBCPlasTCAC = 1.0;
M_lnPBodTCAC     = 1.0;
M_lnPLivTCAC     = 1.0;
M_lnkDissocC     = 1.0;
M_lnBMaxkDC = 1.0;
M_lnPBodTCOHC    = 1.0;
M_lnPLivTCOHC    = 1.0;
M_lnPBodTCOGC    = 1.0;
M_lnPLivTCOGC    = 1.0;
M_lnPeffDCVG     = 1.0;
M_lnPeffDCVC     = 1.0;#(v2.0)
M_lnPeffDCA = 1.0;#(v2.0)
M_lnkTSD    = 1.0;
M_lnkAS     = 1.0;
M_lnkTD     = 1.0;
M_lnkAD     = 1.0;
M_lnkASTCA  = 1.0;
M_lnkASTCOH = 1.0;
M_lnVMaxC   = 1.0;
M_lnKMC     = 1.0;
M_lnClC     = 1.0;
M_lnFracOtherC   = 1.0;
M_lnFracTCAC     = 1.0;
M_lnVMaxDCVGC    = 1.0;
M_lnClDCVGC = 1.0;
M_lnKMDCVGC = 1.0;
M_lnVMaxKidDCVGC = 1.0;
M_lnClKidDCVGC   = 1.0;
M_lnKMKidDCVGC   = 1.0;
#(v1.2) M_lnClClaraC   = 1.0;
M_lnVMaxLungLivC = 1.0; #(v1.2)
M_lnKMClara = 1.0;

# inter-strain scaling
M_lnISOx = 0.0; # P450 oxidative metabolism
M_lnISTCA = 0.0; # fraction to TCA
M_lnISDCA = 0.0; # fraction to DCA
M_lnISConj = 0.0; # GSH conjugation metabolism
M_lnISkTCA = 0.0; # TCA metabolism/clearance
M_lnISkDCA = 0.0; # DCA metabolism/clearance
M_lnISkDCVG = 0.0; # DCVG -> DCVC
M_lnISkDCVC = 0.0; # DCVC clearance
```

```
#(v1.2) M_lnVMaxClearC = 1.0;
#(v1.2) M_lnKMClear    = 1.0;
M_lnFracLungSysC = 1.0;
M_lnVMaxTCOHC    = 1.0;
M_lnClTCOHC = 1.0;
M_lnKMTCOH  = 1.0;
M_lnVMaxGlucC    = 1.0;
M_lnClGlucC = 1.0;
M_lnKMGluc  = 1.0;
M_lnkMetTCOHC    = 1.0;
M_lnkUrnTCAC     = 1.0;
M_lnkMetTCAC     = 1.0;
M_lnkBileC  = 1.0;
M_lnkEHRC   = 1.0;
M_lnkUrnTCOGC    = 1.0;
M_lnFracKidDCVCC = 1.0;
M_lnkDCVGC  = 1.0;
M_lnkElimDCVCC   = 1.0;#(v2.0)
M_lnkClearDCAC   = 1.0;#(v2.0)
M_lnkDCAcen_perC = 1.0;#(v2.0)
M_lnkDCAper_cenC = 1.0;#(v2.0)

#*********************************************************************
******
# Population Variances
#
# These are given InvGamma(alpha,beta) distributions.  The
parameterization
#     for alpha and beta is given by:
#           alpha = (n-1)/2
#           beta = s^2*(n-1)/2
#     where n = number of data points, and s^2 is the sample variance
#     Sum(x_i^2)/n - <x>^2.
# Generally, for parameters for which there is no direct data, assume a
#     value of n = 5 (alpha = 2).  For a sample variance s^2, this gives
#     an expected value for the standard deviation <sigma> = 0.9*s,
#     a median [2.5%,97.5%] of 1.1*s [0.6*s,2.9*s].
#
V_lnQCC     = 1.0;
V_lnVPRC    = 1.0;
V_QFatC     = 1.0;
V_QGutC     = 1.0;
V_QLivC     = 1.0;
V_QSlwC     = 1.0;
#(v1.2) V_QTBC   = 1.0;
V_QKidC     = 1.0;
V_FracPlasC = 1.0;
V_lnDRespC = 1.0; #(v1.2)
V_VFatC     = 1.0;
V_VGutC     = 1.0;
V_VLivC     = 1.0;
V_VRapC     = 1.0;
#(v1.2) V_VTBC   = 1.0;
```

```
V_VRespLumC = 1.0; #(v1.2)
V_VRespEffC = 1.0; #(v1.2)
V_VKidC     = 1.0;
V_VBldC     = 1.0;
V_lnPBC     = 1.0;
V_lnPFatC   = 1.0;
V_lnPGutC   = 1.0;
V_lnPLivC   = 1.0;
V_lnPRapC   = 1.0;
#(v1.2) V_lnPTBC = 1.0;
V_lnPRespC  = 1.0; #(v1.2)
V_lnPKidC   = 1.0;
V_lnPSlwC   = 1.0;
V_lnPRBCPlasTCAC = 1.0;
V_lnPBodTCAC     = 1.0;
V_lnPLivTCAC     = 1.0;
V_lnkDissocC     = 1.0;
V_lnBMaxkDC = 1.0;
V_lnPBodTCOHC    = 1.0;
V_lnPLivTCOHC    = 1.0;
V_lnPBodTCOGC    = 1.0;
V_lnPLivTCOGC    = 1.0;
V_lnPeffDCVG     = 1.0;
V_lnPeffDCVC     = 1.0;#(v2.0)
V_lnPeffDCA = 1.0;#(v2.0)
V_lnkTSD    = 1.0;
V_lnkAS     = 1.0;
V_lnkTD     = 1.0;
V_lnkAD     = 1.0;
V_lnkASTCA  = 1.0;
V_lnkASTCOH = 1.0;
V_lnVMaxC   = 1.0;
V_lnKMC     = 1.0;
V_lnClC     = 1.0;
V_lnFracOtherC   = 1.0;
V_lnFracTCAC     = 1.0;
V_lnVMaxDCVGC    = 1.0;
V_lnClDCVGC = 1.0;
V_lnKMDCVGC = 1.0;
V_lnVMaxKidDCVGC = 1.0;
V_lnClKidDCVGC   = 1.0;
V_lnKMKidDCVGC   = 1.0;
#(v1.2) V_lnClClaraC   = 1.0;
V_lnVMaxLungLivC = 1.0; #(v1.2)
V_lnKMClara = 1.0;

# inter-strain scaling
V_lnISOx = 1.0; # P450 oxidative metabolism
V_lnISTCA = 1.0; # fraction to TCA
V_lnISDCA = 1.0; # fraction to DCA
V_lnISConj = 1.0; # GSH conjugation metabolism
V_lnISkTCA = 1.0; # TCA metabolism/clearance
V_lnISkDCA = 1.0; # DCA metabolism/clearance
V_lnISkDCVG = 1.0; # DCVG -> DCVC
```

```
V_lnISkDCVC = 1.0; # DCVC clearance

#(v1.2) V_lnVMaxClearC = 1.0;
#(v1.2) V_lnKMClear    = 1.0;
V_lnFracLungSysC = 1.0;
V_lnVMaxTCOHC    = 1.0;
V_lnClTCOHC = 1.0;
V_lnKMTCOH  = 1.0;
V_lnVMaxGlucC    = 1.0;
V_lnClGlucC = 1.0;
V_lnKMGluc  = 1.0;
V_lnkMetTCOHC    = 1.0;
V_lnkUrnTCAC     = 1.0;
V_lnkMetTCAC     = 1.0;
V_lnkBileC  = 1.0;
V_lnkEHRC   = 1.0;
V_lnkUrnTCOGC    = 1.0;
V_lnFracKidDCVCC = 1.0;
V_lnkDCVGC  = 1.0;
V_lnkElimDCVCC   = 1.0;#(v2.0)
V_lnkClearDCAC   = 1.0;#(v2.0)
V_lnkDCAcen_perC = 1.0;#(v2.0)
V_lnkDCAper_cenC = 1.0;#(v2.0)


#***********************************************************************
******
# Measurement error variances for output

#(v1.2) Ve_AlvRet= 1;

Ve_RetDose  = 1;
Ve_CAlv     = 1;
Ve_CAlvPPM  = 1;
Ve_CInhPPM  = 1;
Ve_CInh     = 1;
Ve_CMixExh  = 1;
Ve_CArt     = 1;
Ve_CVen     = 1;
Ve_CBldMix  = 1;

Ve_CFat     = 1;
Ve_CGut     = 1;
Ve_CRap     = 1;
Ve_CSlw     = 1;
#(v1.2) Ve_CTB   = 1;
Ve_CHrt     = 1;
Ve_CKid     = 1;
Ve_CLiv     = 1;
Ve_CLung    = 1;
Ve_CMus     = 1;
Ve_CSpl     = 1;
Ve_CBrn     = 1;
Ve_zAExh    = 1;
```

```
Ve_zAExhpost     = 1;


Ve_CTCOH    = 1;
Ve_CKidTCOH = 1;
Ve_CLivTCOH = 1;
Ve_CLungTCOH     = 1;


Ve_CPlasTCA = 1;
Ve_CBldTCA  = 1;
Ve_CBodTCA  = 1;
Ve_CKidTCA  = 1;
Ve_CLivTCA  = 1;
Ve_CLungTCA = 1;
Ve_zAUrnTCA = 1;
Ve_zAUrnTCA_collect    = 1;
Ve_zAUrnTCA_sat  = 1;


Ve_zABileTCOG    = 1;
Ve_CTCOG    = 1;
Ve_CTCOGTCOH     = 1;
Ve_CKidTCOGTCOH  = 1;
Ve_CLivTCOGTCOH  = 1;
Ve_CLungTCOGTCOH = 1;
Ve_AUrnTCOGTCOH  = 1;
Ve_AUrnTCOGTCOH_collect      = 1;

Ve_AUrnTCOGTCOH_sat    = 1;


Ve_CDCVGmol = 1;#(v2.0)
Ve_CDCVCmol = 1;#(v2.0)
Ve_CDCAmol  = 1;#(v2.0)
Ve_AUrnTCTotMole = 1;
Ve_TotCTCOH = 1;
Ve_QPsamp   = 1;

#***********************************************************************
******
#***                 Defaults for input parameters
***
#***********************************************************************
******
##-- TCE dosing
      Conc = 0.0; # Inhalation exposure conc. (ppm)
      IVDose = 0.0;     # IV dose (mg/kg)
      PDose = 0.0;      # Oral gavage dose (mg/kg)
      Drink = 0.0;      # Drinking water dose (mg/kg/day)
      IADose = 0.0;     # Intraarterial dose (mg/kg)
      PVDose = 0.0;     # Portal vein dose (mg/kg)
##-- TCA dosing
      IVDoseTCA = 0.0;# IV dose (mg/kg) of TCA
```

```
      PODoseTCA = 0.0;# Oral dose (mg/kg) of TCA
##-- TCOH dosing
      IVDoseTCOH = 0.0;# IV dose (mg/kg) of TCOH
      PODoseTCOH = 0.0;# Oral dose (mg/kg) of TCOH
##-- Potentially time-varying parameters
      QPmeas = 0.0;     # Measured value of Alveolar ventilation QP
      TCAUrnSat = 0.0;# Flag for saturated TCA urine
      TCOGUrnSat = 0.0;# Flag for saturated TCOG urine
      UrnMissing = 0.0;# Flag for missing urine collection times

Initialize {

#************************************************************************
******
#***                  Parameter Initialization and Scaling
***
#************************************************************************
******
# Model Parameters (used in dynamics):
#     QC          Cardiac output (L/hr)
#     VPR         Ventilation-perfusion ratio
#     QPsamp            Alveolar ventilation (L/hr)
#     QFatCtmp    Scaled fat blood flow
#     QGutCtmp    Scaled gut blood flow
#     QLivCtmp    Scaled liver blood flow
#     QSlwCtmp    Scaled slowly perfused blood flow
#(v1.2)     QTBCtmp     Scaled tracheo-bronchial blood flow
#     DResptmp    Respiratory lumen:tissue diffusive clearance rate
#     QKidCtmp    Scaled kidney blood flow
#     FracPlas    Fraction of blood that is plasma (1-hematocrit)
#     VFat        Fat compartment volume (L)
#     VGut        Gut compartment volume (L)
#     VLiv        Liver compartment volume (L)
#     VRap        Rapidly perfused compartment volume (L)
#(v1.2)     VTB         Tracheo-bronchial compartment volume (L)
#     VRespLum    Volume of respiratory lumen (L air)
#     VRespEff    Effective volume of respiratory tissue (L air)
#     VKid        Kidney compartment volume (L)
#     VBld        Blood compartment volume (L)
#     VSlw        Slowly perfused compartment volume (L)
#     VPlas       Plasma compartment volume [fraction of blood] (L)
#     VBod        TCA Body compartment volume [not incl. blood+liver] (L)
#     VBodTCOH    TCOH/G Body compartment volume [not incl. liver] (L)
#     PB          TCE Blood/air partition coefficient
#     PFat        TCE Fat/Blood partition coefficient
#     PGut        TCE Gut/Blood partition coefficient
#     PLiv        TCE Liver/Blood partition coefficient
#     PRap        TCE Rapidly perfused/Blood partition coefficient
#(v1.2)     PTB         TCE Tracheo-bronchial/Blood partition coefficient
#     PResp       TCE Respiratory tissue:air partition coefficient
#     PKid        TCE Kidney/Blood partition coefficient
#     PSlw        TCE Slowly perfused/Blood partition coefficient
#     TCAPlas           TCA blood/plasma concentration ratio
#     PBodTCA           Free TCA Body/blood plasma partition coefficient
```

```
#     PLivTCA           Free TCA Liver/blood plasma partition coefficient
#     kDissoc           Protein/TCA dissociation constant (umole/L)
#     BMax       Maximum binding concentration (umole/L)
#     PBodTCOH   TCOH body/blood partition coefficient
#     PLivTCOH   TCOH liver/body partition coefficient
#     PBodTCOG   TCOG body/blood partition coefficient
#     PLivTCOG   TCOG liver/body partition coefficient
#     kAS        TCE Stomach absorption coefficient (/hr)
#     kTSD       TCE Stomach-duodenum transfer coefficient (/hr)
#     kAD        TCE Duodenum absorption coefficient (/hr)
#     kTD        TCE Duodenum-feces transfer coefficient (/hr)
#     kASTCA            TCA Stomach absorption coefficient (/hr)
#     kASTCOH           TCOH Stomach absorption coefficient (/hr)
#     VMax       VMax for hepatic TCE oxidation (mg/hr)
#     KM         KM for hepatic TCE oxidation (mg/L)
#     FracOther  Fraction of hepatic TCE oxidation not to TCA+TCOH
#     FracTCA           Fraction of hepatic TCE oxidation to TCA
#     VMaxDCVG   VMax for hepatic TCE GSH conjugation (mg/hr)
#     KMDCVG            KM for hepatic TCE GSH conjugation (mg/L)
#     VMaxKidDCVG VMax for renal TCE GSH conjugation (mg/hr)
#     KMKidDCVG  KM for renal TCE GSH conjugation (mg/L)
#     VMaxClara  VMax for Tracheo-bronchial TCE oxidation (mg/hr)
#     KMClara           KM for Tracheo-bronchial TCE oxidation (mg/L)
#(v1.2)     VMaxClear  VMax for Tracheo-bronchial CH clearance (mg/hr)
#(v1.2)     KMClear           KM for Tracheo-bronchial CH clearance (mg/L)
#     FracLungSys Fraction of respiratory metabolism to systemic circ.
#     VMaxTCOH   VMax for hepatic TCOH->TCA (mg/hr)
#     KMTCOH            KM for hepatic TCOH->TCA (mg/L)
#     VMaxGluc   VMax for hepatic TCOH->TCOG (mg/hr)
#     KMGluc            KM for hepatic TCOH->TCOG (mg/L)
#     kMetTCOH   Rate constant for hepatic TCOH->other (/hr)
#     kUrnTCA           Rate constant for TCA plasma->urine (/hr)
#     kMetTCA           Rate constant for hepatic TCA->other (/hr)
#     kBile      Rate constant for TCOG liver->bile (/hr)
#     kEHR       Lumped rate constant for TCOG bile->TCOH liver (/hr)
#     kUrnTCOG   Rate constant for TCOG->urine (/hr)
#     kDCVG      Rate constant for hepatic DCVG->DCVC (/hr)
#     FracKidDCVC Fraction of renal TCE GSH conj. "directly" to DCVC
#                (i.e., via first pass)
#     VDCVG      DCVG effective volume of distribution
#     VDCVC      DCVC effective volume of distribution#(v2.0)
#     VDCA       DCA effective volume of distribution#(v2.0)
#     kElimDCVC  Lumped rate constant for DCVC elimination (/hr)#(v2.0)
#           kClearDCA  Rate constant for DCA elimination (/hr)#(v2.0)
#     kDCAcen_per Rate constant for DCA central->peripheral (/hr)#(v2.0)
#     kDCAper_cen Rate constant for DCA peripheral->central (/hr)#(v2.0)
#     Rodents           Number of rodents in closed chamber data
#     VCh        Chamber volume for closed chamber data
#     kLoss      Rate constant for closed chamber air loss
# Parameters used (not assigned here)
#     BW         Body weight in kg
#     Species           1 = human (default), 2 = rat, 3 = mouse
#     Male       0 = female, 1 (default) = male
#     CC         Closed chamber initial concentration
```

```
# Sampling/scaling parameters (assigned or sampled)
#     lnQCC
#     lnVPRC
#     lnDRespC
#     QFatC
#     QGutC
#     QLivC
#     QSlwC
#(v1.2)     QTBC
#     QKidC
#     FracPlasC
#     VFatC
#     VGutC
#     VLivC
#     VRapC
#(v1.2)     VTBC
#     VRespLumC
#     VRespEffC
#     VKidC
#     VBldC
#     lnPBC
#     lnPFatC
#     lnPGutC
#     lnPLivC
#     lnPRapC
#     lnPSlwC
#(v1.2)     lnPTBC
#     lnPRespC
#     lnPKidC
#     lnPRBCPlasTCAC
#     lnPBodTCAC
#     lnPLivTCAC
#     lnkDissocC
#     lnBMaxkDC
#     lnPBodTCOHC
#     lnPLivTCOHC
#     lnPBodTCOGC
#     lnPLivTCOGC
#     lnPeffDCVG
#     lnPeffDCVC#(v2.0)
#     lnPeffDCA#(v2.0)
#     lnkTSD
#     lnkAS
#     lnkTD
#     lnkAD
#     lnkASTCA
#     lnkASTCOH
#     lnVMaxC
#     lnKMC
#     lnClC
#     lnFracOtherC
#     lnFracTCAC
#     lnVMaxDCVGC
#     lnClDCVGC
```

```
#     lnKMDCVGC
#     lnVMaxKidDCVGC
#     lnClKidDCVGC
#     lnKMKidDCVGC
#(v1.2)     lnClClaraC
#     lnVMaxLungLivC
#     lnKMClara
#(v1.2)     lnVMaxClearC
#(v1.2)     lnKMClear
#     lnFracLungSysC
#     lnVMaxTCOHC
#     lnClTCOHC
#     lnKMTCOH
#     lnVMaxGlucC
#     lnClGlucC
#     lnKMGluc
#     lnkMetTCOHC
#     lnkUrnTCAC
#     lnkMetTCAC
#     lnkBileC
#     lnkEHRC
#     lnkUrnTCOGC
#     lnFracKidDCVCC
#     lnkDCVGC
#     lnkElimDCVCC#(v2.0)
#     NRodents
#     VChC
#     lnkLossC
# Input parameters
#     none
# Notes:
#*************************************************************************
******
      # use measured value of > 0, otherwise use 0.03 for mouse,
      #     0.3 for rat, 60 for female human, 70 for male human
      BW = (BWmeas > 0.0 ? BWmeas : (Species == 3 ? 0.03 : (Species == 2
? 0.3 : (Male == 0 ? 60.0 : 70.0) )));

      BW75 = pow(BW, 0.75);
      BW25 = pow(BW, 0.25);

# Cardiac Output and alveolar ventilation (L/hr)
      QC = exp(lnQCC) * BW75 *     # Mouse, Rat, Human (default)
            (Species == 3 ? 11.6 : (Species == 2 ? 13.3 : 16.0 ));
      # Mouse: CO=13.98 +/- 2.85 ml/min, BW=30 g (Brown et al. 1997, Tab.
22)
      #     Uncertainty CV is 0.20
      # Rat: CO=110.4 ml/min +/- 15.6, BW=396 g (Brown et al. 1997, Tab.
22,
      #     p 441).  Uncertainty CV is 0.14.
      # Human: Average of Male CO=6.5 l/min, BW=73 kg
      #     and female CO= 5.9 l/min, BW=60 kg (ICRP #89, sitting at
rest)
```

```
      #     From Price et al. 2003, estimates of human perfusion rate
were
      #     4.7~6.5 for females and 5.5~7.1 l/min for males (note
      #     portal blood was double-counted, and subtracted off here)
      #     Thus for uncertainty use CV of 0.2, truncated at 4xCV
      #     Variability from Price et al. (2003) had CV of 0.14~0.20,
      #     so use 0.2 as central estimate
      VPR = exp(lnVPRC)*
            (Species == 3 ? 2.5 : (Species == 2 ? 1.9 : 0.96 ));
      # Mouse: QP/BW=116.5 ml/min/100 g (Brown et al. 1997, Tab. 31),
VPR=2.5
      #     Assume uncertainty CV of 0.2 similar to QC, truncated at 4xCV
      #     Consistent with range of QP in Tab. 31
      # Rat: QP/BW=52.9 ml/min/100 g (Brown et al. 1997, Tab. 31),
VPR=1.9
      #     Assume uncertainty CV of 0.3 similar to QC, truncated at 4xCV
      #     Used larger CV because Tab. 31 shows a very large range of QP
      # Human: Average of Male VE=9 l/min, resp. rate=12 /min,
      #     dead space=0.15 l (QP=7.2 l/min), and Female
      #     VE=6.5 l/min, resp. rate=14 /min, dead space=0.12 l
      #     (QP=4.8 l/min), VPR = 0.96
      #     Assume uncertainty CV of 0.2 similar to QC, truncated at 4xCV
      #     Consistent with range of QP in Tab. 31
      QPsamp = QC*VPR;

#(v1.2) Respiratory diffusion flow rate
#     Will be scaled by QP in dynamics
#     Use log-uniform distribution from 1e-5 to 10
      DResptmp = exp(lnDRespC);

# Fractional Flows scaled to the appropriate species
# Fat = Adipose only
# Gut = GI tract + pancreas + spleen (all drain to portal vein)
# Liv = Liver, hepatic artery
# Slw = Muscle + Skin
# TB = Bronchial blood flow  #(v1.2) -- deleted
# Kid = Kidney
# Rap = Rapidly perfused (rest of organs, plus bone marrow, lymph, etc.),
#     derived by difference in dynamics
#
# Mouse and rat data from Brown et al. (1997).  Human data from
#     ICRP-89 (2002), and is sex-specific.

      QFatCtmp = QFatC*
      (Species == 3 ? 0.07 : (Species == 2 ? 0.07 : (Male == 0 ? 0.085 :
0.05) ));
      QGutCtmp = QGutC*
      (Species == 3 ? 0.141 : (Species == 2 ? 0.153 : (Male == 0 ? 0.21 :
0.19) ));
      QLivCtmp = QLivC*
      (Species == 3 ? 0.02 : (Species == 2 ? 0.021 : 0.065 ));
      QSlwCtmp = QSlwC*
      (Species == 3 ? 0.217 : (Species == 2 ? 0.336 : (Male == 0 ? 0.17 :
0.22) ));
```

```
#(v1.2)     QTBCtmp = QTBC*
#(v1.2)           (Species == 3 ? 0.005 : (Species == 2 ? 0.021 : 0.025
));
      QKidCtmp = QKidC*
            (Species == 3 ? 0.091 : (Species == 2 ? 0.141 : (Male == 0 ?
0.17 : 0.19) ));

# Plasma Flows to Tissues (L/hr)
## Mice and rats from Hejtmancik et al. 2002,
##    control F344 rats and B6C3F1 mice at 19 weeks of age
## However, there appear to be significant strain differences in rodents,
so
##    assume uncertainty CV=0.2 and variability CV=0.2.
## Human central estimate from ICRP.  Well measured in humans, from Price
et al.,
##    human SD in hematocrit was 0.029 in females, 0.027 in males,
##    corresponding to FracPlas CV of 0.047 in females and
##    0.048 in males.  Use rounded CV = 0.05 for both uncertainty and
variability
## Use measured 1-hematocrit if available
## Truncate distributions at 3xCV to encompass clinical "normal range"
      FracPlas = (Hematocritmeas > 0.0 ? (1-Hematocritmeas) : (FracPlasC
*
      (Species == 3 ? 0.52 : (Species == 2 ? 0.53 : (Male == 0 ? 0.615 :
0.567)))));

# Tissue Volumes (L)
# Fat = Adipose only
# Gut = GI tract (not contents) + pancreas + spleen (all drain to portal
vein)
# Liv = Liver
# Rap = Brain + Heart + (Lungs-TB) + Bone marrow + "Rest of the body"
# TB = Tracheobroncial region (trachea+broncial basal+
#           broncial secretory+bronchiolar) #(v1.2) replaced by VResp
# Kid = Kidney
# Bld = Blood
# Slw = Muscle + Skin, derived by difference
# residual (assumed unperfused) = (Bone-Marrow)+GI contents+other
#
# Mouse and rat data from Brown et al. (1997).  Human data from
#     ICRP-89 (2002), and is sex-specific.

        VFat = BW * (VFatCmeas > 0.0 ? VFatCmeas : (VFatC * (Species == 3
? 0.07 : (Species == 2 ? 0.07 : (Male == 0 ? 0.317 : 0.199)  ))));
        VGut = VGutC * BW *
      (Species == 3 ? 0.049 : (Species == 2 ? 0.032 : (Male == 0 ? 0.022
: 0.020) ));
        VLiv = VLivC * BW *
      (Species == 3 ? 0.055 : (Species == 2 ? 0.034 : (Male == 0 ? 0.023
: 0.025) ));
        VRap = VRapC * BW * ## VRaptmp = VRapC * BW * #(v1a)#(v1b)#(v1c)
-- temporary variable
      (Species == 3 ? 0.100 : (Species == 2 ? 0.088 : (Male == 0 ? 0.093
: 0.088) ));
```

```
#(v1.2)         VTB = VTBC * BW *
#(v1.2)     (Species == 3 ? 0.0007 : (Species == 2 ? 0.0005 : 0.00018 ));
      VRespLum = VRespLumC * BW *
      (Species == 3 ? (0.00014/0.03) : (Species == 2 ? (0.0014/0.3) :
(0.167/70) )); #(v1.2) Lumenal volumes from Styrene model (Sarangapani et
al. 2002)
      VRespEfftmp = VRespEffC * BW *
      (Species == 3 ? 0.0007 : (Species == 2 ? 0.0005 : 0.00018 ));
      #(v1.2) Respiratory tract volume is TB region
      #(v1.2) will be multiplied by partition coef. below
                 ## VRap = VRaptmp + VTB; #(v1b) -- add TB to rapidly
perfused
      VKid = VKidC * BW *
      (Species == 3 ? 0.017 : (Species == 2 ? 0.007 : (Male == 0 ? 0.0046
: 0.0043) ));
        VBld = VBldC * BW *
      (Species == 3 ? 0.049 : (Species == 2 ? 0.074 : (Male == 0 ? 0.068
: 0.077) ));
                 ## VRap = VRaptmp + VBld; #(v1a) -- add Bld to rapidly
perfused
                 ## VRap = VRaptmp + VTB + VBld;#(v1c) -- add TB and Bld
to Rap
#(v1.2)        VSlw = (Species == 3 ? 0.8897 : (Species == 2 ? 0.8995 :
(Male == 0 ? 0.85778 : 0.856))) * BW
#(v1.2)          - VFat - VGut - VLiv - VRap - VTB - VKid - VBld;
        VSlw = (Species == 3 ? 0.8897 : (Species == 2 ? 0.8995 : (Male ==
0 ? 0.85778 : 0.856))) * BW
           - VFat - VGut - VLiv - VRap - VRespEfftmp - VKid - VBld;
      #(v1.2) replaced VTB by VRespEfftmp

                 ## - VFat - VGut - VLiv - VRaptmp - VTB - VKid - VBld;
#(v1a)#(v1b)#(v1c)
# Slowly perfused:
# Baseline mouse: 0.8897-0.049-0.017-0.0007-0.1-0.055-0.049-0.07= 0.549
# Baseline rat: 0.8995 -0.074-0.007-0.0005-0.088-0.034-0.032-0.07= 0.594
# Baseline human F: 0.85778-0.068-0.0046-0.00018-0.093-0.023-0.022-0.317=
0.33
# Baseline human M: 0.856-0.077-0.0043-0.00018-0.088-0.025-0.02-0.199=
0.4425

       VPlas = FracPlas * VBld;
#(v1.2)     VBod = VFat + VGut + VRap + VTB + VKid + VSlw; # For TCA
      VBod = VFat + VGut + VRap + VRespEfftmp + VKid + VSlw; # For TCA
      ## VFat + VGut + VRap + VTB + VKid + VSlw + VBld;#(v1h)
                 ## VFat + VGut + VRaptmp + VTB + VKid + VSlw;
#(v1a)#(v1b)#(v1c) For TCA
      ## VFat + VGut + VRaptmp + VTB + VKid + VSlw +
VBld;#(v1a)(v1b)+(v1h)
      VBodTCOH = VBod + VBld;     # for TCOH and TCOG -- body without
liver
      ## VBodTCOH = VBod; #(v1h)

# Partition coefficients
```

```
    PB = (PBmeas > 0.0 ? PBmeas : (exp(lnPBC) * (Species == 3 ? 15. :
(Species == 2 ? 22. : 9.5 )))); # Blood-air
   # Mice: pooling Abbas and Fisher 1997, Fisher et al. 1991
   #     each a single measurement, with overall CV = 0.07.
   #     Given small number of measurements, and variability
   #     in rat, use CV of 0.25 for uncertainty and variability.
   # Rats: pooling Sato et al. 1977, Gargas et al. 1989,
   #     Barton et al. 1995, Simmons et al. 2002, Koizumi 1989,
   #     Fisher et al. 1989.  Fisher et al. measurement substantially
   #     smaller than others (15 vs. 21~26).  Recent article
   #     by Rodriguez et al. 2007 shows significant change with
   #     age (13.1 at PND10, 17.5 at adult, 21.8 at aged), also seems
   #     to favor lower values than previously reported.  Therefore
   #     use CV = 0.25 for uncertainty and variability.
   # Humans: pooling Sato and Nakajima 1979, Sato et al. 1977,
   #     Gargas et al. 1989, Fiserova-Bergerova et al. 1984,
   #     Fisher et al. 1998, Koizumi 1989
   #     Overall variability CV = 0.185.  Consistent with
   #     within study inter-individual variability CV = 0.07~0.22.
   #     Study-to-study, sex-specific means range 8.1~11, so
   #     uncertainty CV = 0.2.
    PFat = exp(lnPFatC) *      # Fat/blood
         (Species == 3 ? 36. : (Species == 2 ? 27. : 67. ));
   # Mice: Abbas and Fisher 1997.  Single measurement.  Use
   #     rat uncertainty of CV = 0.3.
   # Rats: Pooling Barton et al. 1995, Sato et al. 1977,
   #     Fisher et al. 1989.  Recent article by Rodriguez et al.
   #     (2007) shows higher value of 36., so assume uncertainty
   #     CV of 0.3.
   # Humans: Pooling Fiserova-Bergerova et al. 1984, Fisher et al.
1998,
   #     Sato et al. 1977.  Variability in Fat:Air has CV = 0.07.
   #     For uncertainty, dominated by PB uncertainty CV = 0.2
   #     For variability, add CVs in quadrature for
   #     sqrt(0.07^2+0.185^2)=0.20
    PGut = exp(lnPGutC) *            # Gut/blood
         (Species == 3 ? 1.9 : (Species == 2 ? 1.4 : 2.6 ));
   # Mice: Geometric mean of liver, kidney
   # Rats: Geometric mean of liver, kidney
   # Humans: Geometric mean of liver, kidney
   #     Uncertainty of CV = 0.4 due to tissue extrapolation
    PLiv = exp(lnPLivC) *            # Liver/blood
         (Species == 3 ? 1.7 : (Species == 2 ? 1.5 : 4.1 ));
   # Mice: Fisher et al. 1991, single datum, so assumed uncert CV =
0.4
   # Rats: Pooling Barton et al. 1995, Sato et al. 1977,
   #     Fisher et al. 1989, with little variation (range 1.3~1.7).
   #     Recent article by Rodriguez et al.reports 1.34.  Use
   #     uncertainty CV = 0.15.
   # Humans: Pooling Fiserova-Bergerova et al. 1984, Fisher et al.
1998
   #     almost 2-fold difference in Liver:Air values, so uncertainty
   #     CV = 0.4
    PRap = exp(lnPRapC) *            # Rapidly perfused/blood
```

```
                  ## PRaptmp = exp(lnPRapC) * #(v1a)#(v1b)#(v1c) --
temporary
            (Species == 3 ? 1.9 : (Species == 2 ? 1.3 : 2.6 ));
      # Mice: Similar to liver, kidney.  Uncertainty CV = 0.4 due to
      #     tissue extrapolation
      # Rats: Use brain values Sato et al. 1977.  Recent article by
      #     Rodriguez et al. (2007) reports 0.99 for brain.  Uncertainty
      #     CV of 0.4 due to tissue extrapolation.
      # Humans: Use brain from Fiserova-Bergerova et al. 1984
      #     Uncertainty of CV = 0.4 due to tissue extrapolation
#(v1.2)       PTB = exp(lnPTBC) *       # TB/blood
#(v1.2)           (Species == 3 ? 2.6 : (Species == 2 ? 1.0 : 1.3 ));
       PResp = exp(lnPRespC) *          # Resp/blood =
            (Species == 3 ? 2.6 : (Species == 2 ? 1.0 : 1.3 ));
      # Mice: Abbas and Fisher 1997, single datum, so assumed uncert CV =
0.4
      # Rats: Sato et al. 1977, single datum, so assumed uncert CV = 0.4
      # Humans: Pooling Fiserova-Bergerova et al. 1984, Fisher et al.
1998
      #     > 2-fold difference in lung:air values, so uncertainty
      #     CV = 0.4
       VRespEff = VRespEfftmp * PResp * PB; #(v1.2) Effective air volume
                   ##     PRap = (PRaptmp*VRaptmp + VBld)/VRap; #(v1a)
                 ##         #(v1a) adjust PC for blood
                   ##     PRaptmp2 = (PRaptmp*VRaptmp + VBld)/VRap;
#(v1c)
                 ##         #(v1c) first adjust PC for blood
                 ##    QRapCtmp = 1 - QFatCtmp - QGutCtmp - QLivCtmp
#(v1b)#(v1c)
                 ##         - QSlwCtmp - QTBCtmp - QKidCtmp;
#(v1b)#(v1c)
                 ##     PRap = (QRapCtmp+QTBCtmp)/(QRapCtmp/PRaptmp
#(v1b)
                 ##         + QTBCtmp/PTB); #(v1b) -- adjust PC for TB
                 ##     PRap = (QRapCtmp+QTBCtmp)/(QRapCtmp/PRaptmp2
#(v1c)
                 ##         + QTBCtmp/PTB); #(v1c) -- then adjust PC for
TB
       PKid = exp(lnPKidC) *            # Slowly perfused/blood
            (Species == 3 ? 2.1 : (Species == 2 ? 1.3 : 1.6 ));
      # Mice: Abbas and Fisher 1997, single datum, so assumed uncert CV =
0.4
      # Rats: Pooling Barton et al. 1995, Sato et al. 1977.  Recent
article
      #     by Rodriguez et al. (2007) reports 1.01, so use uncertainty
      #     CV of 0.3.  Pooled variability CV = 0.39.
      # Humans: Pooling Fiserova-Bergerova et al. 1984, Fisher et al.
1998
      #     For uncertainty, dominated by PB uncertainty CV = 0.2
      #     Variability in kidney:air CV = 0.23, so add to PB variability
      #     in quadrature    sqrt(0.23^2+0.185^2)=0.30
       PSlw = exp(lnPSlwC) *            # Slowly perfused/blood
            (Species == 3 ? 2.4 : (Species == 2 ? 0.58 : 2.1 ));
      # Mice: Muscle - Abbas and Fisher 1997, single datum, so assumed
```

```
      #     uncert CV = 0.4
      # Rats: Pooling Barton et al. 1995, Sato et al. 1977,
      #     Fisher et al. 1989.  Recent article by Rodriguez et al.
(2007)
      #     reported 0.72, so use uncertainty CV of 0.25.  Variability
      #     in Muscle:air and muscle:blood ~ CV = 0.3
      # Humans: Pooling Fiserova-Bergerova et al. 1984, Fisher et al.
1998
      #     Range of values 1.4~2.4, so uncertainty CV = 0.3
      #     Variability in muscle:air CV = 0.3, so add to PB variability
      #     in quadrature sqrt(0.3^2+0.185^2)=0.35

# TCA partitioning
    TCAPlas = FracPlas + (1 - FracPlas) * 0.5 * exp(lnPRBCPlasTCAC);
      #     Blood/Plasma concentration ratio.  Note dependence
      #     on fraction of blood that is plasma.  Here
      #     exp(lnPRBCPlasTCA) = partition coefficient
      #           C(blood minus plasma)/C(plasma)
      #     Default of 0.5, corresponding to Blood/Plasma
      #           concentration ratio of 0.76 in
      #           rats (Schultz et al 1999)
      #     For rats, Normal uncertainty with GSD = 1.4
      #     For mice and humans, diffuse prior uncertainty of
      #     100-fold up/down
    PBodTCA = TCAPlas * exp(lnPBodTCAC) *
          (Species == 3 ? 0.88 : (Species == 2 ? 0.88 : 0.52 ));
      # Note -- these were done at 10~20 microg/ml (Abbas and Fisher
1997),
      #     which is 1.635-3.27 mmol/ml (1.635-3.27 x 10^6 microM).
      #     At this high concentration, plasma binding should be
      #     saturated -- e.g., plasma albumin concentration was
      #     measured to be P=190-239 microM in mouse, rat, and human
      #     plasma by Lumpkin et al. 2003, or > 6800 molecules of
      #     TCA per molecule of albumin.  So the measured partition
      #     coefficients should reflect free blood-tissue partitioning.
      # Used muscle values, multiplied by blood:plasma ratio to get
      #     Body:Plasma partition coefficient
      # Rats = mice from Abbas and Fisher 1997
      # Humans from Fisher et al. 1998
      #     Uncertainty in mice, humans GSD = 1.4
      #     For rats, GSD = 2.0, based on difference between mice
      #     and humans.
    PLivTCA = TCAPlas * exp(lnPLivTCAC) *
          (Species == 3 ? 1.18 : (Species == 2 ? 1.18 : 0.66 ));
      # Multiplied by blood:plasma ratio to get Liver:Plasma
      # Rats = mice from Abbas and Fisher 1997
      # Humans from Fisher et al. 1998
      #     Uncertainty in mice, humans GSD = 1.4
      #     For rats, GSD = 2.0, based on difference between mice
      #     and humans.

# Binding Parameters for TCA
      # GM of Lumpkin et al. 2003; Schultz et al. 1999;
      #     Templin et al. 1993, 1995; Yu et al. 2000
```

```
      # Protein/TCA dissociation constant (umole/L)
      #     note - GSD = 3.29, 1.84, and 1.062 for mouse, rat, human
      kDissoc = exp(lnkDissocC) *
            (Species == 3 ? 107. : (Species == 2 ? 275. : 182. ));
      # BMax = NSites * Protein concentration.  Sampled parameter is
      #     BMax/kD (determines binding at low concentrations)
      #     note - GSD = 1.64, 1.60, 1.20 for mouse, rat, human
      BMax = kDissoc * exp(lnBMaxkDC) *
            (Species == 3 ? 0.88 : (Species == 2 ? 1.22 : 4.62 ));

# TCOH partitioning
      # Data from Abbas and Fisher 1997 (mouse) and Fisher et al.
      #     1998 (human).  For rat, used mouse values.
      #     Uncertainty in mice, humans GSD = 1.4
      #     For rats, GSD = 2.0, based on difference between mice
      #     and humans.

    PBodTCOH = exp(lnPBodTCOHC) *
            (Species == 3 ? 1.11 : (Species == 2 ? 1.11 : 0.91 ));
    PLivTCOH = exp(lnPLivTCOHC) *
            (Species == 3 ? 1.3 : (Species == 2 ? 1.3 : 0.59 ));

# TCOG partitioning
      # Use TCOH as a proxy, but uncertainty much greater
      # (e.g., use uniform prior, 100-fold up/down)
    PBodTCOG = exp(lnPBodTCOGC) *
            (Species == 3 ? 1.11 : (Species == 2 ? 1.11 : 0.91 ));
    PLivTCOG = exp(lnPLivTCOGC) *
            (Species == 3 ? 1.3 : (Species == 2 ? 1.3 : 0.59 ));

# DCVG distribution volume
      # exp(lnPeffDCVG) is the effective partition coefficient for
      # the "body" (non-blood) compartment
      # Diffuse prior distribution: loguniform 1e-3 to 1e3
      VDCVG = VBld +   # blood plus body (with "effective" PC)
      exp(lnPeffDCVG) * (VBod + VLiv);

# DCVC distribution volume#(v2.0)
      # exp(lnPeffDCVC) is the effective partition coefficient for#(v2.0)
      # the "body" (non-blood) compartment#(v2.0)
      # Diffuse prior distribution: loguniform 1e-3 to 1e3#(v2.0)
      VDCVC = VBld +   # blood plus body (with "effective" PC)#(v2.0)
      exp(lnPeffDCVC) * (VBod + VLiv);#(v2.0)

# DCA distribution volume#(v2.0)
      # exp(lnPeffDCA) is the effective partition coefficient for#(v2.0)
      # the "body" (non-blood) compartment#(v2.0)
      # Diffuse prior distribution: loguniform 1e-3 to 1e3#(v2.0)
      VDCA = VBld +    # blood plus body (with "effective" PC)#(v2.0)
      exp(lnPeffDCA) * (VBod + VLiv);#(v2.0)

# Absorption Rate Constants (/hr)
      # All priors are diffuse (log)uniform distributions
```

```
      # transfer from stomach centered on 1.4/hr, range up or down 100-
fold,
      #     based on human stomach half-time of 0.5 hr.
        kTSD = exp(lnkTSD);
      # stomach absorption centered on 1.4/hr, range up or down 1000-fold
      kAS = exp(lnkAS);
      # assume no fecal excretion -- 100% absorption
      kTD = 0.0 * exp(lnkTD);
      # intestinal absorption centered on 0.75/hr, range up or down
      #     1000-fold, based on human transit time of small intestine
      #     of 4 hr    (95% throughput in 4 hr)
      kAD = exp(lnkAD);
      kASTCA = exp(lnkASTCA);
      kASTCOH = exp(lnkASTCOH);

# Background
      TCABgd = (lnTCABgd > -30 ? exp(lnTCABgd) : 0);
      DCABgd = (lnDCABgd > -30 ? exp(lnDCABgd) : 0);

# TCE Oxidative Metabolism Constants
# For rodents, in vitro microsomal data define priors (pooled).
# For human, combined in vitro microsomoal+hepatocellular individual data
#     define priors.
# All data from Elfarra et al. 1998; Lipscomb et al. 1997, 1998a,b
# For VMax, scaling from in vitro data were (Barter et al. 2007):
#     32 mg microsomal protein/g liver
#     99 x 1e6 hepatocytes/g liver
#     Here, human data assumed representative of mouse and rats.
# For KM, two different scaling methods were used for microsomes:
#     Assume microsomal concentration = liver concentration, and
#           use central estimate of liver:blood PC (see above)
#     Use measured microsome:air partition coefficient (1.78) and
#           central estimate of blood:air PC (see above)
# For human KM from hepatocytes, used measured human hepatocyte:air
#     partition coefficient (21.62, Lipscomb et al. 1998), and
#     central estimate of blood:air PC.
#     Note that to that the hepatocyte:air PC is similar to that
#     found in liver homogenates (human: 29.4+/-5.1 from Fiserova-
#     Bergerova et al. 1984, and 54 for Fisher et al. 1998; rat:
#     27.2+/-3.4 from Gargas et al. 1989, 62.7 from Koisumi 1989,
#     43.6 from Sato et al. 1977; mouse: 23.2 from Fisher et al. 1991).
# For humans, sampled parameters are VMax and ClC (VMax/KM), due to
#     improved convergence.  VMax is kept as a parameter because it
#     appears less uncertain (i.e., more consistent across microsomal
#     and hepatocyte data).

      # Central estimate of VMax is 342, 76.2, and 32.3 (micromol/min/
      #     kg liver) for mouse, rat, human.  Converting to /hr by
      #     * (60 min/hr * 0.1314 mg/micromol) gives
      #     2700, 600, and 255 mg/hr/kg liver
      # Observed variability of about 2-fold GSD.  Assume 2-fold GSD for
      #     both uncertainty and variability
        VMax = exp(lnISOx) * VLiv*exp(lnVMaxC)* # apply interstrain
scaling
```

```
            (Species == 3 ? 2700. : (Species == 2 ? 600. : 255.));

      # For mouse and rat central estimates for KM are 0.068~1.088 and
      #     0.039~0.679 mmol/l in blood, depending on the scaling
      #     method used.  Taking the geometric mean, and converting
      #     to mg/l by 131.4 mg/mmol gives 36. and 21. mg/l in blood.
      # For human, central estimate
      #     for Cl are 0.306~3.95 l/min/kg liver.  Taking the geometric
      #     mean and converting to /hr gives a central estimate of
      #     66. l/hr/kg.
      #     KM is then derived from KM = VMax/(Cl*Vliv) (central estimate
      #     of
      # Note uncertainty due to scaling is about 4-fold.
      #     Variability is about 3-fold in mice, 1.3-fold in rats, and
      #     2- to 4- fold in humans (depending on scaling).
        KM = (Species == 3 ? 36.*exp(lnKMC) : (Species == 2 ?
21.*exp(lnKMC) : VMax/(VLiv*66.*exp(lnClC))));

# Oxidative metabolism splits
      # Fractional split of TCE to DCA
      # exp(lnFracOtherC) = ratio of DCA to non-DCA
      # Diffuse prior distribution: loguniform 1e-4 to 1e2
      FracOther = exp(lnISDCA) * exp(lnFracOtherC)/(1+exp(lnFracOtherC));
              # apply interstrain scaling
      # Fractional split of TCE to TCA
      # exp(lnFracTCAC) = ratio of TCA to TCOH
      # TCA/TCOH = 0.1 from Lipscomb et al. 1998 using fresh hepatocytes,
      # but TCA/TCOH ~ 1 from Bronley-DeLancey et al 2006
      # GM = 0.32, GSD = 3.2
      FracTCA = 0.32*exp(lnISTCA+lnFracTCAC)*(1-FracOther)/
            (1+0.32*exp(lnISTCA+lnFracTCAC));
              # apply interstrain scaling

# TCE GSH Metabolism Constants
# Human in vitro data from Lash et al. 1999, define human priors.
#                VMax (nmol/min/  KM (mM)          CLeff (ml/min/
#                      g tissue)                  g tissue)
#                      ---------------------------------------------
#                [high affinity pathway only] [total]
# Human liver cytosol:      ~423       0.0055~0.023     21.2~87.0
# Human liver cytosol+ ~211       --          --
#     microsomes
#                [total]          [total]          [total]
# Human hepatocytes*   12~30**          0.012~0.039***   0.2~0.5****
# Human kidney cytosol:     81         0.0164~0.0263    3.08~4.93
#     * estimated visually from Fig 1, Lash et al. 1999
#     ** Fig 1A, data from 50~500 ppm headspace at 60 min
#           and Fig 1B, data at 100~5000 ppm in headspace for 120 min
#     *** Fig 1B, 30~100 ppm headspace, converted to blood concentration
#           using blood:air PC of 9.5
#     **** Fig 1A, data at 50 ppm headspace at 120 min and Fig 1B, data
at
#           25 and 50 ppm headspace at 120 min.
# Overall, human liver hepatocytes are probably most like the
```

```
#     intact liver (e.g., accounting for the competition between
#     GSH conjugation and oxidation).  So central estimates based
#     on those: CLeff ~ 0.32 ml/min/g tissue, KM ~ 0.022 mM in blood.
#     CLeff converted to 19 l/hr/kg; KM converted to 2.9 mg/l in blood
#     However, uncertainty in CLeff is large (values in cytosol
#     ~100-fold larger).  Moreover, Green et al. 1997 reported
#     DCVG formation in cytosol that was ~30,000-fold smaller
#     than Lash et al. (1998) in cytosol, which would be a VMax
#     ~300-fold smaller than Lash et al. (1998) in hepatocytes.
#     Uncertainty in KM appears smaller (~4-fold)
#     CLC: GM = 19., GSD = 100; KM: GM = 2.9., GSD = 4.
#     In addition, at a single concentration, the variability
#     in human liver cytosol samples had a GSD=1.3.
# For the human kidney, the kidney cytosol values are used, with the same
#     uncertainty as for the liver.  Note that the DCVG formation rates
#     in rat kidney cortical cells and rat cytosol are quite similar
#     (see below).
#     CLC: GM = 230., GSD = 100; KM: GM = 2.7., GSD = 4.
# Rat and mouse in vitro data from Lash et al. 1995,1998 define rat and
mouse
#     priors.  However, rats and mice are only assayed at 1 and 2 mM
#     providing only a bound on VMax and very little data on KM.
#                 Rate at 2 mM     Equivalent  CLeff
#                             blood conc. at 2 mM
#                 (nmol/min/  (mM)        (ml/min/
#                 g tissue)               g tissue)
#                        ---------------------------------------------
# Rat      hepatocytes:     4.4~16           2.0        0.0022~0.0079
#     liver cytosol:   8.0~12           1.7~2.0          0.0040~0.0072
#     kidney cells:    0.79~1.1   2.2         0.00036~0.00049
#     kidney cytosol:  0.53~0.75  1.1~2.0          0.00027~0.00068
# Mouse    liver cytosol:   36~40       1.1~2.0          0.018~0.036
#     kidney cytosol:  6.2~9.3          0.91~2.0   0.0031~0.0102
#
# In most cases, rates were increased over the same sex/species at 1 mM,
#     indicating VMax has not yet been reached.  The values between cells
#     and cytosol are more much consistent that in the human data.
#     These data therefore put a lower bound on VMax and a lower bound
#     on CLC.  To account for in vitro-in vivo uncertainty, the lower
#     bound of the prior distribution is set 100-fold below the central
#     estimate of the measurements here.  In addition, Green et al.
#     (1997) found values 100-fold smaller than Lash et al. 1995, 1998.
#     Therefore diffuse prior distributions set to 1e-2~1e4.
# Rat liver: Bound on VMax of 4.4~16, with GM of 8.4.  Converting to
#     mg/hr/kg tissue (* 131.4 ng/nmol * 60 min/hr * 1e3 g/kg / 1e6
mg/ng)
#     gives a central estimate of 66. mg/hr/kg tissue.  Bound on CL of
#     0.0022~0.0079, with GM of 0.0042.  Converting to l/hr/kg tissue
#     (* 60 min/hr) gives 0.25 l/hr/kg tissue.
# Rat kidney: Bound on VMax of 0.53~1.1, with GM of 0.76.  Converting
#     to mg/hr/kg tissue gives a central estimate of 6.0 mg/hr/kg.
#     Bound on CL of 0.00027~0.00068, with GM of 0.00043.  Converting
#     to l/hr/kg tissue gives 0.026 l/hr/kg tissue.
# Mouse liver: Bound on VMax of 36~40, with GM of 38.  Converting
```

```
#     to mg/hr/kg tissue gives a central estimate of 300. mg/hr/kg.
#     Bound on CL of 0.018~0.036, with GM of 0.025.  Converting
#     to l/hr/kg tissue gives 1.53 l/hr/kg tissue.
# Mouse kidney: Bound on VMax of 6.2~9.3, with GM of 7.6.  Converting
#     to mg/hr/kg tissue gives a central estimate of 60. mg/hr/kg.
#     Bound on CL of 0.0031~0.0102, with GM of 0.0056.  Converting
#     to l/hr/kg tissue gives 0.34 l/hr/kg tissue.

# apply interstrain scaling to both liver and kidney
      VMaxDCVG = exp(lnISConj) * VLiv*(Species == 3 ?
(300.*exp(lnVMaxDCVGC)) : (Species == 2 ? (66.*exp(lnVMaxDCVGC)) :
(2.9*19.*exp(lnClDCVGC+lnKMDCVGC))));
        KMDCVG = (Species == 3 ? (VMaxDCVG/(VLiv*1.53*exp(lnClDCVGC))) :
(Species == 2 ? (VMaxDCVG/(VLiv*0.25*exp(lnClDCVGC))) :
2.9*exp(lnKMDCVGC)));
      ClDCVG = VMaxDCVG/KMDCVG;#(vrisk)
      VMaxKidDCVG = exp(lnISConj) * VKid*(Species == 3 ?
(60.*exp(lnVMaxKidDCVGC)) : (Species == 2 ? (6.0*exp(lnVMaxKidDCVGC)) :
(2.7*230.*exp(lnClKidDCVGC+lnKMKidDCVGC))));
        KMKidDCVG = (Species == 3 ?
(VMaxKidDCVG/(VKid*0.34*exp(lnClKidDCVGC))) : (Species == 2 ?
(VMaxKidDCVG/(VKid*0.026*exp(lnClKidDCVGC))) : 2.7*exp(lnKMKidDCVGC)));

# TCE Metabolism Constants for Chloral Kinetics in Lung (mg/hr)
#(v1.2)     Scaled to liver VMax using data from Green et al. (1997)
#     in microsomal preparations (nmol/min/mg protein) at ~1 mM.
#     For humans, used detection limit of 0.03
#     Additional scaling by lung/liver weight ratio
#     from Brown et al. Table 21 (mouse and rat) or
#     ICRP Pub 89 Table 2.8 (Human female and male)
#     Uncertainty ~ 3-fold truncated at 3 GSD
   VMaxClara = exp(lnVMaxLungLivC) * VMax *
      (Species == 3 ? (1.03/1.87*0.7/5.5):(Species == 2 ?
(0.08/0.82*0.5/3.4):(0.03/0.33*(Male == 0 ? (0.42/1.4) : (0.5/1.8)))));
   KMClara = exp(lnKMClara);
#(v1.2)   VMaxClara = KMClara*exp(lnClClaraC)*BW75;
#(v1.2)   VMaxClear = exp(lnVMaxClearC) * BW75;
#(v1.2)   KMClear = exp(lnKMClear);
#(v1.2) Fraction of Respiratory Metabolism that goes to system
circulation
# (translocated to the liver)
   FracLungSys = exp(lnFracLungSysC)/(1 + exp(lnFracLungSysC));

# TCOH Metabolism Constants (mg/hr)
      # No in vitro data.  So use diffuse priors of
      #     1e-4 to 1e4 mg/hr/kg^0.75 for VMax
      #           (4e-5 to 4000 mg/hr for rat),
      #     1e-4 to 1e4 mg/l for KM,
      #     and 1e-5 to 1e3 l/hr/kg^0.75 for Cl
      #           (2e-4 to 2.4e4 l/hr for human)
      VMaxTCOH = BW75*
            (Species == 3 ? (exp(lnVMaxTCOHC)) : (Species == 2 ?
(exp(lnVMaxTCOHC)) : (exp(lnClTCOHC+lnKMTCOH))));
      KMTCOH = exp(lnKMTCOH);
```

```
      VMaxGluc = BW75*
            (Species == 3 ? (exp(lnVMaxGlucC)) : (Species == 2 ?
(exp(lnVMaxGlucC)) : (exp(lnClGlucC+lnKMGluc))));
      KMGluc = exp(lnKMGluc);
      # No in vitro data.  So use diffuse priors of
      #     1e-5 to 1e3 kg^0.25/hr (3.5e-6/hr to 3.5e2/hr for human)
      kMetTCOH = exp(lnkMetTCOHC) / BW25;

# TCA kinetic parameters
      # Central estimate based on GFR clearance per unit body weight
      #     10.0, 8.7, 1.8 ml/min/kg for mouse, rat, human
      #     (= 0.6, 0.522, 0.108 l/hr/kg) from Lin 1995.
      #     = CL_GFR / BW (BW=0.02 for mouse, 0.265 for rat, 70 for
human)
      #     kUrn = CL_GFR / VPlas
      #     Diffuse prior with uncertainty of up,down 100-fold
      # add interstrain variability
      kUrnTCA = exp(lnISkTCA) * exp(lnkUrnTCAC) * BW / VPlas *
            (Species == 3 ? 0.6 : (Species == 2 ? 0.522 : 0.108));
      # No in vitro data.  So use diffuse priors of
      #     1e-4 to 1e2 /hr/kg^0.25 (0.3/hr to 35/hr for human)
      kMetTCA = exp(lnISkTCA) * exp(lnkMetTCAC) / BW25;

# TCOG kinetic parameters
      # No in vitro data.  So use diffuse priors of
      #     1e-4 to 1e2 /hr/kg^0.25 (0.3/hr to 35/hr for human)
      kBile = exp(lnkBileC) / BW25;
        kEHR = exp(lnkEHRC) / BW25;
      # Central estimate based on GFR clearance per unit body weight
      #     10.0, 8.7, 1.8 ml/min/kg for mouse, rat, human
      #     (= 0.6, 0.522, 0.108 l/hr/kg) from Lin 1995.
      #     = CL_GFR / BW (BW=0.02 for mouse, 0.265 for rat, 70 for
human)
      #     kUrn = CL_GFR / VBld
      #     Diffuse prior with Uncertainty of up,down 1000-fold
      kUrnTCOG = exp(lnkUrnTCOGC) * BW / (VBodTCOH * PBodTCOG) *
            (Species == 3 ? 0.6 : (Species == 2 ? 0.522 : 0.108));

# DCVG Kinetics (/hr)
      # Fraction of renal TCE GSH conj. "directly" to DCVC via "first
pass"
      # exp(lnFracOtherCC) = ratio of direct/non-direct
      # Diffuse prior distribution: loguniform 1e-3 to 1e3
      #(v1.2.3) FIXED
      FracKidDCVC = exp(lnFracKidDCVCC)/(1 + exp(lnFracKidDCVCC));
      # No in vitro data.  So use diffuse priors of
      #     1e-4 to 1e2 /hr/kg^0.25 (0.3/hr to 35/hr for human)
        # add interstrain variability
      kDCVG = exp(lnISkDCVG) * exp(lnkDCVGC) / BW25;

# DCVC Kinetics (/hr)#(v2.0)
      # No in vitro data.  So use diffuse priors of
      #     1e-4 to 1e2 /hr/kg^0.25 (0.3/hr to 35/hr for human)
        # add interstrain variability
```

```
      kElimDCVC = exp(lnISkDCVC) * exp(lnkElimDCVCC) / BW25;#(v2.0)

# DCA Kinetics (/hr)#(v2.0)
        # add interstrain variability
      kClearDCA = exp(lnISkDCA) * exp(lnkClearDCAC) / BW25;#(v2.0)
      kDCAcen_per = exp(lnkDCAcen_perC) / BW25;#(v2.0)
      kDCAper_cen = exp(lnkDCAper_cenC) / BW25;#(v2.0)

# CC data initialization
      Rodents = (CC > 0 ? NRodents : 0.0); # Closed chamber simulation
      VCh = (CC > 0 ? VChC - (Rodents * BW) : 1.0);
            # Calculate net chamber volume
      kLoss = (CC > 0 ? exp(lnkLossC) : 0.0);

#*******************************************************************
******
#***                 State Variable Initialization and Scaling
***
#*******************************************************************
******
# NOTE: All State Variables are set to 0 initially, unless re-initialized
here

      ACh = (CC * VCh * MWTCE) / 24450.0;    # Initial amount in chamber

#printf("ClC=%lf\n KM=%lf\n kDCVGC=%lf\n FracTCA=%lf\n ClClaraC=%lf\n
KMClara=%lf\n kDissoc=%lf\n BMax=%lf\n ClTCOHC=%lf\n KMTCOH=%lf\n
ClGlucC=%lf\n KMGluc=%lf\n kElimDCVCC=%lf\n kTSD=%lf\n kAD=%lf\n
kBileC=%lf\n kEHRC=%lf\n kUrnTCAC=%lf\n kUrnTCOGC=%lf\n TCAPlas=%lf\n
VPR=%lf\n lnQCC=%lf\n QFatC=%lf\n QGutC=%lf\n QLivC=%lf\n QSlwC=%lf\n
QTBC=%lf\n VFatBldC=%lf\n VFatC=%lf\n VGutC=%lf\n VLivC=%lf\n VRapC=%lf\n
VTBC=%lf\n VBldC=%lf\n FracPlas=%lf\n VBodC=%lf\n VDTCOHC=%lf\n PB=%lf\n
PFat=%lf\n PGut=%lf\n PLiv=%lf\n PRap=%lf\n PSlw=%lf\n PTB=%lf\n
PAFatC1=%lf\n PAFatC2=%lf\n PBodTCA=%lf\n PLivTCA=%lf\n", #(v2.0)
#ClC, KM, kDCVGC, FracTCA, ClClaraC, KMClara, kDissoc, BMax, ClTCOHC,
KMTCOH, ClGlucC, KMGluc, kElimDCVCC, kTSD, kAD, kBileC, kEHRC, kUrnTCAC,
kUrnTCOGC, TCAPlas, VPR, lnQCC, QFatC, QGutC, QLivC, QSlwC, QTBC,
VFatBldC, VFatC, VGutC, VLivC, VRapC, VTBC, VBldC, FracPlas, VBodC,
VDTCOHC, PB, PFat, PGut, PLiv, PRap, PSlw, PTB, PAFatC1, PAFatC2,
PBodTCA, PLivTCA);#(v2.0)
};
###################### End of Initialization #######################

Dynamics{

#  Some test print statements used to test and debug the translation.
#  printf("\nt=%lf\tCArt=%lf\tCVRap=%lf\tQRap=%lf\tPRap=%lf\tVRap=%lf\n",
*(pdTime), CArt, CVRap, QRap, PRap, VRap);
#  printf("QFat=%lf\tQGutLiv=%lf\tQSlw=%lf\tQRap=%lf\tQTB=%lf\n", QFat,
QGutLiv, QSlw, QRap, QTB);
#  printf("CVTB=%lf\n", rgModelVars[ID_CVTB]);
#  printf("QP=%lf\tQC=%lf\tPB=%lf\n", QP, QC, PB);
#  printf("t=%lf\tCArt=%lf\tCVen=%lf\tCVTB=%lf\n", *(pdTime), CArt,
rgModelVars[ID_CVen], rgModelVars[ID_CVTB]);
```

```
#  printf("QBal = %lf\t", QFat+QGutLiv+QSlw+QRap+QTB - QC);
#  printf("VTot = %lf\tBW=%lf\n", VFat+VFatBld+VGut+VLiv+VSlw+VRap+VTB,
BW);


#************************************************************************
******
#***                     Dynamic physiological parameter scaling
***
#************************************************************************
******
# State Variables with dynamics:
#     none
# Input Variables:
#     QPmeas
# Other State Variables and Global Parameters:
#     QC
#     VPR
#     DResptmp
#     QPsamp
#     QFatCtmp
#     QGutCtmp
#     QLivCtmp
#     QSlwCtmp
#(v1.2)     QTBCtmp
#     QKidCtmp
#     FracPlas
# Temporary variables used:
#     none
# Temporary variables assigned:
#     QP
#     DResp
#     QCnow
#     QFat
#     QGut
#     QLiv
#     QSlw
#(v1.2)     QTB
#     QKid
#     QGutLiv
#     QRap
#     QCPlas
#     QBodPlas
#     QGutLivPlas
# Notes:
#************************************************************************
******

# QP uses QPmeas if value is > 0, otherwise uses sampled value
      QP = (QPmeas > 0 ? QPmeas : QPsamp);
#(v1.2) Scale diffusion flow rate
      DResp = DResptmp * QP;

# QCnow uses QPmeas/VPR if QPmeas > 0, otherwise uses sampled value
```

```
      QCnow = (QPmeas > 0 ? QPmeas/VPR : QC);

# These done here in dynamics in case QCnow changes
# Blood Flows to Tissues (L/hr)
        QFat = (QFatCtmp) * QCnow; #
        QGut = (QGutCtmp) * QCnow; #
        QLiv = (QLivCtmp) * QCnow; #
        QSlw = (QSlwCtmp) * QCnow; #
#(v1.2)         QTB = (QTBCtmp) * QCnow; #

        QKid = (QKidCtmp) * QCnow; #
     QGutLiv = QGut + QLiv; #
#(v1.2)        QRap = QCnow - QFat - QGut - QLiv - QSlw - QTB - QKid;
      QRap = QCnow - QFat - QGut - QLiv - QSlw - QKid; #(v1.2)
           ##          #(v1b)#(v1c) don't subtract off TB
      QBod = QCnow - QGutLiv;

  QFatSC = QFat/QCnow; # (vrisk3) #Scaled fat blood flow
  QGutSC = QGut/QCnow; # (vrisk3) #Scaled gut blood flow
  QLivSC = QLiv/QCnow; # (vrisk3) #Scaled liver blood flow
  QSlwSC = QSlw/QCnow; # (vrisk3) #Scaled slowly perfused blood flow
  QRapSC = QRap/QCnow; # (vrisk3) #Scaled rapidly perfused blood flow
  QKidSC = QKid/QCnow; # (vrisk3) #Scaled kidney blood flow

# Plasma Flows to Tissues (L/hr)
      QCPlas = FracPlas * QCnow; #
    QBodPlas = FracPlas * QBod; #
    QGutLivPlas = FracPlas * QGutLiv; #

  VFatSC=VFat/BW; # (vrisk3) #Fat fractional compartment volume
  VGutSC=VGut/BW; # (vrisk3) #Gut fractional compartment volume
  VLivSC=VLiv/BW; # (vrisk3) #Liver fractional compartment volume
  VRapSC=VRap/BW; # (vrisk3) #Rapidly perfused fractional compartment
volume
  VRespLumSC=VRespLum/BW; # (vrisk3) # Fractional volume of respiratory
lumen
  VRespEffSC=VRespEff/BW; # (vrisk3) #Effective fractional volume of
respiratory tissue
  VKidSC=VKid/BW; # (vrisk3) #Kidney fractional compartment volume
  VBldSC=VBld/BW; # (vrisk3) #Blood fractional compartment volume
  VSlwSC=VSlw/BW; # (vrisk3) #Slowly perfused fractional compartment
volume
  VPlasSC=VPlas/BW; # (vrisk3) #Plasma fractional compartment volume
  VBodSC=VBod/BW; # (vrisk3) #TCA Body fractional compartment volume [not
incl. blood+liver]
  VBodTCOHSC=VBodTCOH/BW; # (vrisk3) #TCOH/G Body fractional compartment
volume [not incl. liver]


#*******************************************************************
******
#***                  Exposure and Absorption calculations
***
```

```
#***********************************************************************
******
# State Variables with dynamics:
#     AStom
#     ADuod
#     AStomTCA
#     AStomTCOH
# Input Variables:
#     IVDose
#     PDose
#     Drink
#     Conc
#     IVDoseTCA
#     PODoseTCA
#     IVDoseTCOH
#     PODoseTCOH
# Other State Variables and Global Parameters:
#     ACh
#     CC
#     VCh
#     MWTCE
#     BW
#     TChng
#     kAS
#     kTSD
#     kAD
#     kTD
#     kASTCA
#     kASTCOH
# Temporary variables used:
#     none
# Temporary variables assigned:
#     kIV - rate into CVen
#     kIA - rate into CArt
#     kPV - rate into portal vein
#     kStom - rate into stomach (v1.2.1)
#     kDrink - incorporated into RAO
#     RAO - rate into gut (oral absorption - both gavage and drinking
water)
#     CInh - inhalation exposure concentration
#     kIVTCA - rate into blood
#     kStomTCA - rate into stomach (v1.2.1)
#     kPOTCA - rate into liver (oral absorption)
#     kIVTCOH - rate into blood
#     kStomTCOH - rate into stomach (v1.2.1)
#     kPOTCOH - rate into liver (oral absorption)
# Notes:
# For oral dosing, using "Spikes" for instantaneous inputs
# Inhalation Concentration (mg/L)
#     CInh uses Conc when open chamber (CC=0) and
#     ACh/VCh when closed chamber CC>0.
#***********************************************************************
******
```

```
#### TCE DOSING
## IV route
    kIV = (IVDose * BW) / TChng;# IV infusion rate (mg/hr)
                       # (IVDose constant for duration TChng)
    kIA = (IADose * BW) / TChng;  # IA infusion rate (mg/hr) (v1f)
    kPV = (PVDose * BW) / TChng;  # PV infusion rate (mg/hr) (v1f)
    kStom = (PDose * BW) / TChng;# PO dose rate (into stomach) (mg/hr)
(v1.2.1)

## Oral route
# Amount of TCE in stomach -- for oral dosing only (mg)
#(v1.2.1)    dt(AStom) = PDose * BW - AStom * (kAS + kTSD);
    dt(AStom) = kStom - AStom * (kAS + kTSD);

# Amount of TCE in duodenum -- for oral dosing only (mg)
    dt(ADuod) = (kTSD * AStom) - (kAD + kTD) * ADuod;
# Rate of absorption from drinking water
    kDrink = (Drink * BW) / 24.0; #Ingestion rate via drinking water
(mg/hr)
# Total rate of absorption including gavage and drinking water
    RAO = kDrink + (kAS * AStom) + (kAD * ADuod);
## Inhalation route
    CInh = (CC > 0 ? ACh/VCh : Conc*MWTCE/24450.0); # in mg/l

#### TCA Dosing
      kIVTCA = (IVDoseTCA * BW) / TChng;  # TCA IV infusion rate (mg/hr)
      kStomTCA =  TCABgd * BW / 24 + #(v2.0) TCA background in mg/kg/d
             (PODoseTCA * BW) / TChng; # TCA PO dose rate into stomach
#(v1.2.1)    dt(AStomTCA) = PODoseTCA * BW - AStomTCA * kASTCA;
      dt(AStomTCA) = kStomTCA - AStomTCA * kASTCA;
      kPOTCA = AStomTCA * kASTCA;  # TCA oral absorption rate (mg/hr)

#### TCOH Dosing
      kIVTCOH = (IVDoseTCOH * BW) / TChng;#TCOH IV infusion rate (mg/hr)
      kStomTCOH = (PODoseTCOH * BW) / TChng; # TCOH PO dose rate into
stomach
#(v1.2.1.)    dt(AStomTCOH) = PODoseTCOH * BW - AStomTCOH * kASTCOH;
    dt(AStomTCOH) = kStomTCOH - AStomTCOH * kASTCOH;
      kPOTCOH = AStomTCOH * kASTCOH;# TCOH oral absorption rate (mg/hr)

#*************************************************************************
******
#***                        TCE Model
***
#*************************************************************************
******
# State Variables with dynamics:
#     ARap,       # Amount in rapidly perfused tissues
#     ASlw,       # Amount in slowly perfused tissues
#     AFat,       # Amount in fat
#     AGut,       # Amount in gut
#     ALiv,       # Amount in liver
#(v1.2)     ATB,        # Amount in TB -- candidate to remove and put in
Rap
```

```
#     AInhResp,
#     AResp,
#     AExhResp,
#     AKid,            # Amount in Kidney -- currently in Rap tissue
#     ABld,            # Amount in Blood -- currently in Rap tissue
#     ACh,        # Amount of TCE in closed chamber
# Input Variables:
#     none
# Other State Variables and Global Parameters:
#     VRap
#     PRap
#     VSlw
#     PSlw
#     VFat
#     PFat
#     VGut
#     PGut
#     VLiv
#     PLiv
#(v1.2)     VTB
#(v1.2)     PTB
#     VRespLum
#     VRespEff
#     FracLungSys
#     VKid
#     PKid
#     VBld
#     VMaxClara
#     KMClara
#     PB
#     Rodents
#     VCh
#     kLoss
#     VMax
#     KM
#     VMaxDCVG
#     KMDCVG
#     VMaxKidDCVG
#     KMKidDCVG
# Temporary variables used:
#     QM
#     QFat
#     QGutLiv
#     QSlw
#     QRap
#(v1.2)     QTB
#     QKid
#     kIV
#     QCnow
#     CInh
#     QP
#     RAO
# Temporary variables assigned:
#     QM
```

```
#     CRap
#     CSlw
#     CFat
#     CGut
#     CLiv
#(v1.2)     CTB
#     CInhResp
#     CResp
#     CExhResp
#     ExhFactor
#     CMixExh
#     CKid
#     CVRap
#     CVSlw
#     CVFat
#     CVGut
#     CVLiv
#     CVTB
#     CVKid
#     CVen
#     RAMetLng
#     CArt_tmp
#     CArt
#     CAlv
#     RAMetLiv1
#     RAMetLiv2
#     RAMetKid
# Notes:
#**************************************************************************
******
#
#(v1.2)     ATB = (VMaxClara > RTB) ? (RTB*KMClara*VTB*PTB/(VMaxClara -
RTB)) : 0; #(v1j)

#****Blood
(venous)************************************************************
# Tissue Concentrations (mg/L)
      CRap = ARap/VRap;
      CSlw = ASlw/VSlw;
      CFat = AFat/VFat;
      CGut = AGut/VGut;
      CLiv = ALiv/VLiv;
#(v1.2)     CTB = (ATB<0 ? 0 : ATB/VTB); # Comment out for #(v1b)#(v1c)
      CKid = AKid/VKid;
# Venous Concentrations (mg/L)
      CVRap = CRap / PRap;
      CVSlw = CSlw / PSlw;
      CVFat = CFat / PFat;
      CVGut = CGut / PGut;
      CVLiv = CLiv / PLiv;
#(v1.2)     CVTB = CTB / PTB;# Comment out for #(v1b)#(v1c)
      CVKid = CKid / PKid;
# Concentration of TCE in mixed venous blood (mg/L)
      CVen = ABld/VBld; # Comment out for #(v1a)#(v1c)
```

```
          ##    CVen = (QFat*CVFat + QGutLiv*CVLiv + QSlw*CVSlw +
#(v1a)#(v1c)
          ##          QRap*CVRap + QTB*CVTB + QKid*CVKid + kIV) / QCnow;
#(v1a)
          ##          QRap*CVRap + QKid*CVKid + kIV) / QCnow; #(v1c)
# Dynamics for blood
    dt(ABld) = (QFat*CVFat + QGutLiv*CVLiv + QSlw*CVSlw + # Comment out
for #(v1a)#(v1c)
          QRap*CVRap + QKid*CVKid + kIV) - CVen * QCnow; #(v1.2)(v1b)
#(v1.2)          QRap*CVRap + QTB*CVTB + QKid*CVKid + kIV) - CVen *
QCnow;# Comment out for #(v1a)#(v1c)

#****Gas exchange
***********************************************************
# Concentration in arterial blood leaving lung (mg/L) #(v1f)
#(v1.2)    CArt_tmp = ((QCnow * CVen) + (QP * CInh)) / (QCnow + (QP /
PB));

#****Respiratory
Metabolism****************************************************
#(v1.2)
    QM = QP/0.7; # Minute-volume
    CInhResp = AInhResp/VRespLum;
    CResp = AResp/VRespEff;
    CExhResp = AExhResp/VRespLum;
    dt(AInhResp) = (QM*CInh + DResp*(CResp-CInhResp) - QM*CInhResp);
    RAMetLng = VMaxClara * CResp/(KMClara + CResp);
    dt(AResp) = (DResp*(CInhResp + CExhResp - 2*CResp) - RAMetLng);
    CArt_tmp = (QCnow*CVen + QP*CInhResp)/(QCnow + (QP/PB));
    dt(AExhResp) = (QM*(CInhResp-CExhResp) + QP*(CArt_tmp/PB-CInhResp) +
          DResp*(CResp-CExhResp));
    CMixExh = (CExhResp > 0 ? CExhResp : 1e-15); #(v1.2) mixed exhaled
breath

# Concentration in alveolar air (mg/L) #(v1f)
#(v1.2)        CAlv = CArt_tmp / PB;
      #(v1.2) Correction factor for exhaled air to account for
      # absorption/desorption/metabolism in respiratory tissue
      # = 1 if DResp = 0
      ExhFactor_den = (QP * CArt_tmp / PB + (QM-QP)*CInhResp);
      ExhFactor = (ExhFactor_den > 0) ? (
            QM * CMixExh / ExhFactor_den) : 1;
      #(v1.2) End-exhaled breath (corrected for absorption/
      #     desorption/metabolism in respiratory tissue)
      CAlv = CArt_tmp / PB * ExhFactor;
# Concentration in arterial blood entering circulation (mg/L) #(v1f)
      CArt = CArt_tmp + kIA/QCnow; # add inter-arterial dose

#****Other dynamics for inhalation/exhalation
*******************************
# Dynamics for amount of TCE in closed chamber
#(v1.2)    dt(ACh) = (Rodents * (QP * CAlv - QP * ACh/VCh)) - (kLoss *
ACh);
    dt(ACh) = (Rodents * (QM * CMixExh - QM * ACh/VCh)) - (kLoss * ACh);
```

```
      # (v1.2)
#**** TB region
****************************************************************
# Amount metabolized in the tracheo-bronchial region (mg)
#(v1j)      RAMetLng = (VMaxClara * CVTB) / (KMClara + CVTB); ## comment
out for #(v1b)#(v1c)
#(v1.2)     RAMetLng = RTB; #(v1j)
# Amount of TCE in the tracheo-bronchial region (mg)
#(v1j)         dt(ATB) = QTB*(CArt - CVTB) - RAMetLng; ## comment out for
#(v1b)#(v1c)
#(v1.2)           ZTB = (VMaxClara > RTB) ? (VMaxClara - RTB) : 0;
#(v1.2)         dt(RTB) = ZTB*(QTB*CArt*ZTB - RTB*(KMClara*QTB + ZTB))/
#(v1j)
#(v1.2)                 (VMaxClara*KMClara*PTB*VTB); #(v1j)
# Venous blood draining TB (v1b)(v1c)
##    a = (VMaxClara/QTB + KMClara - CArt)/2;#(v1b)#(v1c)
##    b = CArt*KMClara;#(v1b)#(v1c)
##    CVTB = (b < 0.01*a*a ? b/2.0/a : sqrt(a*a+b)-a);#(v1b)#(v1c)
# Amount of TCE in the tracheo-bronchial region (mg) (v1b)#(v1c)
##       CTB = CVTB * PTB;#(v1b)#(v1c)
# Amount metabolized in the tracheo-bronchial region (mg) (v1b)#(v1c)
##    a = (VMaxClara + QTB*(KMClara + CArt))/2;#(v1b)#(v1c)
##    b = CArt*QTB*VMaxClara;#(v1b)#(v1c)
##    RAMetLng = (b < 0.01*a*a ? b/2.0/a : a-sqrt(a*a-b));#(v1b)#(v1c)

#**** Non-metabolizing tissues
************************************************
# Amount of TCE in rapidly perfused tissues (mg)
    dt(ARap) = QRap * (CArt - CVRap); ##      - RAMetLng; #(v1b)#(v1c)
# Amount of TCE in slowly perfused tissues
    dt(ASlw) = QSlw * (CArt - CVSlw);
# Amount of TCE in fat tissue (mg)
    dt(AFat) = QFat*(CArt - CVFat);
# Amount of TCE in gut compartment (mg)
    dt(AGut) = (QGut * (CArt - CVGut)) + RAO;

#**** Liver
********************************************************************
# Rate of TCE oxidation by P450 to TCA, TCOH, and other (DCA) in liver
(mg/hr)
      RAMetLiv1 = (VMax * CVLiv) / (KM + CVLiv);
# Rate of TCE metabolized to DCVG in liver (mg)
      RAMetLiv2 = (VMaxDCVG * CVLiv) / (KMDCVG + CVLiv);
# Dynamics for amount of TCE in liver (mg)
    dt(ALiv) = (QLiv * (CArt - CVLiv)) + (QGut * (CVGut - CVLiv))
                 - RAMetLiv1 - RAMetLiv2 + kPV; # add PV dose #(v1f)

#**** Kidney
*******************************************************************
# Rate of TCE metabolized to DCVG in kidney (mg) #
      RAMetKid = (VMaxKidDCVG * CVKid) / (KMKidDCVG + CVKid);
# Amount of TCE in kidney compartment (mg)
    dt(AKid) = (QKid * (CArt - CVKid)) - RAMetKid;
```

```
#*************************************************************************
******
#***                    TCOH Sub-model
***
#*************************************************************************
******
# State Variables with dynamics:
#     ABodTCOH
#     ALivTCOH
# Input Variables:
#     none
# Other State Variables and Global Parameters:
#     ABileTCOG
#     kEHR
#     VBodTCOH
#     PBodTCOH
#     VLiv
#     PLivTCOH
#     VMaxTCOH
#     KMTCOH
#     VMaxGluc
#     KMGluc
#     kMetTCOH - hepatic metabolism of TCOH (e.g., to DCA)
#     FracOther
#     FracTCA
#     StochTCOHTCE
#     StochTCOHGluc
#     FracLungSys
# Temporary variables used:
#     QBod
#     QGutLiv
#     QCnow
#     kPOTCOH
#     RAMetLiv1
#     RAMetLng
# Temporary variables assigned:
#     CVBodTCOH
#     CVLivTCOH
#     CTCOH
#     RAMetTCOHTCA
#     RAMetTCOHGluc
#     RAMetTCOH
#     RARecircTCOG
# Notes:
#*************************************************************************
******
#**** Blood (venous=arterial)
*************************************************
# Venous Concentrations (mg/L)
      CVBodTCOH = ABodTCOH / VBodTCOH / PBodTCOH;
      CVLivTCOH = ALivTCOH / VLiv / PLivTCOH;
      CTCOH = (QBod * CVBodTCOH + QGutLiv * CVLivTCOH + kIVTCOH)/QCnow;
```

```
#**** Body
**********************************************************************
# Amount of TCOH in body
    dt(ABodTCOH) = QBod * (CTCOH - CVBodTCOH);

#**** Liver
*********************************************************************

# Rate of oxidation of TCOH to TCA (mg/hr)
      RAMetTCOHTCA = (VMaxTCOH * CVLivTCOH) / (KMTCOH + CVLivTCOH);
# Amount of glucuronidation to TCOG (mg/hr)
      RAMetTCOHGluc = (VMaxGluc * CVLivTCOH) / (KMGluc + CVLivTCOH);
# Amount of TCOH metabolized to other (e.g., DCA)
      RAMetTCOH = kMetTCOH * ALivTCOH;
# Amount of TCOH-Gluc recirculated (mg)
      RARecircTCOG = kEHR * ABileTCOG;
# Amount of TCOH in liver (mg)
    dt(ALivTCOH) = kPOTCOH + QGutLiv * (CTCOH - CVLivTCOH)
            - RAMetTCOH - RAMetTCOHTCA - RAMetTCOHGluc
            + ((1.0 - FracOther - FracTCA) * StochTCOHTCE *
            (RAMetLiv1 + FracLungSys*RAMetLng)) #(v1.2)
            + (StochTCOHGluc * RARecircTCOG);

#(v2.0)
#*********************************************************************
******
#***                      DCA Sub-model
***
#*********************************************************************
******
# State Variables with dynamics:
#     ADCA
#     ADCAper
# Input Variables:
#     None
# Other State Variables and Global Parameters:
#     FracOther
#     StochDCATCE
#     FracLungSys
#     kClearDCA
#     kDCAcen_per
#     kDCAper_cen
#     VDCA
# Temporary variables used:
#     none
# Temporary variables assigned:
#       none
# Notes:
#
#*********************************************************************
******
# Amount of DCA in central compartment (mg)
    dt(ADCA) = DCABgd * BW / 24 + #(v2.0) DCA background in mg/kg/d
            (FracOther * StochDCATCE *
```

```
          (RAMetLiv1 + FracLungSys*RAMetLng)) - (kClearDCA * ADCA) -
          kDCAcen_per * ADCA + kDCAper_cen * ADCAper;
# Amount of DCA in peripheral compartment (mg)
    dt(ADCAper) = kDCAcen_per * ADCA - kDCAper_cen * ADCAper;
# Concentration of DCA in blood (in mmoles/l)
      CDCAmol = ADCA / MWDCA / VDCA;
#*************************************************************************
******
#***                    TCA Sub-model
***
#*************************************************************************
******
# State Variables with dynamics:
#     APlasTCA # comment out for #(v1h)
#     ABodTCA
#     ALivTCA
#     AUrnTCA
#     AUrnTCA_sat
#     AUrnTCA_collect
# Input Variables:
#     TCAUrnSat
#     UrnMissing
# Other State Variables and Global Parameters:
#     VPlas
#     MWTCA
#     kDissoc
#     BMax
#     kMetTCA -- hepatic metabolism of TCA (e.g., to DCA)
#     VBod
#     PBodTCA
#     PLivTCA
#     kUrnTCA
#     FracTCA
#     StochTCATCE
#     StochTCATCOH
#     FracLungSys
# Temporary variables used:
#     kIVTCA
#     kPOTCA
#     QBodPlas
#     QGutLivPlas
#     QCPlas
#     RAMetLiv1
#     RAMetTCOHTCA
#     RAMetLng
# Temporary variables assigned:
#     CPlasTCA
#     CPLasTCAMole
#     a, b, c
#     CPlasTCAFreeMole
#     CPlasTCAFree
#     APlasTCAFree
#     CPlasTCABnd
#     CBodTCAFree
```

```
#     CLivTCAFree
#     CBodTCA
#     CLivTCA
#     CVBodTCA
#     CVLivTCA
#     RUrnTCA
#     RAMetTCA
# Notes:
#*****************************************************************
******
#**** Plasma
*****************************************************************
# Concentration of TCA in plasma (umoles/L)
      CPlasTCA = (APlasTCA<1.0e-15 ? 1.0e-15 : APlasTCA/VPlas); # comment
out for #(v1h)
      ## CPlasTCA = (kIVTCA + (QBodPlas*CVBodTCA) +
(QGutLivPlas*CVLivTCA))/QCPlas; #(v1h)
# Concentration of free TCA in plasma in (umoles/L)
      CPlasTCAMole = (CPlasTCA / MWTCA) * 1000.0;
      a = kDissoc+BMax-CPlasTCAMole;
      b = 4.0*kDissoc*CPlasTCAMole;
      c = (b < 0.01*a*a ? b/2.0/a : sqrt(a*a+b)-a);
      CPlasTCAFreeMole = 0.5*c;
# Concentration of free TCA in plasma (mg/L)
      CPlasTCAFree = (CPlasTCAFreeMole * MWTCA) / 1000.0;
      APlasTCAFree = CPlasTCAFree * VPlas;
# Concentration of bound TCA in plasma (mg/L)
      CPlasTCABnd = (CPlasTCA<CPlasTCAFree ? 0 : CPlasTCA-CPlasTCAFree);
# Concentration in body and liver
      CBodTCA = (ABodTCA<0 ? 0 : ABodTCA/VBod);
      CLivTCA = (ALivTCA<1.0e-15 ? 1.0e-15 : ALivTCA/VLiv);
# Total concentration in venous plasma (free+bound)
      CVBodTCAFree = (CBodTCA / PBodTCA);     # free in equilibrium
      CVBodTCA = CPlasTCABnd + CVBodTCAFree;
#     CVBodTCA = (BMax+kDissoc + CVBodTCAFree)*CVBodTCAFree / #(v1g)
#           (kDissoc + CVBodTCAFree);    #(v1g) total in equilibrium
      CVLivTCAFree = (CLivTCA / PLivTCA);
      CVLivTCA = CPlasTCABnd + CVLivTCAFree;  # free in equilibrium
#     CVLivTCA = (BMax+kDissoc + CVLivTCAFree)*CVLivTCAFree / #(v1g)
#           (kDissoc + CVLivTCAFree);    #(v1g) total in equilibrium
# Rate of urinary excretion of TCA
      RUrnTCA = kUrnTCA * APlasTCAFree;
# Dynamics for amount of total (free+bound) TCA in plasma (mg)
    dt(APlasTCA) = kIVTCA + (QBodPlas*CVBodTCA) + (QGutLivPlas*CVLivTCA)
# comment out for #(v1h)
                 - (QCPlas * CPlasTCA) - RUrnTCA; # comment out for
#(v1h)

#**** Body
*****************************************************************
# Dynamics for amount of TCA in the body (mg)
    dt(ABodTCA) = QBodPlas * (CPlasTCAFree - CVBodTCAFree);
      ## dt(ABodTCA) = QBodPlas * (CPlasTCAFree - CVBodTCAFree) -
RUrnTCA; #(v1h)
```

```
#    dt(ABodTCA) = QBodPlas * (CPlasTCA - CVBodTCA); #(v1g)
      ## dt(ABodTCA) = QBodPlas * (CPlasTCA - CVBodTCA) - RUrnTCA;
#(v1g)+(v1h)

#**** Liver
******************************************************************
# Rate of metabolism of TCA
      RAMetTCA = kMetTCA * ALivTCA;
# Dynamics for amount of TCA in the liver (mg)
    dt(ALivTCA) = kPOTCA + QGutLivPlas*(CPlasTCAFree - CVLivTCAFree)
                 - RAMetTCA + (FracTCA * StochTCATCE *
                 (RAMetLiv1 + FracLungSys*RAMetLng)) #(v1.2)
                 + (StochTCATCOH * RAMetTCOHTCA);
#    dt(ALivTCA) = kPOTCA + QGutLivPlas*(CPlasTCA - CVLivTCA) #(v1g)
#                - RAMetTCA + (FracTCA * StochTCATCE * RAMetLiv1) #(v1g)
#                + (StochTCATCOH * RAMetTCOHTCA); #(v1g)

#**** Urine
******************************************************************
# Dynamics for amount of TCA in urine (mg)
    dt(AUrnTCA) = RUrnTCA;
    dt(AUrnTCA_sat) = TCAUrnSat*(1-UrnMissing)* RUrnTCA;
            # Saturated, but not missing collection times
    dt(AUrnTCA_collect) = (1-TCAUrnSat)*(1-UrnMissing)*RUrnTCA;
            # Not saturated and not missing collection times

#*********************************************************************
******
#***                      TCOG Sub-model
***
#*********************************************************************
******
# State Variables with dynamics:
#     ABodTCOG
#     ALivTCOG
#     ABileTCOG
#     AUrnTCOG
#     AUrnTCOG_sat
#     AUrnTCOG_collect
# Input Variables:
#     TCOGUrnSat
#     UrnMissing
# Other State Variables and Global Parameters:
#     VBodTCOH
#     VLiv
#     PBodTCOG
#     PLivTCOG
#     kUrnTCOG
#     kBile
#     StochGlucTCOH
# Temporary variables used:
#     QBod
#     QGutLiv
#     QCnow
```

```
#     RAMetTCOHGluc
#     RARecircTCOG
# Temporary variables assigned:
#     CVBodTCOG
#     CVLivTCOG
#     CTCOG
#     RUrnTCOG
#     RBileTCOG
# Notes:
#*************************************************************************
******
#**** Blood (venous=arterial)
*************************************************
# Venous Concentrations (mg/L)
      CVBodTCOG = ABodTCOG / VBodTCOH / PBodTCOG;
      CVLivTCOG = ALivTCOG / VLiv / PLivTCOG;
      CTCOG = (QBod * CVBodTCOG + QGutLiv * CVLivTCOG)/QCnow;
#**** Body
*******************************************************************
# Amount of TCOG in body
      RUrnTCOG = kUrnTCOG * ABodTCOG;
    dt(ABodTCOG) = QBod * (CTCOG - CVBodTCOG) - RUrnTCOG;

#**** Liver
******************************************************************
# Amount of TCOG in liver (mg)
      RBileTCOG = kBile * ALivTCOG;
    dt(ALivTCOG) = QGutLiv * (CTCOG - CVLivTCOG)
            + (StochGlucTCOH * RAMetTCOHGluc) - RBileTCOG;

#**** Bile
*******************************************************************
# Amount of TCOH-Gluc excreted into bile (mg)
    dt(ABileTCOG) = RBileTCOG - RARecircTCOG;

#**** Urine
******************************************************************
# Amount of TCOH-Gluc excreted in urine (mg)
    dt(AUrnTCOG) = RUrnTCOG;
    dt(AUrnTCOG_sat) = TCOGUrnSat*(1-UrnMissing)*RUrnTCOG;
            # Saturated, but not missing collection times
    dt(AUrnTCOG_collect) = (1-TCOGUrnSat)*(1-UrnMissing)*RUrnTCOG;
            # Not saturated and not missing collection times

#*************************************************************************
******
#***                      DCVG Sub-model
***
#*************************************************************************
******
# State Variables with dynamics:
#     ADCVGmol
# Input Variables:
#     none
```

```
# Other State Variables and Global Parameters:
#     kDCVG
#     FracKidDCVC # Fraction of kidney DCVG going to DCVC in first pass
#     VDCVG
# Temporary variables used:
#     RAMetLiv2
#     RAMetKid
# Temporary variables assigned:
#     RAMetDCVGmol
#     CDCVGmol
# Notes:
#     Assume negligible GGT activity in liver as compared to kidney,
#     supported by in vitro data on GGT (even accounting for 5x
#     greater liver mass relative to kidney mass), as well as lack
#     of DCVC detected in blood.
#     Need to account for "first pass" in kidney (TCE->DCVG->DCVC
#     without systemic circulation).
#*********************************************************************
******
# Rate of metabolism of DCVG to DCVC
     RAMetDCVGmol = kDCVG * ADCVGmol;
# Dynamics for DCVG in blood
    dt(ADCVGmol) = (RAMetLiv2 + RAMetKid*(1-FracKidDCVC)) / MWTCE
           - RAMetDCVGmol;
# Concentration of DCVG in blood (in mmoles/l)
     CDCVGmol = ADCVGmol / VDCVG;

#*********************************************************************
******
#***                       DCVC Sub-model
***
#*********************************************************************
******
# State Variables with dynamics:
#     ADCVC#(v2.0)
# Input Variables:
#     none
# Other State Variables and Global Parameters:
#     MWDCVC
#     FracKidDCVC
#     StochDCVCTCE
#     kElimDCVC#(v2.0)
#     VDCVC#(v2.0)
# Temporary variables used:
#     RAMetDCVGmol
#     RAMetKid
# Temporary variables assigned:
# Notes:#(v2.0)
#*********************************************************************
******
# Amount of DCVC in body (mg) #(v2.0)
    dt(ADCVC) = RAMetDCVGmol * MWDCVC
           + RAMetKid * FracKidDCVC * StochDCVCTCE
           - kElimDCVC * ADCVC;#(v2.0)
```

```
# Concentration of DCVG in blood (in mmoles/l)#(v2.0)
      CDCVCmol = ADCVC / MWDCVC / VDCVC;#(v2.0)
#**************************************************************************
******
#***                      Total Mass Balance
***
#**************************************************************************
******
# State Variables with dynamics:
#     InhDose
#     AO
#     AExc
#     AMetLng
#     AMetLiv1
#     AMetLiv2
#     AMetKid
#     AExh
#     ARecircTCOG
#     AOTCOH
#     AMetTCOHTCA
#     AMetTCOHGluc
#     AMetTCOHOther
#     AOTCA
#     AMetTCA
#     ADCVGIn
#     AMetDCVG
#     ADCVCIn
# Input Variables:
#     IVDose
#     IVDoseTCA
#     IVDOseTCOH
# Other State Variables and Global Parameters:
#     BW
#     AStom
#     ADuod
#     ARap
#     ASlw
#     AFat
#     AGut
#     ALiv
#     ATB
#     AKid
#     ABld
#     FracOther
#     FracTCA
#     StochTCOHTCE
#     StochTCOHGluc
#     ABodTCOH
#     ALivTCOH
#     StochTCATCE
#     StochTCATCOH
#     APlasTCA
#     ABodTCA
#     ALivTCA
```

```
#     AUrnTCA
#     StochGlucTCOH
#     ABodTCOG
#     ALivTCOG
#     ABileTCOG
#     AUrnTCOG
#     FracKidDCVC
#     MWTCE
#     ADCVGmol
#     MWDCVC
#     StochDCVCTCE
#     kKidBioact
#     ADCVC
# Temporary variables used:
#     QP
#     QM
#     CInh
#     CMixExh
#     RAO
#     RInhDose
#     kPOTCA
#     kPOTCOH
#     RAMetLng
#     RAMetLiv1
#     RAMetLiv2
#     RAMetKid
#     CAlv
#     RARecircTCOG
#     RAMetTCOHTCA
#     RAMetTCOHGluc
#     RAMetTCOH
#     RAMetTCA
#     RAMetDCVGmol
# Temporary variables assigned:
#     RInhDose
#     TotDose
#     TotTissue
#     ATotMetLiv
#     TotMetab
#     RAExc - rate of fecal excretion
#     RAExh
#     TCEDiff
#     MassBalTCE
#     TotTCOHIn
#     TotTIssueTCOH
#     TotMetabTCOH
#     MassBalTCOH
#     TotTCAIn
#     TotTissueTCA
#     TCADiff
#     MassBalTCA
#     TotTCOGIn
#     TotTissueTCOG
#     MassBalTCOG
```

```
#     MassBalDCVG
#     MassBalDCVC
# Notes:
#***********************************************************************
******
#**** Mass Balance for TCE
***************************************************
# Total intake from inhalation (mg)
#(v1.2)     RInhDose = QP * CInh;
      RInhDose = QM * CInh;
    dt(InhDose) = RInhDose;
# Amount of TCE absorbed (mg)
#(v1e)    dt(AO) = RAO;
# Total dose
#(v1e)      TotDose = InhDose + AO + IVDose * BW;
# Total in tissues
#(v1e)      TotTissue = # AStom + ADuod +
#(v1e)            ARap + ASlw + AFat + AGut + ALiv + ATB + AKid + ABld;
            ##    ARap + ASlw + AFat + AGut + ALiv + ATB + AKid; #(v1a)
            ##    ARap + ASlw + AFat + AGut + ALiv + AKid + ABld;#(v1b)
            ##    ARap + ASlw + AFat + AGut + ALiv + AKid;#(v1c)
# Total metabolized
    dt(AMetLng) = RAMetLng; #(vrisk)
    dt(AMetLiv1) = RAMetLiv1; #(vrisk)
    dt(AMetLiv2) = RAMetLiv2; #(vrisk)
    dt(AMetKid) = RAMetKid; #(vrisk)
#(v1e)      ATotMetLiv = AMetLiv1 + AMetLiv2;
#(v1e)      TotMetab = AMetLng + ATotMetLiv + AMetKid;
# Amount of TCE excreted in feces (mg)
#(v1e)      RAExc = kTD * ADuod;
#(v1e)    dt(AExc) = RAExc;
# Amount exhaled (mg)
#(v1.2)     RAExh = QP * CAlv;
      RAExh = QM * CMixExh;
    dt(AExh) = RAExh;
# Mass balance
#(v1e)      TCEDiff = TotDose - TotTissue - TotMetab;
#(v1e)      MassBalTCE = TCEDiff - AExc - AExh;

#(v2.0)
#**** Mass Balance for DCA
***************************************************
#(v1e)      TotDCAIn = FracOther * StochDCATCE * (AMetLiv1 +
FracLungSys*AMetLng);#(v2.0)
# Amount of DCA eliminated (mg)#(v2.0)
#(v1e)    dt(AElimDCA) = kClearDCA * ADCA;#(v2.0)
#(v1e)      MassBalDCA = TotDCAIn - ADCA - ADCAper - AElimDCA;#(v2.0)
TotDCAProd = (FracOther * StochDCATCE * #(vrisk)
           (AMetLiv1 + FracLungSys*AMetLng));#(vrisk)

#**** Mass Balance for TCOH
**************************************************
# Total production/intake of TCOH
#(v1e)    dt(ARecircTCOG) = RARecircTCOG;
```

```
#(v1e)    dt(AOTCOH) = kPOTCOH;
#(v1e)      TotTCOHIn = AOTCOH + IVDoseTCOH * BW
#(v1e)             + ((1.0 - FracOther - FracTCA) * StochTCOHTCE *
#(v1e)             (AMetLiv1 + FracLungSys*AMetLng))
#(v1e)             + (StochTCOHGluc * ARecircTCOG);
# Total in tissues
#(v1e)      TotTissueTCOH = ABodTCOH + ALivTCOH;
# Total metabolism of TCOH
    dt(AMetTCOHTCA) = RAMetTCOHTCA; #(vrisk)
#(v1e)    dt(AMetTCOHGluc) = RAMetTCOHGluc;
#(v1e)    dt(AMetTCOHOther) = RAMetTCOH;
#(v1e)      TotMetabTCOH = AMetTCOHTCA + AMetTCOHGluc + AMetTCOHOther;
# Mass balance
#(v1e)      MassBalTCOH = TotTCOHIn - TotTissueTCOH - TotMetabTCOH;

#**** Mass Balance for TCA
****************************************************
# Total production/intake of TCA
#(v1e)    dt(AOTCA) = kPOTCA;
#(v1e)      TotTCAIn = AOTCA + IVDoseTCA * BW
TotTCAProd =  (FracTCA*StochTCATCE*(AMetLiv1 + FracLungSys*AMetLng)) +
#(vrisk)
            (StochTCATCOH*AMetTCOHTCA);#(vrisk)#(vrisk)
# Total in tissues
#(v1e)      TotTissueTCA = APlasTCA + ABodTCA + ALivTCA;
      ## TotTissueTCA = ABodTCA + ALivTCA; #(v1h)
# Total metabolism of TCA
#(v1e)    dt(AMetTCA) = RAMetTCA;
# Mass balance
#(v1e)      TCADiff = TotTCAIn - TotTissueTCA - AMetTCA;
#(v1e)      MassBalTCA = TCADiff - AUrnTCA;

#**** Mass Balance for TCOG
***************************************************
# Total production of TCOG
#(v1e)      TotTCOGIn = StochGlucTCOH * AMetTCOHGluc;
# Total in tissues
#(v1e)      TotTissueTCOG = ABodTCOG + ALivTCOG + ABileTCOG;
# Mass balance
#(v1e)      MassBalTCOG = TotTCOGIn - TotTissueTCOG - ARecircTCOG -
AUrnTCOG;

#**** Mass Balance for DCVG
***************************************************
# Total production of DCVG
#(v1e)    dt(ADCVGIn) = (RAMetLiv2 + RAMetKid*(1-FracKidDCVC)) / MWTCE;
# Metabolism of DCVG
#(v1e)    dt(AMetDCVG) = RAMetDCVGmol;
# Mass balance
#(v1e)      MassBalDCVG = ADCVGIn - ADCVGmol - AMetDCVG;

#**** Mass Balance for DCVC
***************************************************
# Total production of DCVC
```

```
#(v1e)    dt(ADCVCIn) = RAMetDCVGmol * MWDCVC
#(v1e)            + RAMetKid * FracKidDCVC * StochDCVCTCE;
# Rate of DCVC eliminated (mg)#(v2.0)
#(v1e)      RAElimDCVC = kElimDCVC * ADCVC;#(v2.0)
# Dynamics for amount of DCVC eliminated (mg)#(v2.0)
#(v1e)      dt(AElimDCVC) = RAElimDCVC;#(v2.0)
# Mass balance#(v2.0)
#(v1e)      MassBalDCVC = ADCVCIn - ADCVC - AElimDCVC;#(v2.0)

#*********************************************************************
******
#***                        Dynamic Outputs
***
#*********************************************************************
******
# State Variables with dynamics:
# Input Variables:
#     none
# Other State Variables and Global Parameters:
# Temporary variables used:
# Temporary variables assigned:
# Notes:
#     INCOMPLETE
#*********************************************************************
******
# Amount exhaled during exposure (mg)
    dt(AExhExp) = (CInh > 0 ? RAExh : 0);

#*********************************************************************
******
#***                        Dose Metrics
***
#*********************************************************************
******
# State Variables with dynamics:
# Input Variables:
# Other State Variables and Global Parameters:
# Temporary variables used:
# Temporary variables assigned:
# Notes:
#     INCOMPLETE
#*********************************************************************
******
#**** AUCs in mg-hr/L
*****************************************************
#AUC of TCE in arterial blood
#(v1d)    dt(AUCCBld) = CArt;
#AUC of TCE in liver
#(v1d)    dt(AUCCLiv) = CLiv;
#AUC of TCE in kidney
#(v1d)    dt(AUCCKid) = CKid;
#AUC of TCE in fat
#(v1d)    dt(AUCCFat) = CFat;
#AUC of TCE in rapidly perfused
```

```
#(v1d)    dt(AUCCRap) = CRap;
#AUC of TCE in slowly perfused
#(v1d)    dt(AUCCSlw) = CSlw;
#AUC of TCE in gut
#(v1d)    dt(AUCCGut) = CGut;
#AUC of Chl in lung
#(v1d)      ChlFac = (StochChlorTCE / VMaxClear) * RAMetLng;
      # Steady-state concentration of Chloral (mg/L)
#(v1d)      CChl = (KMClear * ChlFac) / (1.0 - ChlFac);
#(v1d)    dt(AUCCChl) = CChl;
#AUC of TCOH in blood
#(v1d)    dt(AUCCTCOH) = CTCOH;
#AUC of free TCA in the plasma (mg/L * hr)
#(v1d)    dt(AUCPlasTCAFree) = CPlasTCAFree;
#AUC of total TCA in plasma (mg/L * hr)
#(v1d)    dt(AUCPlasTCA) = CPlasTCA;
#AUC of TCOG in blood
#(v1d)    dt(AUCCTCOG) = CTCOG;

#**********************************************************************
******
#**** Misc - Alternative models
*************************************
#**********************************************************************
******
#
## Alternative sampling scheme
## Priors on ViC are TruncNormal(0,1,-3,3)
#     MFat = BW * (Male == 0 ? 0.31667 : 0.19863) *
#           (1 + (Male == 0 ? 0.206 : 0.256) * VFatC);
#     LBM = BW * (1 - Mfat);
#     MLiv = LBM * (Male == 0 ? 0.03414 : 0.03078) *
#           (1 + (Male == 0 ? 0.102 : 0.093) * VLivC);
#     MTB = BW * 0.00018 *
#           (1 + 0.1 * VTBC);
#     MGut = (Male == 0 ? 0.02192 : 0.01959) *
#           (1 + (Male == 0 ? 0.092 : 0.078) * VGutC);
#     MRap = (Male == 0 ? 0.09925 : 0.10581) *
#           (1 + (Male == 0 ? 0.186 : 0.106) * VRapC);
#     MNp = LBM * (Male == 0 ? 0.26103 : 0.23241);
#     MSlw = BW - MFat - MLiv - MTB - MGut - MRap - MNp;
##    Convert to volume using density
#     VFat = MFat / 0.923;
#     VGut = MGut / 1.05;
#     VLiv = MLiv / 1.05;
#     VRap = MRap / 1.05;
#     VTB = MTB / 1.05;
#     VSlw = MSlw / 1.05;
#
#     VBld = (Male == 0 ? 0.688 : 0.725) * VRap; # Fraction of Rap
#       VPlas = FracPlas * VBld;
#     VBod = VFat + VGut + VRap + VTB + VSlw - VBld;
#           # for TCA -- body without liver or blood
#     VBodTCOH = VFat + VGut + VRap + VTB + VSlw;
```

```
#           # for TCOH and TCOG -- body without liver
#
## Revised Sampling of Flows
## Priors on QiC are TruncNormal(0,1,-3,3)
#     QFatCtmp = MFat / BW * (Male == 0 ? 0.268 : 0.252) *
#           (1 + 0.3 * QFatC);
#     QTBCtmp = MTB / BW * 140 *
#           (1 + 0.3 * QTBC);
#     QGutCtmp = MGut / BW * (Male == 0 ? 9.58 : 9.70) *
#           (1 + 0.3 * QLivC);
#     QLivCtmp = MLiv / BW * (Male == 0 ? 2.826 : 2.6) *
#           (1 + 0.3 * QLivC);
#     QRapCtmp = MRap / BW * (Male == 0 ? 3.81 : 3.67) *
#           (1 + 0.3 * QRapC);
#     QSlwCtmp = MSlw / BW * (Male == 0 ? 0.658 : 0.607) *
#           (1 + 0.3 * QLivC);
#     QtotCtmp = QFatCtmp + QTBCtmp + QGutCtmp + QLivCtmp +
#           QRapCtmp + QSlwCtmp;
#
## Alternative sampling scheme
#
#     QFat = QCnow * QFatCtmp / QtotCtmp;
#     QGut = QCnow * QGutCtmp / QtotCtmp;
#     QLiv = QCnow * QLivCtmp / QtotCtmp;
#     QSlw = QCnow * QSlwCtmp / QtotCtmp;
#     QTB = QCnow * QTBCtmp / QtotCtmp;
#     QRap = QCnow * QRapCtmp / QtotCtmp;
#     QGutLiv = QGut + QLiv;
#
#*************************************************************************
******
#***                    TCA Sub-model -- different protein binding
***
#*************************************************************************
******
# ## NOT YET TESTED
# In the original model, CBnd is constant while perfusing through tissue,
#     while CFree changes in response to CTissue.
# In this model, CBnd and CFree are in constant equilibrium while
perfusing,
#     so both CBnd and CFree change in response to CTissue.
# In both cases, tissue only exchanges with CFree, via partition
coefficient.
#
#     CVBodTCA = (BMax+kDissoc + CVBodTCAFree)*CVBodTCAFree /
#           (kDissoc + CVBodTCAFree);    # total in equilibrium
#    dt(ABodTCA) = QBodPlas * (CPlasTCA - CVBodTCA);
#     CVLivTCA = (BMax+kDissoc + CVLivTCAFree)*CVLivTCAFree /
#           (kDissoc + CVLivTCAFree);
#    dt(ALivTCA) = kPOTCA + QGutLivPlas*(CPlasTCA - CVLivTCA)
#                - RAMetTCA + (FracTCA * StochTCATCE * RAMetLiv1)
#                + (StochTCATCOH * RAMetTCOHTCA);
#*************************************************************************
******
```

```
#***                    TCE sub-model -- alternatives
***
#***********************************************************************
******
#******* Alternative local steady-state gas exchange assumption
***************
# ## TESTED - Makes very little difference, but does not add noticably
#     to computation burden
# VRaptmp and PRaptmp are the "original" parameters
# VRap = VRaptmp + VBld; #(v1a) -- now add blood to rapidly perfused
# Change VSlw so as to not double-count blood
# VBod = VFat + VGut + VRaptmp + VTB + VKid + VSlw; # For TCA #(v1a) --
doesn't include blood
# PRap = (PRaptmp*VRaptmp + VBld)/(VRaptmp + VBld); # Adjust PC
# Remove ABld from mass balance
#
#     CVen = (QFat*CVFat + QGutLiv*CVLiv + QSlw*CVSlw +
#           QRap*CVRap + QTB*CVTB + QKid*CVKid + kIV) / QCnow;
#
#**** Alternative local steady state TB region
********************************
# ## TESTED - Makes very little difference, but does not add noticably
#     to computation burden
# VRaptmp and PRaptmp are the "original" parameters
# VRap = VRaptmp + VTB; # add TB volume to rapidly perfused volume
# Change VSlw so as to not double-count blood
# VBod = VFat + VGut + VRaptmp + VTB + VKid + VSlw; # For TCA #(v1b)
don't double-count TB
# (v1b) temporary variable for Rapidly perfused blood flow
# QRapCtmp = 1 - QFatCtmp - QGutCtmp - QLivCtmp - QSlwCtmp - QTBCtmp -
QKidCtmp;
# PRap = (QRapCtmp+QTBCtmp)/(QRapCtmp/PRaptmp + QTBCtmp/PTB); #(v1b) --
now adjust PC for TB
#        QRap = QCnow - QFat - QGut - QLiv - QSlw - QKid; # (v1b) don't
subtract off TB
# Remove ATB from mass balance
# Remove CVTB from Blood mass balance
# Remove old definitions of CTB, CVTB
# Include RAMetLng metabolism in ARap differential equation
# Use local steady state approximation
#
# Venous blood draining TB
#     a = (VMaxClara/QTB + KMClara - CArt)/2;
#     b = CArt*KMClara;
#     CVTB = (b < 0.01*a*a ? b/2.0/a : sqrt(a*a+b)-a);
# Amount of TCE in the tracheo-bronchial region (mg)
#       CTB = CVTB * PTB;
#     ATB = CTB * VTB;
# Amount metabolized in the tracheo-bronchial region (mg)
#     a = (VMaxClara + QTB*(KMClara + CArt))/2;
#     b = CArt*QTB*VMaxClara;
#     RAMetLng = (b < 0.01*a*a ? b/2.0/a : a-sqrt(a*a-b));
#
```

```
#**** Alternative local steady state kidney
**********************************
# ## Probably not a good idea because kidney is target issue
#
# Similar procedure to TB
#
# Use local steady state approximation
#
# Venous blood draining Kid
#     a = (VMaxKidDCVG/QKid + KMKidDCVG - CArt)/2;
#     b = CArt*KMKidDCVG;
#     CVKid = (b < 0.01*a*a ? b/2.0/a : sqrt(a*a+b)-a);
# Amount of TCE in the kidney compartment (mg)
#       CKid = CVKid * PKid;
#     AKid = CKid * VKid;
# Amount metabolized in the kidney compartment (mg)
#     a = (VMaxKidDCVG + QKid*(KMKidDCVG + CArt));
#     b = CArt*QKid*VMaxKidDCVG;
#     RAMetKid = (b < 0.01*a*a ? b/2.0/a : a-sqrt(a*a-b));
#
#*****************************************************************
******
# OLD MODEL FOR ORAL DOSING -- no longer used
#AStom = Total - Abs; # OLD
#dt(Abs) = (kAS * AStom) + (kTSD * AStom); # OLD
#**** Alternative model with diffusion-limited fat -- not used
****************
#     #AFatBld,   # Amount in fat blood -- for diffusion-limited fat
# Amount of TCE in fat blood (mg)
#                 dt(AFatBld) = (QFat * (CArt - AFatBld/VFatBld)) +
#                 (PAFat2 * (CFat/PFat)) - (PAFat1 * AFatBld/VFatBld);
#                 dt(AFat) = (PAFat1 * CVFat) - (PAFat2 * (CFat / PFat));
# Total amount in fat blood and fat tissue (mg)
#                 ATotFat = AFatBld + AFat;


};
################ End of Dynamics ####################################


CalcOutputs{



#**** Static outputs for comparison to data
**********************************
# TCE
#(v1.2)     AlvRet = (InhDose > 0 ? 1 - AExhExp/InhDose : 1); #
      RetDose = ((InhDose-AExhExp) > 0 ? (InhDose - AExhExp) : 1e-15); #
      CAlvPPM = (CAlv < 1.0e-15 ? 1.0e-15 : CAlv * (24450.0 / MWTCE));
      CInhPPM = (ACh< 1.0e-15 ? 1.0e-15 : ACh/VCh*24450.0/MWTCE);
            # CInhPPM Only used for CC inhalation
#(v1.2)     CMixExh = (0.7 * CAlv) + (0.3 * CInh);
      CArt = (CArt < 1.0e-15 ? 1.0e-15 : CArt);
```

```
      CVen = (CVen < 1.0e-15 ? 1.0e-15 : CVen);
      CBldMix = (CArt+CVen)/2;
      CFat = (CFat < 1.0e-15 ? 1.0e-15 : CFat);
      CGut = (CGut < 1.0e-15 ? 1.0e-15 : CGut);
      CRap = (CRap < 1.0e-15 ? 1.0e-15 : CRap);
      CSlw = (CSlw < 1.0e-15 ? 1.0e-15 : CSlw);
#(v1.2)     CTB = (CTB < 1.0e-15 ? 1.0e-15 : CTB);
      CHrt = CRap;
      CKid = (CKid < 1.0e-15 ? 1.0e-15 : CKid);
      CLiv = (CLiv < 1.0e-15 ? 1.0e-15 : CLiv);
      CLung = CRap; #(v1.2)
      CMus = (CSlw < 1.0e-15 ? 1.0e-15 : CSlw);
      CSpl = CRap;
      CBrn = CRap;
      zAExh = (AExh < 1.0e-15 ? 1.0e-15 : AExh);
      zAExhpost = ((AExh - AExhExp) < 1.0e-15 ? 1.0e-15 : AExh -
AExhExp);
# TCOH
      CTCOH = (CTCOH < 1.0e-15 ? 1.0e-15 : CTCOH);
      CBodTCOH = (ABodTCOH < 1.0e-15 ? 1.0e-15 : ABodTCOH/VBodTCOH);
      CKidTCOH = CBodTCOH;
      CLivTCOH = (ALivTCOH < 1.0e-15 ? 1.0e-15 : ALivTCOH/VLiv);
      CLungTCOH = CBodTCOH;
# TCA
      CPlasTCA = (CPlasTCA < 1.0e-15 ? 1.0e-15 : CPlasTCA);
      CBldTCA = CPlasTCA*TCAPlas;
      CBodTCA = (CBodTCA < 1.0e-15 ? 1.0e-15 : CBodTCA);
      CLivTCA = (CLivTCA < 1.0e-15 ? 1.0e-15 : CLivTCA);
      CKidTCA = CBodTCA;
      CLungTCA = CBodTCA;
      zAUrnTCA = (AUrnTCA < 1.0e-15 ? 1.0e-15 : AUrnTCA);
      zAUrnTCA_sat = (AUrnTCA_sat < 1.0e-15 ? 1.0e-15 : AUrnTCA_sat);
      zAUrnTCA_collect = (AUrnTCA_collect < 1.0e-15 ? 1.0e-15 :
AUrnTCA_collect);
# TCOG
      zABileTCOG = (ABileTCOG < 1.0e-15 ? 1.0e-15 : ABileTCOG);
      #  Concentrations are in TCOH-equivalents
      CTCOG = (CTCOG < 1.0e-15 ? 1.0e-15 : CTCOG);
      CTCOGTCOH = StochTCOHGluc*CTCOG;
      CBodTCOGTCOH = (ABodTCOG < 1.0e-15 ? 1.0e-15 :
StochTCOHGluc*ABodTCOG/VBodTCOH);
      CKidTCOGTCOH = CBodTCOGTCOH;
      CLivTCOGTCOH = (ALivTCOG < 1.0e-15 ? 1.0e-15 :
StochTCOHGluc*ALivTCOG/VLiv);
      CLungTCOGTCOH = CBodTCOGTCOH;
      AUrnTCOGTCOH = (AUrnTCOG < 1.0e-15 ? 1.0e-15 :
StochTCOHGluc*AUrnTCOG);
      AUrnTCOGTCOH_sat = (AUrnTCOG_sat < 1.0e-15 ? 1.0e-15 :
StochTCOHGluc*AUrnTCOG_sat);
      AUrnTCOGTCOH_collect = (AUrnTCOG_collect < 1.0e-15 ? 1.0e-15 :
StochTCOHGluc*AUrnTCOG_collect);
# Other
      CDCVGmol = (CDCVGmol < 1.0e-15 ? 1.0e-15 : CDCVGmol);
      CDCVGmol0 = CDCVGmol;
```

```
      CDCVG_NDtmp = CDFNormal(3*(1-CDCVGmol/CDCVGmolLD));
            # Assuming LD = 3*sigma_blank, Normally distributed
      CDCVG_ND = ( CDCVG_NDtmp < 1.0 ? ( CDCVG_NDtmp >= 1e-100 ? -
log(CDCVG_NDtmp) : -log(1e-100)) : 1e-100 );
# DCVC#(v2.0)
      CDCVCmol = (CDCVCmol < 1.0e-15 ? 1.0e-15 : CDCVCmol);#(v2.0)
      CDCVCmol0 = CDCVCmol;#(v2.0)
      CDCVC_NDtmp = CDFNormal(3*(1-CDCVCmol/CDCVCmolLD)); #(v2.0)
            # Assuming LD = 3*sigma_blank, Normally distributed#(v2.0)
      CDCVC_ND = ( CDCVC_NDtmp < 1.0 ? ( CDCVC_NDtmp >= 1e-100 ? -
log(CDCVC_NDtmp) : -log(1e-100)) : 1e-100 );#(v2.0)
# DCA#(v2.0)
      CDCAmol = (CDCAmol < 1.0e-15 ? 1.0e-15 : CDCAmol);#(v2.0)
      CDCAmol0 = CDCAmol;#(v2.0)
      CDCA_NDtmp = CDFNormal(3*(1-CDCAmol/CDCAmolLD)); #(v2.0)
            # Assuming LD = 3*sigma_blank, Normally distributed#(v2.0)
      CDCA_ND = ( CDCA_NDtmp < 1.0 ? ( CDCA_NDtmp >= 1e-100 ? -
log(CDCA_NDtmp) : -log(1e-100)) : 1e-100 );#(v2.0)
         #
      AUrnTCTotMole = zAUrnTCA / MWTCA + AUrnTCOGTCOH / MWTCOH;
      TotCTCOH = CTCOH + CTCOGTCOH;
      TotCTCOHcomp = CTCOH + CTCOG; # ONLY FOR COMPARISON WITH HACK
      ATCOG = ABodTCOG + ALivTCOG; # ONLY FOR COMPARISON WITH HACK
# Misc
      CVenMole = CVen / MWTCE;
      CPlasTCAMole = (CPlasTCAMole < 1.0e-15 ? 1.0e-15 : CPlasTCAMole);
      CPlasTCAFreeMole = (CPlasTCAFreeMole < 1.0e-15 ? 1.0e-15 :
CPlasTCAFreeMole);

#**** Dose Metrics
************************************************************
# Oxidation/BW
#(v1d)      AMetLiv1BW = AMetLiv1 / BW;

AMetOX = AMetLiv1 + AMetLng;#(vrisk)
AMetGSH = AMetLiv2 + AMetKid;#(vrisk)

};
```
